# Heterogeneous Trimetallic Nanoparticles as Catalysts

**DOI:** 10.1021/acs.chemrev.1c00493

**Published:** 2022-03-09

**Authors:** James W. M. Crawley, Isla E. Gow, Naomi Lawes, Igor Kowalec, Lara Kabalan, C. Richard A. Catlow, Andrew J. Logsdail, Stuart H. Taylor, Nicholas F. Dummer, Graham J. Hutchings

**Affiliations:** † Max Planck−Cardiff Centre on the Fundamentals of Heterogeneous Catalysis (FUNCAT), Cardiff Catalysis Institute, School of Chemistry, Cardiff University, Main Building, Park Place, Cardiff CF10 3AT, United Kingdom; ‡ UK Catalysis Hub, Research Complex at Harwell, Rutherford Appleton Laboratory, Didcot OX11 OFA, U.K.; § Department of Chemistry, University College London, Gordon Street, London WC1H 0AJ, U.K.

## Abstract

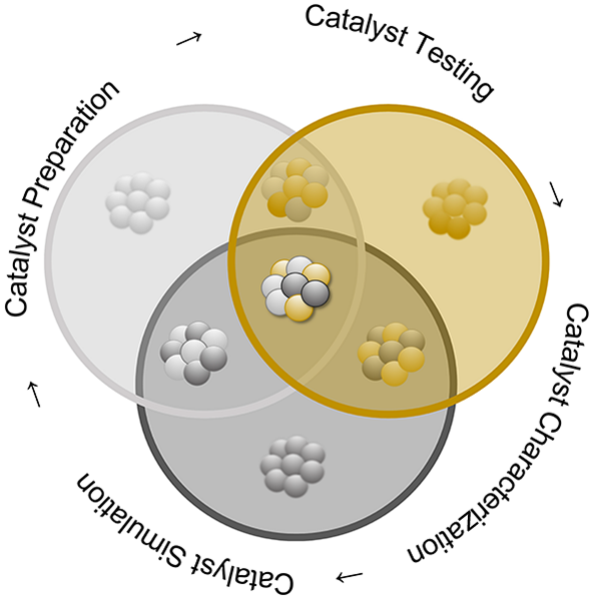

The development and application of trimetallic nanoparticles continues to accelerate rapidly as a result of advances in materials design, synthetic control, and reaction characterization. Following the technological successes of multicomponent materials in automotive exhausts and photovoltaics, synergistic effects are now accessible through the careful preparation of multielement particles, presenting exciting opportunities in the field of catalysis. In this review, we explore the methods currently used in the design, synthesis, analysis, and application of trimetallic nanoparticles across both the experimental and computational realms and provide a critical perspective on the emergent field of trimetallic nanocatalysts. Trimetallic nanoparticles are typically supported on high-surface-area metal oxides for catalytic applications, synthesized *via* preparative conditions that are comparable to those applied for mono- and bimetallic nanoparticles. However, controlled elemental segregation and subsequent characterization remain challenging because of the heterogeneous nature of the systems. The multielement composition exhibits beneficial synergy for important oxidation, dehydrogenation, and hydrogenation reactions; in some cases, this is realized through higher selectivity, while activity improvements are also observed. However, challenges related to identifying and harnessing influential characteristics for maximum productivity remain. Computation provides support for the experimental endeavors, for example in electrocatalysis, and a clear need is identified for the marriage of simulation, with respect to both combinatorial element screening and optimal reaction design, to experiment in order to maximize productivity from this nascent field. Clear challenges remain with respect to identifying, making, and applying trimetallic catalysts efficiently, but the foundations are now visible, and the outlook is strong for this exciting chemical field.

## 1 Introduction

Nanoparticles (NPs) have been utilized for millennia, as evidenced by their discovery in cave paintings^1^ their presence in the Lycurgus cup from the fourth century^2^ and in glassware dating back to the late Bronze Age.^3^ A notable modern focus in nanotechnology emerged in the late 20th century,^2^ where the application of NPs has broadened; considerable research has also been performed since this period in the development of nanotubes, -wires, -fibers, porous materials, quantum dots, and dendrimers.^4−11^ Because of the large surface-to-volume ratio of NPs compared with bulk materials, NPs are attractive, but not limited to, the catalysis industry. NPs are also notable across varied industries such as transport, food, textiles, and personal care products. For example, the catalytic converters present in the exhaust systems of motor vehicles are a widely known application of multimetal NPs. Comprising Pd/Pt, Rh, CeO_2_, and Al_2_O_3_, catalytic converters are crucial in reducing and controlling the release of toxic gases and pollutants.^12^ Self-cleaning surfaces based on lanthanum doped titania^13^ and indium-doped tin oxide for photovoltaic thin films in solar panels^14^ are other applications of multicomponent materials.

Specific focus on trimetallic nanoparticles (TMNPs) in catalysis is an emerging area, one developed from the benefits observed upon addition of a second metal to NP catalysts, such as Pd to Au NPs supported on a high-surface-area metal oxide.^15,16^ In depth studies of bimetallic catalysts have been explored; therefore, the fundamental understanding of these systems can be utilized in the design, synthesis, characterization, and application of trimetallic catalysts. Bimetallic catalysts are well-known to exhibit different properties from their monometallic counterparts. Synergistic effects between the two metals are often responsible for the increased activity, selectivity, and stability. Ferrando *et al.* discussed several crucial considerations when mixing two metals,^17^ which include (i) the relative bond strength between two metals, (ii) the surface energy, (iii) relative atomic sizes, (iv) geometric effects, and (v) electronic effects. Sankar *et al.* presented a thorough and critical review of bimetallic catalysts, providing an overview of the recent developments in material design, synthesis methods, characterization, and catalytic applications.^18^

Differences in electronic and geometric properties are among the most common explanations why bimetallic catalysts often show increased catalytic performances. Xie *et al.* demonstrated the positive influences of adding a second metal to Ni.^19^ They reported an electronic effect that in this system weakened the bonding of CO, suppressing methanation for aqueous-phase reforming of ethylene glycol. Additionally, the formation of a SnNi bimetallic alloy was shown through X-ray diffraction (XRD) and X-ray photoelectron spectroscopy (XPS) and was suggested as a center for H_2_O activation where surface hydroxyls are formed on Sn, facilitating higher H_2_ selectivity.^19^ Ferretti *et al.* used XPS to suggest a partial electron transfer when a binding energy shift for the Rh 3d_5/2_ peak was observed upon Sn addition relative to monometallic Rh. The successful application of a SnRh/SiO_2_ catalyst to the hydrogenation of crotonaldehyde was reported.^20^ The addition of a third metal adds to the complexity of the system, but key principles learned and understood from bimetallic systems can be directly applied to aid the synthesis of trimetallic catalysts. In particular, advanced characterization techniques are relevant to both bi- and trimetallic systems, such as high-resolution electron microscopy, including high-angle annular dark-field scanning transmission electron microscopy (HAADF-STEM), and X-ray adsorption techniques such as X-ray absorption fine structure (XAFS). Significantly, the distinction of the different elements present in a solid solution, core@shell, or segregated mixed system should be explored when possible to provide a better understanding of the complex reaction site. For example, distinguishing the concentration of elements present on the surface of a bimetallic supported catalyst was reported by Rioux and Vannice through chemisorption studies.^21^ Chemisorption of CO, N_2_O, and H_2_ was used to probe the surface concentration of carbon-supported Pt–Cu catalysts in comparison with their bulk compositional values and to differentiate observations in the rate of isopropyl alcohol dehydrogenation. Zhu *et al.*(22) applied a multitude of characterization techniques, including XPS, high-resolution transmission electron microscopy (HRTEM), and high-sensitivity low-energy ion scattering (LEIS) spectroscopy to differentiate between structural variations. LEIS spectroscopy was employed to investigate the surface atomic composition of the catalysts, including bimetallic NiCo and RuNiCo TMNPs, upon exposure to different heat treatments. Tarditi and Cornaglia^23^ also utilized LEIS spectroscopy to investigate surface segregation of novel PdAgCu ternary alloys. It is known that the chemical and physical properties of the bulk composition vary from those of metallic alloy surfaces. Such techniques are encouraged to determine the role of metal alloys at the surface as well as their bulk compositions. Realizing the full benefits of using trimetallic compositions as catalysts requires advanced characterization concurrent with building on the successful application of these complex materials.

**Figure 1:**
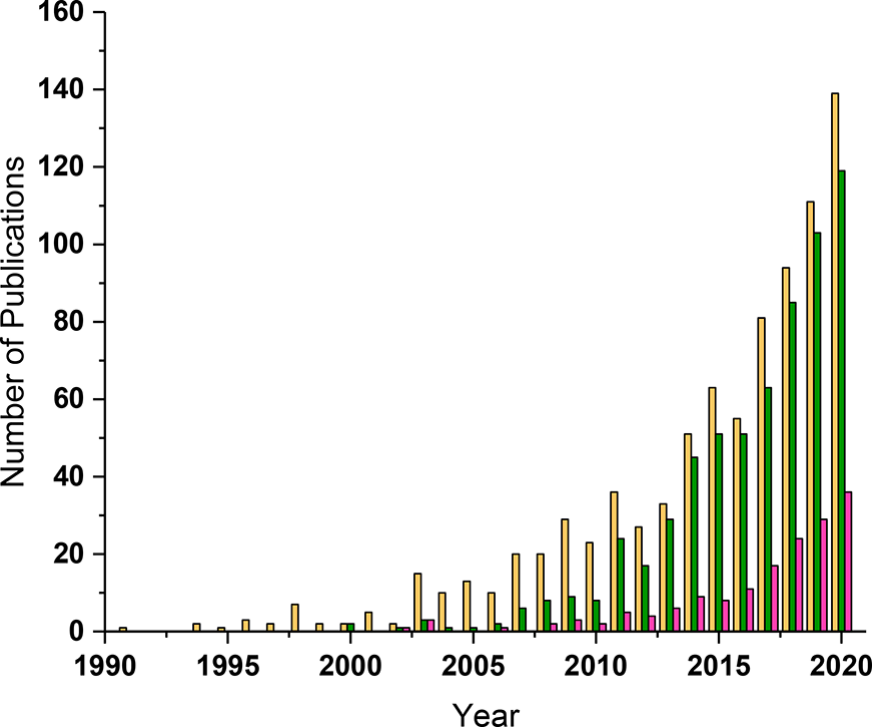
Number of publications based on a Web of Science (February 2021) search for “trimetallic catalyst” (tan), “trimetallic nanoparticles” (green), and “trimetallic nanoparticles” with “applications” (pink) and limited from 1990 to 2020.

**Figure 2:**
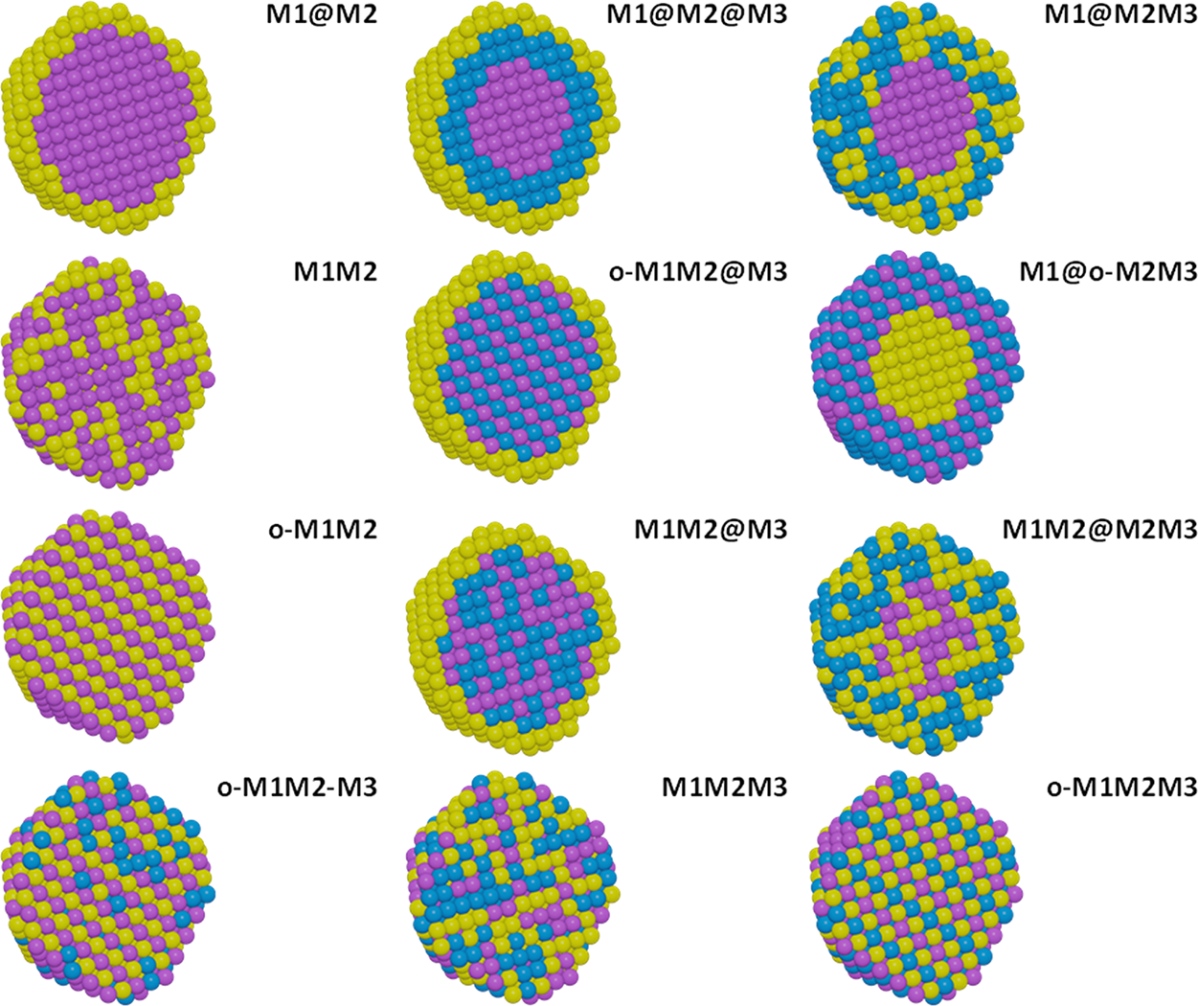
Diagrammatical representations of different compositional descriptors used in multicomponent nanomaterials (left to right) noting ordered structures (o-) where appropriate: bimetallic core@shell (M1@M2), trimetallic inner-core@core@shell (M1@M2@M3), core@random-shell (M1@M2M3), random alloy (M1M2), ordered-core@shell (o-M1M2@M3), core@ordered-shell (M1@o-M2M3), ordered alloy (o-M1M2), random-core@shell (M1M2@M3), core@shell where M2 is distributed across the particle (M1M2@M2M3, often described as an AB@AC structure), ordered alloy with M3 randomly distributed (o-M1M2-M3), random trimetallic solid solution (M1M2M3), and ordered trimetallic alloy or intermetallic solution (o-M1M2M3). Where a support is used, the nomenclature will be given as M1M2M3/support (*e.g.*, PtPdAu/TiO_2_).

Over 130 articles related to TMNP catalysts were published in 2020 (Web of Science; search “trimetallic catalyst”), with an upward trend in publications over the past decade (Figure 1) that is far greater than the general growth in scientific publications.^24^ Consequently, TMNPs are considered to be a key component in the advancement of catalyst-based processes. However, fundamental studies on TMNP catalysts remain elusive, and much work will be required to truly advance this field. Despite this, TMNPs have been successfully applied as catalysts to alcohol oxidation^25,26^ and dehydrogenation^27,28^ reactions, where increased catalytic activity over mono- and bimetallic counterparts has been reported. Therefore, TMNPs can be considered highly promising, as the addition of a third metal often synergistically enhances the catalytic performance. However, the catalytic activity is highly dependent on the composition, morphology, and dimensions, and thus, it is necessary to apply multiple characterization techniques to understand the nature of TMNPs. In this respect, computational approaches have become a vital component in the development of TMNP catalysts, particularly in defining the expected stability of a given composition.^29−31^ As the growth of reported bi- and trimetallic materials has increased, so too has the number of terms used to describe these systems, principally because of their complexity. Terms such as solid solution, core–shell, intermetallic solution, ordered alloy, and segregated systems are abundant and often used in relation to the intended composition or when suitable characterization techniques can resolve the elemental composition and structure of a nanoparticle. To further complicate matters, this structure may not be applicable to all nanoparticles, so a larger pool of candidates must be considered. We have attempted to summarize the variety of compositional descriptors in Figure 2, and these terms are then used throughout the review and should be referenced when distinguishing the systems commonly reported. These include core@shell-types, such as M1@M2@M3/support, and solid solutions or alloys, such as M1M2M3/support, where M represents a metal. Furthermore, where appropriate, the nature of the nanoparticle structure can be described through the use of a descriptor to notate order, *e.g.*, o-M1M2M3. Where this is not used, a random structure can be assumed, *e.g.*, M1M2M3. Defining these clearly and consistently across the variety of the fields of which TMNPs are part should ideally be standardized to avoid confusion.

Understanding the operation and benefit of TMNPs as catalytic active sites is challenging because of the additional degree of freedom of ternary metal nanoparticles. Synergy is often broadly used to describe the benefit of additional metals; however, this term is not as clear as it could be in terms of molecular transformations through electron transfer on supported nanoparticle surfaces. Bond breaking and making are crucial processes in catalysis, and the d-band model has been an impactful development to describe how the electronic properties of a material influence bond formation at the surface. In the d-band model, the strength of a surface–adsorbate interaction is determined by the degree of electronic occupation in the hybridized d−σ antibonding states that form between the surface d and adsorbate σ orbitals.^32^ The degree of occupation in the surface d states is element- and/or composition-dependent, and higher occupancy leads to greater occupancy of the surface–adsorbate d−σ antibonding states, which results in weaker surface–adsorbate interactions. Thus, the center of the d states (or the d-band center for short), which signifies the occupancy of the surface d states, has become synonymous with the strength of any surface–adsorbate interactions. In the case of TMNPs, such considerations are highly important because of the potential tunability of the position of the d-band center by compositional variation.^33^

This critical review attempts to provide insight and understanding of TMNP material descriptions, preparation methods, and catalytic activities, particularly emphasizing the positive influence that TMNPs are envisaged to have on the future of catalyst-based industries.

## 2 Properties of Trimetallic Nanoparticles

When nanoparticles are being synthesized for catalytic applications, there are several factors to consider, including size, shape, morphology, structure, composition, phases, supports, and defects. To date, a plethora of studies involving mono- and bimetallic nanoparticles (BNMPs) have taken these factors into account in the careful design of systems for catalytic applications.^18,34−37^ For trimetallic nanoparticles, the same fundamental principles can be applied. These considerations become even more complex because of the increased degrees of freedom; however, the greater flexibility afforded with respect to tuning these properties can greatly improve catalytic performance. Where possible, we have attempted to relate the various catalytic processes discussed according to the rate of product formation or turnover frequency (TOF) to better compare catalyst compositions. This is particularly key when attempting to gauge the efficacy of a TMNP catalyst compared to their mono- or bimetallic counterparts. Often this is a challenge because of the lack of complementary specific activity data given in the literature. In this case we have used the available data such as catalyst mass or volume, supported metal loading (mass or moles), flow rate, or gas hourly space velocity and calculated the activity as moles of product per unit catalyst mass per hour or as TOF (moles of product per unit time as a function of moles of metal present on the catalyst). Ideally, productivity values should be calculated at moderate conversion percentages on the basis of active site density, as the productivity is insensitive when taken at high conversions. However, the values should offer the reader a way to initially assess the influence of metal composition. Therefore, the productivity values are presented in tables in the following sections for comparison where appropriate. We submit that through these metrics we can compare catalysts for a given process, but care must be taken because it is rare to possess activity rate data for a range of compositions of BMNPs and TMNPs within one study. Therefore, we take this opportunity to make the point that activity rate data should be provided routinely, normalized to metal loading as a function of time, to better understand the improvements that are often claimed for TMNP catalysts.

### 2.1 Key Properties of TMNPs to Consider

The composition of a metal nanoparticle is an important consideration that includes the identities of the species present in the nanoparticle in addition to the ratio of these species. In a trimetallic nanoparticle, the metal ratio A:B:C can have drastic implications for the morphology, structure, and performance of a catalyst, which have been explored through both synthetic and computational studies. The key properties of composition and morphology will be introduced here.

**Figure 3:**
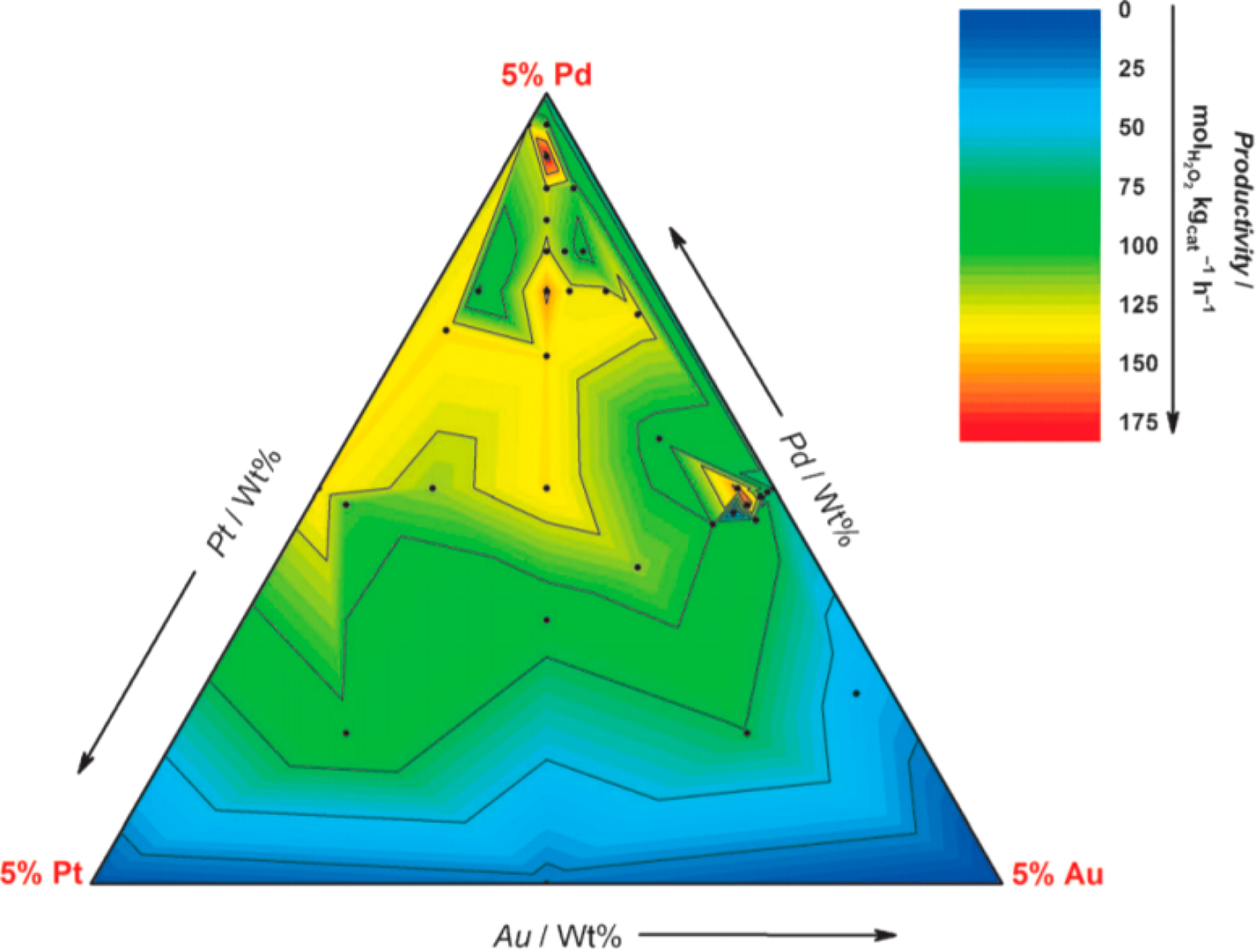
Contour diagram of catalytic activity of AuPdPt/CeO_2_ catalysts, showing how the rate of H_2_O_2_ production (mol_H_2_O_2__ kg_cat_^–1^ h^–1^) depends on the Au:Pd:Pt ratio. Adapted with permission from ref (16). Copyright 2014 Wiley-VCH.

As an example, Figure 3 illustrates the importance of composition, specifically the ratio of the three metals on the resultant catalytic activity.^16^ Hutchings and co-workers developed a contour plot of the rate of hydrogen peroxide productivity as a function of the Au:Pd:Pt metal ratio. The most active catalysts were those with a small amount of Pt relative to an Au:Pd weight ratio of 1:1 (Au:Pd:Pt = 2.4:2.4:0.2). Where the Pd or Pt concentration was higher, the hydrogenation rate was significantly increased, reducing the overall peroxide yield. To generate this plot, many experiments were carried out with various compositional ratios, and the results show that a small change in composition can have a significant impact on the overall reaction rate. Presently, this complexity is a key issue in the preparation and application of TMNPs.

The influence of the metal nanoparticle (MNP) oxidation state on the catalytic performance can be significant. Metals within a nanoparticle system are not necessarily of neutral charge, which may impact the outcome of reactions because of the altered electronic interactions between the metal and the substrates. For example, different oxidation states change the redox properties of the nanoparticle, which in turn may enhance or suppress its performance.^38^ To illustrate this phenomenon, we use a monometallic example from the work of Hutchings *et al.*(39) on the mechanism of acetylene hydrochlorination to vinyl chloride over single-site Au/C catalysts. These catalytic sites were investigated using *in situ* XAFS and XPS. According to analysis of these measurements, the active form of gold under the reaction conditions was not Au^0^ but rather Au cation sites, specifically, Au^1+^ or Au^3+^. The results indicated the catalytic activity was strongly correlated with the Au^1+^:Au^3+^ ratio, with Au^1+^ showing high catalytic activity and the Au^0^ and Au^3+^ sites exhibiting little catalytic activity. Interestingly, the catalyst shows a 1 h induction period of modest activity, suggesting that the active Au^1+^ form of the catalyst is formed under the reaction conditions. In a multimetallic system, the oxidation state of just one of the three metals may impact the catalytic performance.^40,41^ For example, Sial *et al.* noted that in their PtCoFe catalysts, a high ratio of Pt^0^ to oxidized Pt^2+^ vastly improved the electrocatalytic performance for ethanol oxidation and oxygen reduction.^42^ Analysis of XPS data revealed that the other metals were present as Co^2+^ and Fe^0^, and in addition to the strong structural character of this composition, these factors facilitated the enhanced catalytic activity observed.

**Figure 4:**
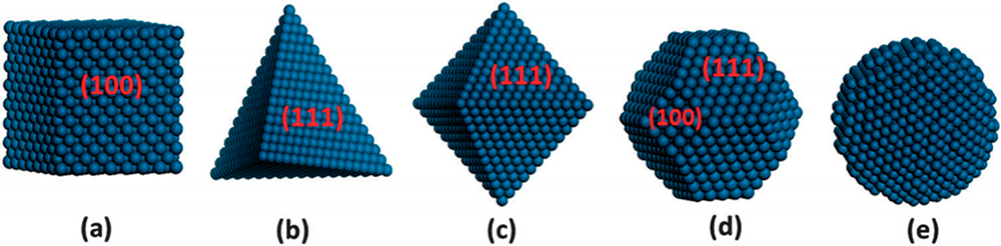
Examples of metal NPs with different morphologies: (a) cube with (100) facets, (b) tetrahedron with (111) facets, (c) octahedron with (111) facets, (d) cuboctahedron with (100) and (111) facets, and (e) a spherical shape. Adapted with permission from ref (43). Copyright 2016 Royal Society of Chemistry.

Morphology is another crucial factor to consider when assessing catalytic performance. Many examples of size- and shape-dependent catalytic performances have been reported, including recent literature reviews that describe the influences of size, shape, and support on CO oxidation reactions.^43−46^ In the case of TMNPs, the addition of a third metal can be more complicated but can result in greater flexibility and control. Metal NP preparation methods influence the different shape formations and the various ways atoms can pack together.^6,47^ Darby *et al.* explored this concept through computational modeling of bimetallic Au clusters in which one Au atom was replaced by a Cu atom.^48^ Their calculations indicated that replacement of one Au atom could lead to significant structural changes for clusters of 14, 16, and 55 atoms compared with the monometallic counterparts. Figure 4 illustrates various model particle shapes such as (a) cubic, (b) tetrahedral, (c) octahedral, (d) truncated octahedral, and (e) spherical.^43^

Miller indices (*hkl*) are often used to categorize surfaces of NPs, and for a metal catalyst with a face-centered-cubic (FCC) lattice, the (100), (111), and (110) planes are the main surfaces of a shape-controlled NP.^43^ The different surfaces, with different coordination numbers (CNs), influence the adsorption, dissociation, coupling, and interaction of intermediate adsorbates in a catalytic event. Selectivity is also sensitive to the arrangement of atoms and exposed facets on the surface.^49^ For example, the difference between the adsorption energies of the Pt(111) and Pt(100) surfaces can be traced to their intrinsic structural differences and the difference in binding energies, with density functional theory (DFT) studies showing that the binding energy of NH_*x*_ species on the Pt(100) surface is greater than that on Pt(111) surface by *ca.* 0.7 eV.^50^ This difference between the adsorption energies of adsorbates on the two surfaces probably results from the difference in coordination numbers of Pt atoms on the Pt(111) and Pt(100) facets. A shape-dependent catalytic reaction for the hydrogenation of benzene over Pt NPs was reported by Somorjai *et al.*, who synthesized cubic and cuboctahedral NPs shapes using tetradecyltrimethylammonium bromide as a capping agent.^51^ The cubic particles consisted of Pt(100) facets (*ca.* 12.3 nm diameter), whereas the cuboctahedral particles contained both Pt(100) and Pt(111) facets (*ca.* 13.5 nm diameter). The hydrogenation of benzene over Pt NPs was reported to be highly dependent on the shape; it was concluded that the Pt(111) surface of the cuboctahedral NPs resulted in cyclohexene production, as both possible products (cyclohexane and cyclohexene) formed on the cuboctahedral nanoparticles whereas only cyclohexane was produced on the cubic nanoparticles. On Pt(111), the weak adsorption energy of cyclohexene makes desorption of cyclohexene facile, whereas the selectivity for cyclohexane on Pt(100) was considered to be a result of the high adsorption energy and the relatively strong binding of cyclohexene, encouraging further hydrogenation. Hence, the cuboctahedral Pt nanoparticle catalysts, containing both (111) and (100) exposed facets, resulted in both cyclohexene and cyclohexane formation.

**Figure 5:**
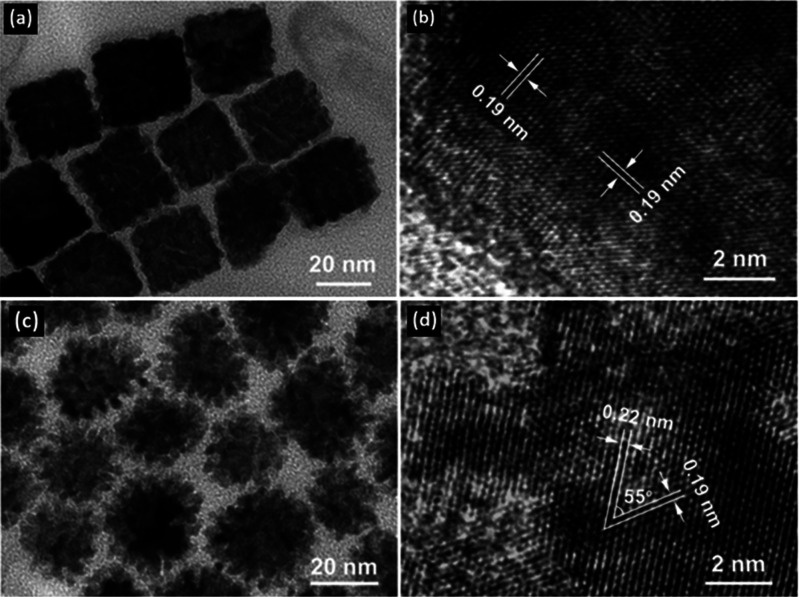
(a) TEM image of PtPdCu porous nanocubes. (b) HRTEM image of PtPdCu porous nanocubes. (c) TEM image of PtPdCu porous nanodendrites. (d) HRTEM image of PtPdCu porous nanodendrites. Adapted from ref (47). Copyright 2019 American Chemical Society.

Wang *et al.* demonstrated a shape-controlled synthesis of trimetallic PtPdCu nanocrystals applied as electrocatalysts for the methanol oxidation reaction (MOR).^47^ The different surfactants used in the coreduction synthesis, cetyltrimethylammonium bromide (CTAB) and cetyltrimethylammonium chloride (CTAC), resulted in the unique formation of nanocubes and nanodendrites, respectively. According to Xu *et al.*, the stabilization of the Pd(100) facets was due to binding of the Br^–^ ions from CTAB, promoting the formation of Pd nanocubes. When CTAB was replaced with CTAC, the chloride ions did not efficiently stabilize and promote the formation of (100) surfaces of Pd.^52^ Similar reasoning was considered to account for the different nanocrystal formations reported by Wang *et al.*(47) The materials characterized by TEM displayed the sizes and morphologies of the PtPdCu porous nanocubes and nanodendrites. The lattice fringe measurements shown in HRTEM images can be assigned to the (200) crystal planes of PtPdCu alloy in the nanocubes (Figure 5b) and the (111) and (200) crystal planes of PtPdCu alloy in the nanodendrites (Figure 5d). Alloy formation was also observed in a shift in the XRD pattern and a positive binding energy shift in the Pd 3d_5/2_ peak in the XPS data, providing evidence of the PtPdCu alloy compared with monometallic Pd. HAADF-STEM images with elemental mapping also confirmed alloy formation but additionally indicated a Pt-rich surface in the nanocubes. The PtPdCu nanocrystals exhibited higher electrochemical activity for MOR in comparison with a commercial Pt/C catalyst. The mass activity and specific activity of the catalysts were reported to be in the order of PtPdCu nanodendrites > PtPdCu porous nanocubes > commercial Pt/C. This trend could be related to the higher electrochemically active surface area (ECSA) of the PtPdCu nanodendrites (75 m^2^ g_Pt_^–1^) in comparison with the PtPdCu nanocubes (50 m^2^ g_Pt_^–1^) and commercial Pt/C catalyst (34 m^2^ g_Pt_^–1^). The PtPdCu nanocrystals have a higher ECSA because of their porous morphology compared with the monometallic Pt/C catalyst, resulting in more active sites. Therefore, the PtPdCu nanocrystals exhibit higher activity due to the combination of the porous morphology and the synergistic effects from adding Pd and Cu atoms. The addition of Cu atoms may modify the electronic state of Pt atoms, resulting in a weaker Pt–CO bond during MOR, decreasing catalytic poisoning by CO.^47^

In the field of nanoparticle catalysis, surface defect sites have long been known to exhibit enhanced catalytic performance. Such surface defects include atomic steps, kinks, and edges.^53^ These defect sites contain a high concentration of low-coordinate atoms, which may facilitate catalysis of certain processes. For example, the cleavage of N_2_ readily occurs on coordination-unsaturated Ru sites.^54^ The enhancement of these catalytic sites in bond-breaking steps can be attributed to local electronic or geometric effects.^55,56^ Because of the localized change in electronic and geometric structure, defect sites are more susceptible to act as nucleation sites, allowing for site-selective addition of the third metal around the sites.

Li *et al.*(26) reported a defect-dominated shape recovery phenomenon of nanocrystals. Fine-tuned geometric and electronic structures were rationally designed by means of a step-induced growth mechanism. Two key requirements were reported for the synthesis of new trimetallic o-M1M2@M3 structures by shape recovery: the metals must have the same crystal structure and similar atomic radii. This preparative concept involves chemical etching of an Pt_3_Ni octahedral crystal to form a defect-rich concave structure.^26^ Other defect-rich trimetallic structures obtained using etching methods have been reported. These include nanoframes, which are hollow and retain only the atoms on the edges of the facets. Nanoframes possess a high surface area and many low-coordinate defects that are known to be highly catalytically active. This structural motif along with the trimetallic composition has been shown to further improve catalytic performance.^57−62^

Perhaps one of the most compelling properties of multimetallic NPs is the many structures they can manifest. TMNPs with varied morphologies, such as wires,^63−67^ dumbbells,^68^ hollow interiors,^5,69−71^ nanotubes,^72,73^ and dendrimers,^74−76^ have been reported, exemplifying the flexible outcomes available through variation of the synthesis conditions and metal choice. Interestingly, the different metals within NPs can be distributed in many ways on and within the nanoparticle. Therefore, with respect to TMNPs, many structures can be investigated, such as random alloys and core–shell structures, similar to those seen in bimetallic systems.

**Figure 6:**
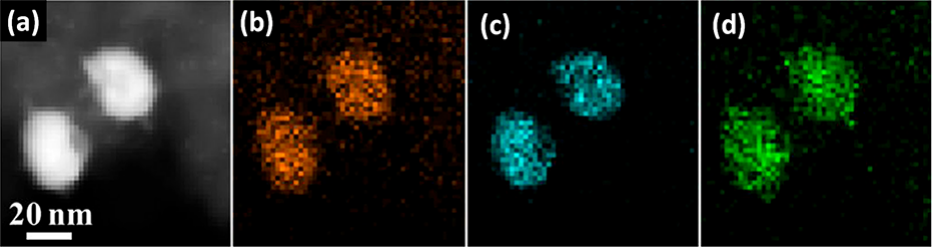
(a) HAADF-STEM image of Ni_40_Au_15_Pd_45_/C and (b–d) the corresponding EDX elemental maps of (b) Ni, (c) Au, and (d) Pd. Adapted with permission from ref (77). Copyright 2014 Elsevier.

Alloyed TMNPs can exhibit an essentially random distribution of the three metals within the nanoparticle, with no clear structural segregation present. For example, Wang *et al.* prepared NiAuPd/C samples in which the metal nanoparticles were composed of random alloys, and the catalysts were applied to the dehydrogenation of formic acid.^77^ The catalysts were prepared by simultaneous reduction of all three metal precursors in aqueous solution in the presence of an active carbon support suspended within the solution. The Ni:Au:Pd molar ratio was 40:15:45. HRTEM images illustrated that the diameter of the nanoparticles was in the range of 16–35 nm (Figure 6). Furthermore, images from energy-dispersive X-ray (EDX) analysis (Figure 6) confirmed the randomly distributed and homogeneous structure of the three metals within the pseudospherical nanoparticles and the lack of a core@shell structure. Inductively coupled plasma atomic emission spectroscopy (ICP-AES) measurements confirmed that the overall metal loading was 9.91% and the Ni:Au:Pd ratio was 36:18:46.

**Figure 7:**
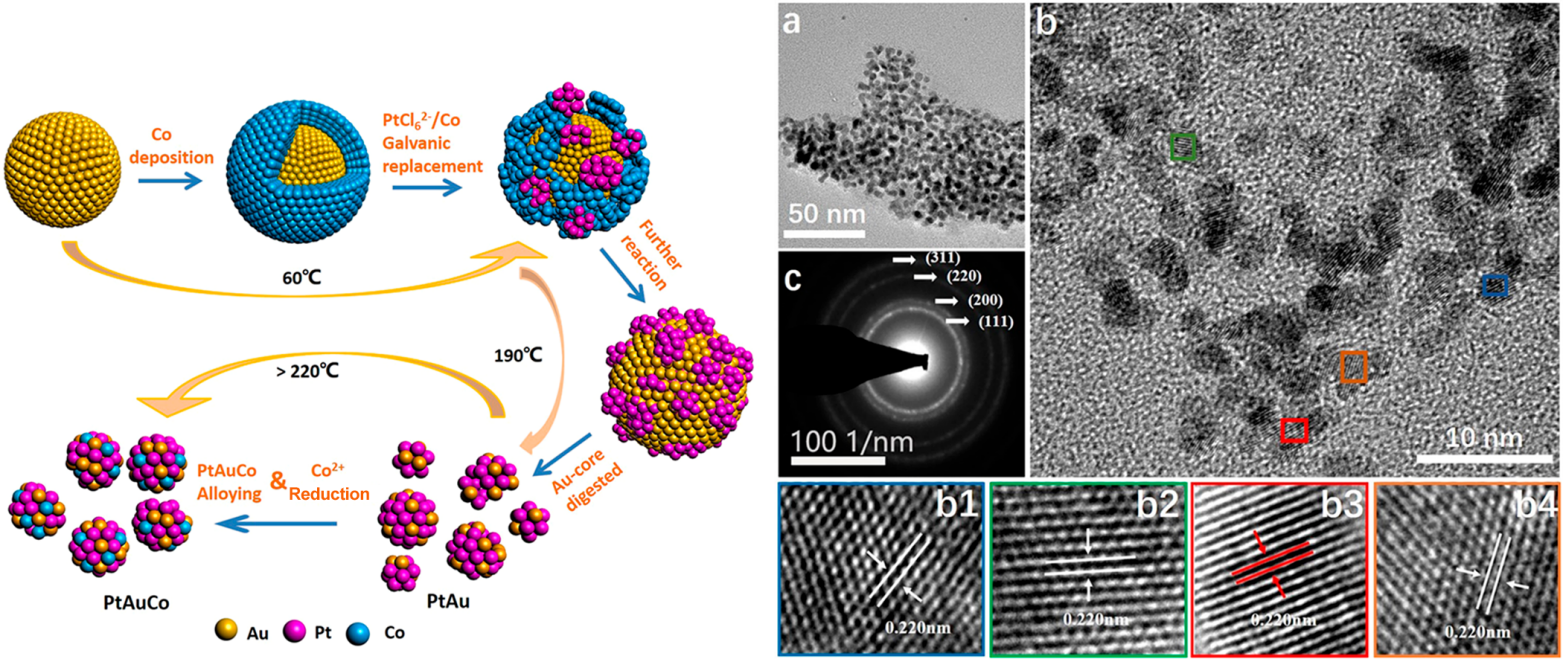
(left) Synthesis scheme to produce PtAuCo alloy catalysts. (right) Resultant morphology and structure of the unsupported Pt_76_Au_12_Co_12_ alloy TMNPs from electron microscopy analysis: (a, b) TEM images taken at different magnifications; (b1–b4) HRTEM images recorded from the different regions in (b); (c) selected-area electron diffraction (SAED) pattern. Adapted from ref (78). Copyright 2020 American Chemical Society.

Using a different design approach, Fu *et al.*(78) prepared unsupported, randomly alloyed PtAuCo TMNPs as catalysts for the dehydrogenation of ammonia borane. First, Au@Co core@shell bimetallic nanoparticles were prepared, and these were added to a solution of Pt precursor, digested with 1,2-hexadecanediol, and heated to 493 K. The approach led to TMNPs with a mean diameter of 4–5 nm in addition to smaller 1–2 nm particles, which are considerably smaller than most other reported TMNPs. The synthetic Pt:Au:Co molar ratio was 6.3:1:1. Analysis of EDX spectra confirmed that the metals were randomly distributed throughout the spherical particles. The design strategy is illustrated in Figure 7, along with electron microscopy images and a selected-area electron diffraction (SAED) pattern of such alloy catalysts.

Because of the additional degree of freedom when three metals are utilized, more variation is expected in the NP structures. Core@shell materials as M1@M2M3 were prepared by Kang *et al.* as shown in Figure 2).^79^*Via* a one-pot synthesis method, trimetallic Au@PdPt core–shell NPs were successfully synthesized with fine control over the nucleation and growth kinetics through manipulation of the reducing agents.^79^ In this example, an octahedral Au core and a highly crystalline PdPt alloy shell were prepared, which exhibited excellent catalytic activity and stability for the electrooxidation of methanol in acidic media. The enhanced catalytic activity was attributed to their optimized binding affinity for adsorbates due to the improved charge transfer between the core and the shell of the NPs. Computational modeling has been used extensively to understand how the geometric and chemical structure vary with AuPdPt trimetallic composition. Zhang *et al.*(80) observed charge transfer from Pd atoms to Au and Pt in Au-rich compositions, leaving the latter negatively charged; the Au present is therefore postulated to be able to activate molecular oxygen by electron donation.

There has been further extensive computational work using global optimization to investigate how the composition and nuclearity affect the chemical distribution and geometric structure for AuPdPt across a range of specific compositions and nuclearities, using both molecular mechanics and electronic structure simulation. There is a general consensus that Pt favors segregation to the interior of the nanoparticles while Au is strongly segregated to the surface for global optimizations under vacuum conditions, which is in line with thermodynamic expectations considering elemental cohesive and surface energies.^81−84^ In dynamical simulations for AuPdPt NPs encapsulated in carbon nanotubes (CNTs), Wei *et al.* showed that multishell structures are formed with Au accumulating near the core and Pt distributed near the nanotube wall, which highlights how thermodynamic stability can be influenced by the environment or support (as discussed in section 2.2).^85^

In several cases, AuPdPt and AgPdPt have been compared during simulation work, as both Au and Ag are in group 11. Wu *et al.* identified a preference for decahedral structures in both compositions but a greater preference for icosahedral structures for AuPdPt than for AgPdPt at a nuclearity of 75 atoms. With respect to composition, Ag was observed to alloy more than Au. Au prefers homogeneous bonding (*i.e.*, to other Au atoms),^86^ which was further confirmed by Du *et al.* for nuclearities up to 147 atoms.^87^

**Figure 8:**
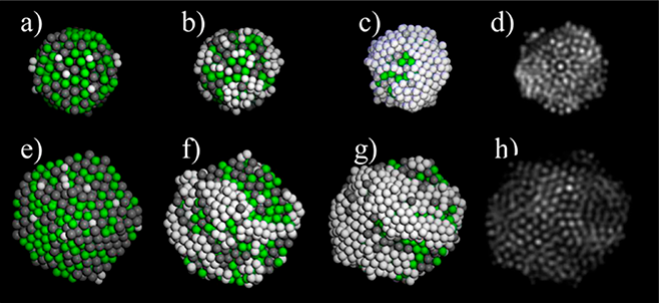
Monte Carlo simulations showed that Pt grown on AgPd nanoalloys forms 3D islands at the beginning of the deposition process. Ag_0.5_Pd_0.5_ icosahedra (top row) and decahedra (bottom row): (a, e) initial stage of Pt (light-gray spheres) nucleation on Ag_0.5_Pd_0.5_; formation of Pt deposits on AgPd at (b, f) Θ = 0.5 monolayer (ML) and (c, g) Θ = 1 ML; (d, h) STEM simulations of configurations at Θ = 1 ML. Adapted with permission from ref (89). Copyright 2013 Royal Society of Chemistry.

Further work on AgPdPt has confirmed observations that Pt tends to occupy a position at the core of these trimetallic NPs, with Ag at the surface (similar to Au).^88^ Khanal *et al.* produced an interesting study looking at the growth of AgPdPt nanoparticles using step-based synthesis, which is often overlooked in the search for thermodynamic global minima.^89^ Monte Carlo simulations showed that Pt grown on AgPd nanoalloys forms three-dimensional (3D) islands at the beginning of the deposition process (Figure 8). Akbarzadeh *et al.* also looked at the AgPd@Pt configuration with molecular dynamics simulations and identified a higher melting temperature when phase separation is realized in this core@shell manner. When the Pd concentration was increased and the Ag concentration reduced, the melting temperature rose, which is in line with elemental properties but also associated with reduced strain on the system when Pd atoms are at the core of the NP.^90^

**Figure 9:**
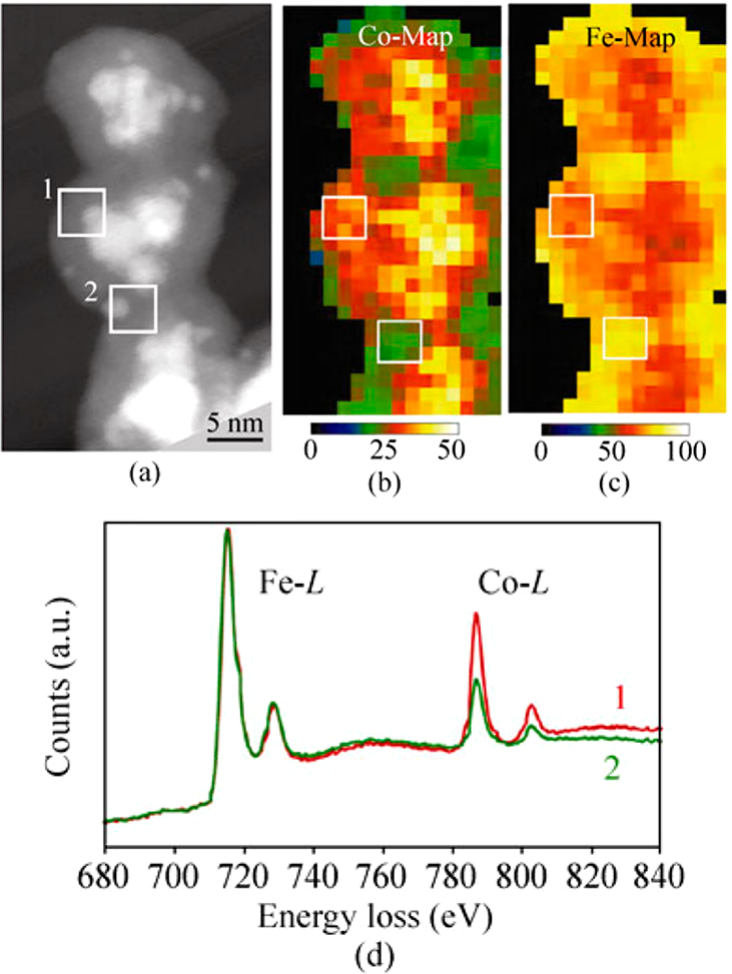
(a) HAADF-STEM image of AuCoFe triple-layer core@shell NPs, (b, c) EELS elemental maps for (b) Co and (c) Fe, and (d) EELS spectra at the areas indicated in (a). Adapted with permission from ref (91). Copyright 2011 Tsinghua University Press and Springer.

Considering further such layered NP structures, Au@Co@Fe triple-layer core@shell NPs (Au:Co:Fe molar ratio = 6:6:88) were synthesized *via* a one-step preparation method using poly(vinylpyrrolidinone) (PVP) as a stabilizing agent.^91^ As the reducing agent was exposed to the metal precursors at the same time, the resultant triple-layer core@shell structure is based on the reduction potentials of the metal cations as a measure of their ability to undergo reduction (*E*_Fe(III)/Fe(II)_^°^ = +0.77 V vs the standard hydrogen electrode (SHE); *E*_Fe(II)/Fe_^°^ = −0.44 V vs SHE; *E*_Co(II)/Co_^°^ = −0.28 V vs SHE; *E*_Au(III)/Au_^°^ = +0.93 V vs SHE). The Au core can be formed relatively quickly because of its higher reduction potential and can serve as an *in situ* seed for the catalytic reduction leading to the formation of the outer layers. This is an example of a successive reduction process for the formation of triple-layer core@shell trimetallic catalysts, as illustrated by HAADF-STEM imaging and electron energy loss spectroscopy (EELS) elemental maps revealing a Au core, a Co-rich interlayer, and an Fe-rich shell (Figure 9).

Combinations of group 11 elements are popular in TMNPs because of the aspiration to replace expensive Au with cheaper Ag and Cu without significantly changing the valence electronic structure, and these have been extensively investigated using computational methods. For example, utilizing Ag, Au, and Cu, Yildirim and Arslan^92^ showed that Ag and Au segregate to the surface of 38- and 55-atom NPs while Cu segregates to the core, in agreement with the higher surface energy of Cu. Segregation varies slightly for smaller systems (*N* < 10), where Cu and Ag take higher-coordination sites and Au the lower-coordination exposed sites.^93^ The preference of Au for surface sites has also been observed for CuAuPt alloys with nuclearities up to 147 atoms, with Pt and Cu preferring positions in the core; this observation is driven by the stronger cohesion energies and high surface energies of Pt and Cu.^94,95^ Taran considered how composition affects the melting of 55-atom Cu–Au–Pt trimetallic nanoalloys using molecular dynamics; Au was observed to be more mobile than Cu and Pt, which would favor Au diffusion to the surface of the aforementioned larger particles.^96^ For larger models that considered the specific structure of CuPt alloys on a Au core with molecular dynamics (MD) simulations, increasing concentrations of Pt led to greater stability, which is due to the greater cohesive energy of this species.^97,98^ Mattei *et al.*(99) considered AuPtPd systems, comparing MD and Metropolis Monte Carlo (MMC) simulations with laboratory experiments; the experiments showed Pd-rich shells and Au/Pt cores, and this observation was validated by modeling of the growth dynamics, with Pd structures forming quicker than Au/Pt and enveloping these particles during coalescence.

In some cases, subnano clusters have been considered, such as the four-atom AlTiNi clusters modeled by Koh *et al.*,^100^ where complex multiscale global optimization was applied. The interest in this system is motivated by the jellium behavior observed for small Al clusters; in this case, the stability of the clusters increases with the number of nickel atoms. Doping of 13-atom NiAg clusters with Cd and Cr was investigated by Datta using DFT calculations.^101^ The undoped NiAg clusters form a core@shell segregated system with smaller Ni in the core; doping was observed to enhance the mixing of the system, with Cd dopants being more effective than Cr. Taran *et al.* performed global optimization simulations on PtNi@Ag core@shell structures, which are of interest for electrocatalytic applications,^102^ and observed that, similar to Datta’s work, Ni atoms occupy core regions close to Pt atoms in the icosahedral structures; however, the core Pt atoms are under high stress, which is reduced by replacement with Ni atoms.

Overall, it is clear that the composition of TMNPs can give rise to morphological diversity and interesting structural features across a variety of sizes, which may then be exploited in catalytic processes. The broad research efforts in both synthetic and computational studies not only speak to this but also highlight the opportunity to develop this field further in novel ways.

### 2.2 Influence of the Support

Commonly, NP catalysts are synthesized with a support, which can improve their overall performance. Although unsupported colloidal trimetallic catalysts have shown promise,^103−106^ there are also many advantages of using a support. The advantages include improved dispersion of NPs, enhanced surface area, and increased stability against deactivation processes such as agglomeration.

However, it is important to consider that the support is not necessarily inert and may participate in the reaction. In the 1970s, the term “strong metal–support interaction” (SMSI) was introduced by Tauster to explain the unusual adsorption behavior of H_2_ over noble metal catalysts supported on TiO_2_.^107,108^ The metal–support interaction (MSI) is therefore a crucial consideration, as are the many influential factors on MSI,^109^ and it is desirable to understand these influences because tuning the MSI may facilitate optimization of catalytic properties through catalyst design.^110−114^

The number of catalyst supports available is large, and when selecting a support there are a number of factors one must consider, including mechanical and thermal stability, surface area, accessibility of active sites, porosity, and nanostructure. The support may also provide beneficial characteristics toward the reaction, such as defect sites, charge transfer ability, spillover, and additional functionality to the NPs.^109^ The development of a wide variety of novel supports has resulted in a huge diversification of supported heterogeneous catalysts, and a number of these novel supports have been employed in trimetallic catalysis.^115^ Graphene is a very promising option with regard to stability and is particularly favorable for electrocatalysis because of its unique charge transfer properties.^116^ Graphene is commonly in the form of few-layer graphene rather than the single-layer graphene to increase the stability,^117^ and graphene and graphene oxide (GO) have shown encouraging results as catalyst supports for a diverse selection of reactions.^118−123^ CNTs have incredible stability due to their nanostructure, which also allows exceptional distribution of NPs and small average particle sizes.^28,124−126^

There is currently limited information available for direct comparison of supports for trimetallic catalysts because of the recent development of the TMNP domain; however, reports have emerged that illustrate the importance of choosing the appropriate support and method of preparation when working with trimetallic catalysts, such as the use of different carbon support types for CO hydrogenation over alkali-promoted CoRhMo catalysts.^112,113^ These choices can drastically impact the overall catalytic performance.^124,125,127−132^ These aspects of TMNP catalyst design will be examined in greater detail in the following sections on catalyst preparation and testing.

## 3 Preparation of Trimetallic Catalysts

A variety of methods are used to synthesize NP catalysts, including impregnation, sol immobilization, coprecipitation, and microwave-assisted methods. Not only have these techniques been used to synthesize supported monometallic catalysts, but they have also provided stable and active multimetallic catalysts. The addition of a third metal can result in complications over the control of the NP composition but can offer greater tunability. Herein, we shall explore these methods to demonstrate how they can be successfully applied to the synthesis of heterogeneous TMNP catalysts.

### 3.1 Impregnation Preparations

Impregnation is one of the more traditional methods for the synthesis of heterogeneous catalysts. An excess of solution is used in wet impregnation, where a dissolved metal component is present. Wet impregnation is a relatively simple method to deposit metal NPs onto a support. Activation, through calcination or reduction in a high temperature furnace, is preceded by a drying step. Wet impregnation has been successfully applied to prepare various TMNPs as catalysts.^133,134^ Examples of supported TMNPs prepared *via* this technique include NiMoW supported on SBA-15^135^ and PdNiAg NPs supported on carbon.^27^ An analogue to this technique is incipient wetness impregnation (IWI), which uses a volume of metal precursors in solution equal to (or just below) the pore volume of the support, followed by a drying step; the catalyst is then typically activated by heat treatment under either a reductive, oxidative, or inert atmosphere as a final step.^136^ Other various TMNPs have been synthesized by impregnation: for example, a pore-filling coimpregnation method was applied by Nakaya *et al.* in preparing Pt_3_(Fe_0.75_M_0.25_)/SiO_2_, where M = Co, Ni, Cu, Zn, Ga, In, Sn, or Pb.^137^ Supported trimetallic AuPdPt alloys were synthesized by a conventional wet impregnation method by He *et al.*(133) Enhanced catalytic performances were observed in the solvent-free oxidation of benzyl alcohol over these AuPdPt catalysts, in part as a result of the reduction in selectivity for the byproduct toluene. However, the catalytic activity was much lower than over TMNPs prepared by alternative methods. The lower activity observed was thought to be due to the significant increase in mean particle diameter when such an impregnation technique was used compared with the colloid-derived material.

Generally, impregnation methods are known to produce a greater particle size and particle size distribution. Lopez-Sanchez *et al.*(138) reported the particle size distribution of supported AuPd metal NPs prepared by both sol immobilization and an impregnation route. The synthesized particles ranged in diameter between 4 and 7 nm in the catalyst prepared by sol immobilization, and a much wider particle diameter distribution of 2–14 nm was observed in the latter route. Bahruji *et al.* also observed that Pd/ZnO catalysts synthesized by impregnation produced larger diameters of metal NPs after heating in H_2_ at 673 K (ca. 8.8 nm) in comparison with those prepared by sol immobilization (ca. 4.9 nm).^139^

### 3.2 Colloidal Preparations

In an attempt to eliminate the larger particle sizes observed with some impregnation methods (*i.e.*, those over 8 nm in diameter), facile chemical reduction preparation methods are often utilized, with various terminology including, but not limited to, coreduction, sol immobilization, and one-pot synthesis. Although coreduction methods demonstrate greater control of particle size and particle size distribution in multimetallic systems, compositional fluctuations with respect to neighboring particles are often observed, which needs further investigation.^133,138^ As trimetallic catalysts attract greater interest, colloidal preparation methods have been successfully reported and applied to a range of catalytic reactions such as glycerol oxidation,^25^ methanol oxidation,^118^ and hydrolytic dehydrogenation of ammonia borane.^91^ Colloidal nanoalloys can be prepared by adding an excess of stabilizing agent and reducing agent to the metal precursor(s). These stabilizing agents are often donor ligands, polymers, and surfactants, such as PVP, poly(vinyl alcohol) (PVA), and CTAC, which also control the growth of the initially formed nanoclusters.^139−142^ Protective agents are often deemed as essential through either electrostatic stabilization or steric stabilization of the colloids, as demonstrated by Nam *et al.* in the shape-controlled synthesis of intermetallic PdSn nanocrystals.^143^

Conversely, studies have been conducted to show successful activity and stability under surfactant-free conditions using a facile chemical reduction preparation method. Wang *et al.* demonstrated the synthesis of a well-dispersed random alloyed trimetallic catalyst composed of NiAuPd NPs deposited on carbon black in the absence of a stabilizing agent.^77^ Yurderi *et al.* also synthesized a stabilizer free PdNiAg/C catalyst that showed high activity for the dehydrogenation of formic acid, and maintained activity after the forth reusability test, without a stabilizing agent.^27^ These studies successfully demonstrated the synthesis of stable TMNPs as catalysts that resist agglomeration under surfactant-free conditions; however, the use of protecting agents is more important for unsupported colloids, as these NPs are unstable with respect to agglomeration.^144^

Often, a major drawback of the use of TMNPs in catalysis is the complexity that potentially arises from combining three metal precursors, all with different reduction potentials. The multielement composition makes the growth kinetics and structural characteristics complex and difficult to control, which will affect the overall catalytic performance. Kang *et al.*(79) developed a facile one-pot aqueous synthesis method to obtain AuPdPt TMNPs with a controlled nanostructure by using simultaneous reduction of all three metal precursors in a 1:1:1 molar ratio with separate reducing agents (hydrazine and ascorbic acid) in the presence of CTAC. Their method resulted in an octahedral Au core with a single-crystalline shell of dendritic Pd–Pt alloy.

In order to study the formation mechanism, coreduction of the Au and Pd precursors with CTAC and no additional reagents was performed and yielded core@shell Au@Pd {111}-faceted octahedral nanoparticles with an average edge length of 61.0 ± 1.3 nm.^79^ When the synthesis was repeated with the addition of the Pt precursor, spherical Au@PdPt nanoparticles were formed with a polycrystalline shell having an average edge length of 43.3 ± 5.1 nm. Thus, in this case the addition of Pt influenced the metal and surfactant interactions. When just hydrazine was added to the mixture of metal precursors and CTAC, polyhedral Au monometallic nanoparticles formed in a variety of shapes, and Pd or Pt were not visible *via* EDX analysis of the final product; when only ascorbic acid was added, dendritic Pd–Pt nanoparticles were formed. The observations confirmed that both ascorbic acid and hydrazine were essential to produce the well-defined shape of the octahedral core. Further investigations with UV–vis spectroscopy revealed a peak at 285 nm, which was attributed to ligand-to-metal charge transfer (LMCT) between the Pd precursor and CTAC. The Pd–CTAC complex is essential in promoting the structure direction of the nanoparticles. If CTAC is substituted with other surfactants, the octahedral shape of the Au core is not obtained. Although the method outlined clearly works very well for Au@PdPt nanoparticles, it does not appear to be applicable to other trimetallic systems, as altering the type or amount of reducing agents, the surfactant, or the molar ratio of metal precursors has been shown to affect the composition, structure, and morphology of the nanoparticles, which has a negative effect on the catalytic performance.^79^

The successful generation of the Au_oct_@PdPt core@shell NPs with well-defined octahedral Au core and a dendritic PdPt shell was achieved exclusively when both ascorbic acid and hydrazine were employed in the synthesis as reducing agents. The Au_oct_@PdPt particles had an average edge length of 61.0 ± 1.3 nm and shell thickness of 13.6 ± 1.6 nm. When ascorbic acid was solely used as a reductant in the synthesis, dendritic PdPt alloy NPs were produced instead of ternary AuPdPt NPs. Spherical Au cores were formed without the presence of ascorbic acid and hydrazine, indicating the influence of the reducing agents. EDX elemental mapping confirmed the core–shell structure, and the Pd–Pt shell was further observed *via* XRD analysis. ICP-AES estimated the Au:Pd:Pt atomic ratio to be 55:25:20.^79^ The effect of the reaction time was investigated by studying the NPs after a range of reaction times. After 1 min, octahedral NPs formed, and after 10 min small dendrites were observed on the NP surface. These protrusions were more abundant after 20 min. Further increasing the reaction time continued to increase the growth of the branches on the NP surface, and after 150 min the well-defined Au_oct_@PdPt core–shell TMNPs were formed.^79^

The effect of the reducing agent on the nucleation and growth kinetics was also investigated. When only CTAC was used in combination with the metal precursors, spherically shaped Au@PdPt NPs were formed with an average particle diameter of 43.3 ± 5.1 nm. The Au core was spherical in shape with an average diameter of 27.8 ± 3.9 nm, and the Pd–Pt shell was dendritic in structure with an average thickness of 7.6 ± 1.3 nm. The Pd–Pt shell was observed to be polycrystalline by SAED, suggesting that the shape of the Au core may influence the crystallinity of the shell. The Au_oct_@PdPt NPs could be produced only when both ascorbic acid and hydrazine were used simultaneously as reducing agents. When only ascorbic acid was used, bimetallic dendritic Pd–Pt NPs were produced. EDX showed Pd–Pt alloying with no Au present, even though Au has the highest reduction potential of the three metals; the Pd:Pt atomic ratio was 65:35 as determined by ICP-AES. When hydrazine was used exclusively as the reducing agent, polyhedral monometallic Au NPs were produced in a variety of shapes, and EDX showed that no Pd or Pt was present. The formation mechanism of the octahedral Au core was found to be influenced also by the metal precursors present, as when just HAuCl_4_ and K_2_PdCl_4_ were reduced by hydrazine, homogeneous octahedral Au NPs were synthesized, but the polyhedral Au NPs were produced when the K_2_PtCl_6_ precursor was also included. UV–vis adsorption spectra showed evidence of a peak corresponding to LMCT between the Pd precursor and CTAC, indicating that this interaction is a crucial component for producing the well-defined octahedral shape. Replacing CTAC with a different surfactant that does not contain ammonium resulted in an absence of the octahedral shape. When Pt is present, it preferentially binds to hydrazine ahead of Pd because of the stronger binding affinity. Ascorbic acid is therefore additionally required to direct the formation of the Pd–CTAC complex. The equimolar combination of the metal precursors was also crucial to form the well-defined Au_oct_@PdPt NPs; when Au was in excess, the octahedral shape of the Au cores could not be directed. Similarly, when Pt was in excess, its inhibiting effect on Pd–CTAC complex formation could not be eliminated by ascorbic acid. Au@PdPt NPs did form when Pd was in excess, but the shapes of the cores were inhomogeneous. Using the appropriate amounts of both reducing agents was therefore crucial to both the morphology and the material yield.^79^

Other trimetallic systems that have been synthesized by a one-pot method include Cu/Au/Pt,^106,145^ PtAuRu,^146^ Ag@PdAu,^147^ and (Pd,Co)@Pt.^148^ These examples appear to have been prepared following a significant amount of trial and error to identify the optimum conditions for reduction, and the “one-pot method” does not appear to be universally applicable.

A technique that is complementary to colloidal methods is the use of polyols (*e.g.*, the diol ethylene glycol) to synthesize metal NPs through chemical reduction of metal salts. These liquid organic compounds can act as both the solvent and the reducing agent. At elevated temperatures, ethylene glycol can reduce metal cations to their metallic state and therefore encourage the growth of nanostructures. Advantages of using polyols include facile formation of crystallized materials and the ability to coordinate to metal particle surfaces to minimize aggregation. Such polyol compounds often have high viscosities, favoring diffusion-controlled particle growth that results in controlled structures and morphologies.^149^ The polyol method has been successfully applied to prepare TMNPs as catalysts, such as PdRuNi supported on graphene oxide for the dehydrocoupling of dimethylamine borane^121^ and PdCoPt/C for electrocatalytic oxygen reduction reactions.^148^ Adaptations of the polyol method have been used to synthesize (Pd,Co)@Pt NPs supported on Ketjenblack carbon by sonochemical reactions of Pt(acac)_2_, Pd(acac)_2_, and Co(acac)_2_ in ethylene glycol; with an elemental composition of Pd_50_Co_20_Pt_30_, the resultant trimetallic catalysts showed superior activity and stability, due to synergistic effects, for electrocatalytic oxygen reduction reactions compared with commercial Pt/C.^148^

Microwave (MW)-assisted polyol reduction methods have also been reported for the successful preparation of TMNPs, such as PdRuNi/GO,^121^ used as a catalyst for dehydrocoupling of dimethylamine borane, and PtRuFe supported on N-doped graphene, used in methanol oxidation and oxygen reduction reactions.^150^ Further examples of trimetallic catalyst preparation by MW irradiation methods have recently emerged. Typically, MW-assisted material synthesis is a highly controllable and rapid method to synthesize nanoparticles. The main benefit of MW techniques is that the internal heating provided by MW irradiation is much faster than that in conventional chemical reactions heated in an oil bath or oven. Furthermore, modern microwave reactors provide a high level of control over reaction conditions such as temperature, pressure, and heating rate, creating a very stable reaction environment. Depending on the method, MW irradiation may be applied to all of the metal precursors at once,^121,151^ or one metal precursor may be reduced to form a core NP first, after which the remaining precursors are added before MW synthesis is carried out.^152−154^

### 3.3 Obtaining Multimetallic Nanoparticles *via* Galvanic Replacement

Galvanic replacement or displacement has emerged as one of the more commonly used methods for synthesizing both supported and unsupported multimetallic nanoparticles.^155−162^ The method can be used to apply a third metal to a bimetallic NP system, resulting in core@shell or pseudo-core@shell structures as well as “nanorings”.^163^

The galvanic replacement reaction (GRR) occurs between the zero-valent surface metal atoms of the nanoparticle and metal precursors in solution. It is driven by the difference in reduction potentials between the surface metal atoms and the cations in solution, resulting in oxidation of the surface atom *via* reduction of the cation to its neutral state. As such, the process is sensitive to the chemistry of the metallic species at the surface and the cation in solution.^164^ Galvanic replacement demonstrates a flexible design approach in which the morphology and electronic composition of the active sites of the TMNP can be finely and independently tuned.^165−168^Equation 1 summarizes the overall reaction occurring at the surface sites:
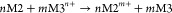
1The process effectively replaces *n* surface atoms of metal M2 with *m* atoms of metal M3, resulting in an M1M2@M1M3 pseudo-core@shell structure. The reaction is highly selective, as only one element on the surface is replaced. The process is also strictly a surface modification, as the third metal does not enter the interior of the nanoparticle, and therefore, the inherent morphology of the nanoparticle is not changed.^165^ Since the replacement reaction is a gradual process, the degree of replacement can be controlled by varying the reaction time, hence affording control over the stoichiometry of metal M3.^169^

Sahoo *et al.*(170) used the sacrificial oxidation of cobalt nanoparticles that were initially prepared from a CoCl_2_ precursor, reduced with NaBH_4_, and capped with sodium citrate to prepare TMNP catalysts. Initially, the reaction takes place under N_2_ to prevent oxidation of Co. After reduction is complete, a deaerated solution of HAuCl_4_ is transferred to the Co nanoparticle solution in the absence of oxygen. A solution containing PVP is then added as a stabilizing agent, and the sacrificial oxidation of Co^0^ to Co^2+^ spontaneously reduces the Au^3+^ ions to Au^0^ as Au nanoparticles are formed and Co^2+^ re-enters the solution. NaBH_4_ is then added to the solution of Au nanoparticles and Co^2+^ ions, which rereduces Co^2+^ ions to deposit a layer of Co^0^ around the Au nanoparticles. When this reduction is complete, a K_2_PdCl_4_ precursor solution is added to the mixture and stirred for 6 h at room temperature and then for a further 2 h at 323 K. Again, the precious metal, in this case Pd, is reduced from Pd^2+^ to Pd^0^ while Co^0^ is oxidized to Co^2+^. This solution is deaerated, and the process is then repeated using the RuCl_3_·H_2_O precursor to obtain triple core@shell Au@Pd@Ru nanoparticles.^170^ The disadvantage of the galvanic displacement method is that the final average nanoparticle diameter is large, at 110 nm, which means that a lot of the metal atoms are in the bulk and therefore unavailable for catalytic processes on the surface of the nanoparticle. Nevertheless, the Au@Pd@Ru nanoparticles were evaluated as catalysts for dye degradation and wastewater treatment and were promising compared with mono- and bimetallic analogues.

**Figure 10:**
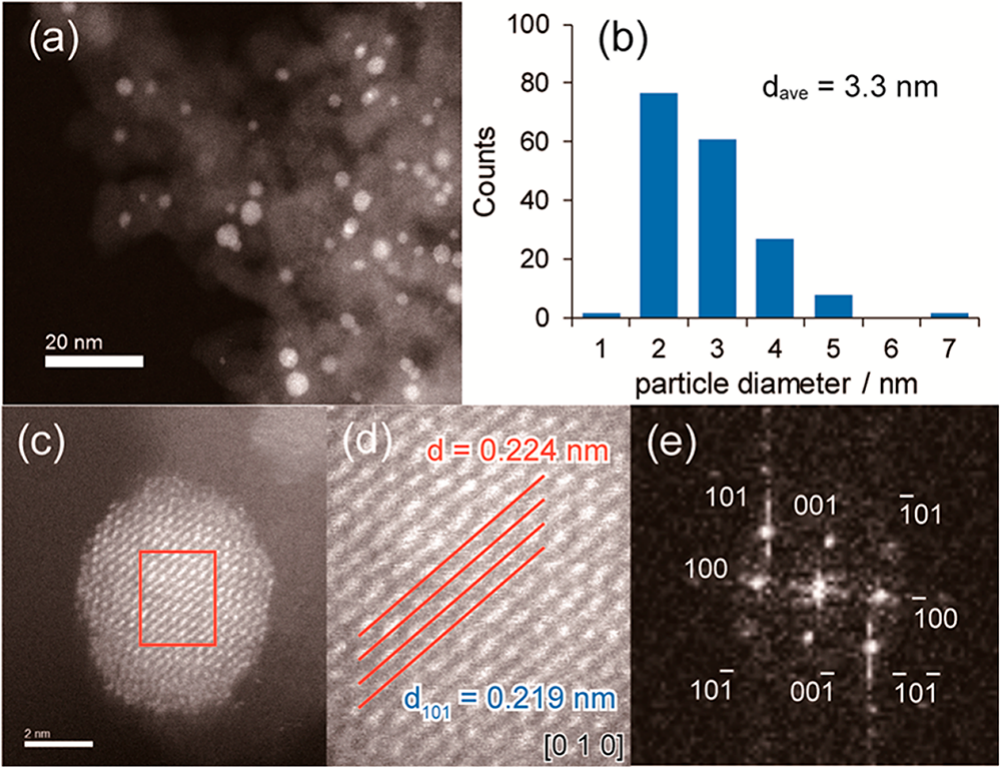
(a) TEM image of PdZn@Pb_0.25_ TMNPs. (b) Size distribution of the TMNPs. (c) HRTEM image of a single nanoparticle. (d) Further magnification of the region denoted by the red square in (c), highlighting the lattice fringes on the (010) plane. (e) Fast Fourier transform of the single nanoparticle. Adapted from ref (165). Copyright 2019 American Chemical Society.

Miyazaki *et al.* reported the use of GRR to add a third metal (M) to PdZn/SiO_2_ such that Pd:Zn:M = 1:1:0.25.^165^ First, PdZn/SiO_2_ was synthesized by pore-filling coimpregnation of aqueous Pd(NO_3_)_2_ and Zn(NO_3_)_2_·6H_2_O on dried silica gel (specific surface area of 470 m^2^ g^–1^) to obtain a 3% loading of PdZn. The resulting product was reduced and stored under argon prior to the introduction of an aqueous solution of the third metal precursor. HRTEM images indicated that the diameters of the PdZn/SiO_2_ BMNPs were between 2 and 6 nm with a mean of *ca.* 3 nm. HRTEM images of a single PdZn nanoparticle, with the CsCl-type crystal structure, showed lattice fringes with a spacing of 0.217 nm. This spacing corresponds to the PdZn(010) planes, calculated to be 0.219 nm, and is indicative of an intermetallic bimetallic alloy of PdZn. Following addition of Pb *via* galvanic replacement, TEM images of PdZn@Pb_0.25_ showed a mean diameter of 3.3 nm (Figure 10). Minimal changes in the morphology of the nanoparticle were reported following galvanic replacement, which is as expected. The spacing of the (010) plane lattice fringes was reported to be 0.224 nm, similar to that of the PdZn(010) planes previously calculated. A fast Fourier transform of the HRTEM image confirmed the *P*4/*mmm* space group oriented along the (010) plane, comparable to analysis of a PdZn single crystal. EDX mapping of the HRTEM image confirmed the pseudo-core@shell structure, wherein Pd was homogeneously dispersed throughout the nanoparticle, Zn was found exclusively within the core, and Pb was found exclusively in the shell (M1M2@M1M3 structure). In this galvanic replacement reaction, Zn in the surface is replaced with Pb, whereas Pd remains in place. This is the case because Zn has a lower oxidation potential than Pd and is capable of reducing Pb^2+^ to Pb, as well as many other late transition and p-block metals. (Zn^2+^ + 2e^–^ → Zn, −0.76 V vs NHE). This catalyst composition was compared to the Lindlar catalyst (Pd–Pb/CaCO_3_) in the hydrogenation of phenylacetylene to styrene, with the galvanic replacement prepared catalyst, comprised of PdZn@Pb_0.25_, exhibiting a high activity and importantly an enhanced R_1_/R_2_ ratio, indicating a reduced overhydrogenation rate of styrene.

Galvanic replacement has been extensively investigated by Skrabalak and co-workers as a way to access multicomponent materials and catalysts, including heterostructures that may offer unique opportunities in catalysis. As an extension or development of the work discussed above, the formation of heterostructures from Pd nanocubes or AgPd heterodimers has been reported,^171^ along with structures from intermetallic PdCu nanoparticles.^172^ These architecturally complex, multicomponent materials possess unique structural motifs and junctions, for example in the formation of a PdCu–Au Janus particle^172^ However, when Pt was added to the same intermetallic PdCu nanoparticles, no Janus particles were formed. The difference was ascribed to the surface mobility of Au versus Pt, as Au has a higher surface mobility and resulted in the segregated domains.

The use of TMNPs or nanostructures as templates for quaternary or quintenary NPs *via* GRR appears to be rare. However, the preparation of tetrametallic AgAuPtPd nanotubes from multidomain AgAuPd nanotubes has been investigated.^173^ This four-component structure was formed *via* a sequential GRR starting with Ag nanowires, to which Au was added with CTAB to form multidomain nanotubes. Pd (with CTAB) was added to this structure, and finally, Pt (with CTAB) was added to form the nanotubes. The material was imaged with HAADF-STEM, which revealed that the metals were well-mixed and were effective at catalytic reduction of 4-nitrophenol with NaBH_4_.

### 3.4 Preparations of Specialized Trimetallic Nanostructures

Novel approaches toward trimetallic systems have been reported in which defect sites on a bimetallic system have acted as seeds for controlled addition of a third metal.^26^ Such a method has paved the way for design of trimetallic catalysts at the atomic level. One example used Pt_3_Ni nanocrystals with a concave octahedral morphology containing many surface defect sites, and deposition of Ni was carried out to form Pt_3_Ni@Ni, which was of regular octahedral shape. Overall, several trimetallic catalysts of the form Pt_3_Ni@M_8_ (M = Au, Ag, Cu, Rh) were prepared. As the high-energy defect sites are less thermodynamically favored, nanoparticles often possess relatively few natural defect sites; this issue can be overcome for the outlined purposes by using chemical etching to introduce many additional defect sites.^174^ In the example reported by Wu *et al.*, the recovered nanocrystals consisted of many surface sites of segregated Pt, with step edges and step terraces in contrast to the eight (111) facets expected in a regular octahedron.^26^ Proof-of-concept experiments with a PVP (*M*_w_ = 8000)-capped Ni shell confirmed that growth of the shell was seeded by these defect sites, reforming a regular octahedron with eight Ni(111) facets. Finally, catalysts were prepared using other metals (M) to form Pt_3_Ni@M_*x*_ core@shell TMNPs. The catalysts containing 0.5 atom % Pt were evaluated for the Suzuki–Miyaura reaction and reduction of nitrobenzene using formic acid as a hydrogen source. The stoichiometry of M was controlled by varying the concentration of M precursor and was set at *x* = 0.5, 2, or 8. Pt_3_Ni@M_8_ represented a perfect octahedron, whereas a low M stoichiometry such as Pt_3_Ni@M_0.5_ was still of concave morphology. XPS data confirmed the addition of the third metal on the defect-rich concave region of the nanoparticles. TEM images confirmed particle diameters of 10–13 nm for all of the catalysts that were synthesized. Catalysts containing Au (0.5 atom %) as the third metal had significantly enhanced reaction rates compared with a commercial example and for the reduction of nitrobenzene, where with other metals (Cu, Rh, and Ag) the rate was lower.

**Figure 11:**
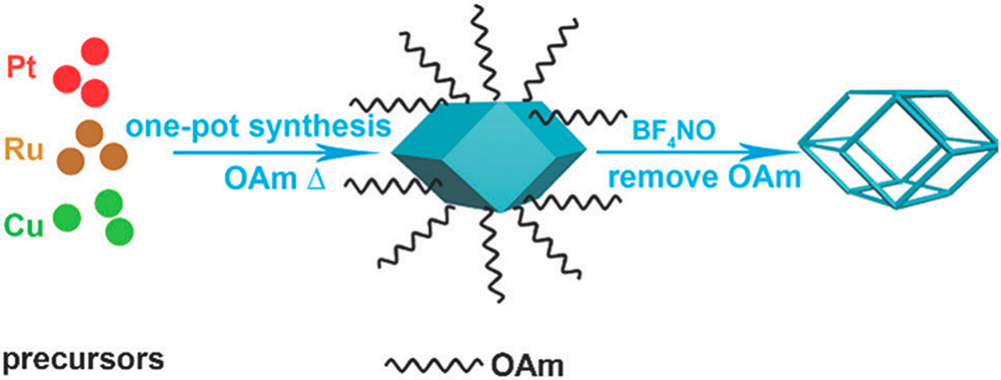
Schematic displaying the formation of PtRuCu nanoframes *via* formation of the nanoparticle and subsequent etching to form the hollow frame. Adapted with permission from ref (175). Copyright 2020 Royal Society of Chemistry.

Etching methods have also been used for the synthesis of trimetallic nanoframes, as reported by Yin *et al.*(175) First, trimetallic PtRuCu nanoparticles were synthesized *via* hydrothermal methods using K_2_PtCl_6_, CuCl_2_·2H_2_O, and RuCl_3_·*x*H_2_O with oleic acid and oleylamine capping (Figure 11). The mixture was heated to 473 K with stirring and then heated in a Teflon-lined stainless-steel autoclave. The PtRuCu TMNPs were dispersed in a 1:1 mixture of cyclohexane and DMF. The etching agent, BF_4_NO, was added with stirring. The mixture was centrifuged and washed with DMF. The nanoframes were dispersed in 3:1 DMF/acetone, and finally, carbon black (Vulcan XC-72) was dispersed into the mixture followed by sonication. The catalysts were activated in a N_2_-saturated HClO_4_ aqueous solution.

The resulting frames had a rhombic dodecahedral shape and were 40–80 nm in diameter. The three metals within the frame were reported to be alloyed and evenly distributed, and the Pt:Ru:Cu ratio was 40.7:0.3:59. The low Ru content was understood to be due to a mismatch of the Ru lattice structure with those of Cu and Pt; Ru adopts a hexagonal closed-packed (HCP) structure, whereas Cu and Pt adopt FCC structures.^176^

Another specialized nanostructure, the one-dimensional nanowire, was reported by Zhu *et al.*(177) and was synthesized with a sacrificial template composed of a tellurium nanowire. These materials were evaluated as electrocatalysts for ethanol electrooxidation. The Pd–Pt–Au nanowire was prepared by hydrothermal synthesis from an aqueous suspension of Te nanowires that was charged with H_2_PdCl_4_, H_2_PtCl_6_, and HAuCl_4_. Bimetallic samples, PdPt and PdAu, were also prepared. Trimetallic formulations included Pd:Pt:Au molar ratios of 5:5:1 (resulting in an atomic formulation of Au_18_Pt_6_Pd_76_), 4:16:1 (resulting in Au_17_Pt_24_Pd_59_), and 2:8:1 (resulting in Au_34_Pt_18_Pd_48_). The compositional differences observed in the final materials were attributed to the galvanic replacement reaction rates (HAuCl_4_ > H_2_PdCl_4_ > H_2_PtCl_6_). The nanowires were shown by EDX mapping to contain an evenly distributed and fully alloyed configuration, with no remaining Te apparent. The detailed mechanism of formation of the nanowires is still currently under investigation; however, in comparison with the commercial E-TEK Pd/C electrocatalyst, the Au_17_Pt_24_Pd_59_ nanowires were shown to have greater activity for ethanol electrooxidation in alkaline media.

Hydrothermal synthesis is another important method that has been used for the preparation of TMNPs. The technique involves heating a solution of dissolved metal precursors in an autoclave under autogenous pressure and can be an effective method to produce highly ordered structures in a facile manner. Examples of trimetallic nanoparticles prepared this way include PdNiAl NPs,^178^ NiFeCo NPs,^179^ PtAgCu@PtCu core@shell concave nanooctahedrons as catalysts for formic acid oxidation,^180^ (111)-terminated PtPdRh nanotruncated octahedrons for conversion of ethanol to CO_2_,^181^ and CoNiCu hydroxyphosphate nanosheets as electrocatalysts for the oxygen evolution reaction.^182^

An interesting approach to the formation of highly ordered arrays of multimetal nanoparticles was developed by Mirkin and co-workers through a tip-directed deposition technique.^183,184^ Here a metal or multimetallic precursor solution containing a block copolymer could be deposited on a suitable substrate such as Si *via* a technique termed scanning probe block copolymer lithography.^185^ This technique results in a high degree of deposition control, with particles spaced at regular intervals, and nanoparticles with a diameter of *ca.* 10 nm are formed following annealing at 500 °C. Formulations of nanoparticles included AuPd, PtNi, PdNi, PtCo, PdCo, and CoNi BMNPs and also AuAgPd TMNPs. These arrays were evaluated as catalysts for the conversion of 4-nitrophenol to 4-aminophenol with NaBH_4_. As an extension of this technique, the formation of quaternary and quinary nanoparticles that can be prepared as heterostructured nanoparticles was subsequently reported.^184^ The diversity of preparation techniques available to synthesize TMNPs as NPs deposited on a surface or as trimetallic nanoframes highlights the degree of control available to tailor materials to potential catalytic processes. In the next section we evaluate the catalytic applications of many of the materials discussed in this section and explore the benefits of TMNPs in important processes.

## 4 Catalytic Applications

There are several examples of the uses of trimetallic nanoparticles in catalysis, including oxidations, reductions, hydrogenations, decomposition reactions, and electrochemical reactions. The following section will include applications of specific compositions that have received particular attention in the literature. The majority of examples in the literature are for transition metals in groups 8–11. The exact role played by the addition of a third metal in their catalytic performance can be complicated, but it is apparent that TMNP-based catalysts can exhibit increased catalytic activity compared with their mono- and bimetallic counterparts. Here we explore the potential causes of this enhancement with different compositions of TMNPs. These enhancements often appear through side-reaction suppression *via* dilution of an active surface or subtle alteration of the active site through sterics or an electronic change of an active metal center upon addition of third metal.

### 4.1 Hydrogenation

Hydrogenation encompasses a wide variety of reactions that involve reduction of a molecule *via* the addition of hydrogen.^186^ Without a catalyst, hydrogenation would require high temperatures and pressures, so an appropriate catalyst is essential to ensure appropriate selectivity and feasible reaction conditions. Common examples of heterogeneous catalysts used in hydrogenation include ruthenium,^187,188^ Raney nickel,^189^ and the palladium-based Lindlar’s catalyst.^190^ More recently, a number of trimetallic examples have been reported that can further address the issues related to selectivity, and a selection are discussed in further detail in this section.

#### 4.1.1 Hydrogenation of CO to Higher Alcohols

Mixtures of higher alcohols have the potential to be used to blend with gasoline to improve its octane number and reduce the output of harmful emissions. Although these higher alcohols can be obtained from biomass-derived synthesis gas (syngas), the product is typically contaminated with H_2_S, which is a strong poison for many catalysts. Hence, two essential properties of catalysts for the synthesis of higher alcohols from biomass-derived syngas are (i) high activity for the water gas shift (WGS) reaction and (ii) superior sulfur resistance. MoS_2_ catalysts fulfill these criteria but have low selectivity for higher alcohols, instead producing methanol, hydrocarbons, and CO_2_. The addition of Rh shifts the activity toward oxygenates, and further addition of Co increases C_1_ to C_2_ homologation, encouraging the production of ethanol as a dominant product. Table 1 summarizes the influence of the support on the activities of TMNP catalyst examples discussed here, when applied to this reaction.

**Table 1:** Summary of a Selection of TMNP Catalysts Applied to CO Hydrogenation to Higher Alcohols

catalyst	catalyst type	reaction conditions	% CO conversion (selectivity for higher alcohols)	ref
Rh–Mo–K/MWCNT	alkali-promoted BMNP	*T* = 593 K; *p* = 8.3 MPa; GHSV^a^ = 3.6 m^3^ (STP)/(h kg cat) over 24 h; H_2_/CO molar ratio = 1	40.1 (24.6)	(124)
4.5 wt % Co–Rh–Mo–K/MWCNT	alkali-promoted TMNP		45.2 (31.4)	
6.0 wt % Co–Rh–Mo–K/MWCNT			48.9 (27.8)	
4.5 wt % Co–Rh–Mo–K/AC			31.2 (18.8)	
6.0 wt % Co–Rh–Mo–K/AC			35.3 (15.9)	
4.5 wt % Co–Rh–Mo–K/AC-Darco (microporous)	alkali-promoted TMNP	*T* = 603 K; *p* = 8.3 MPa; GHSV^a^ = 3.6 m^3^ (STP)/(h kg cat) over 48 h; H_2_/CO molar ratio = 2	35.6 (11.7)	(125)
4.5 wt % Co–Rh–Mo–K/AC-RX_3_ Extra (microporous)			39.6 (12.1)	
4.5 wt % Co–Rh–Mo–K/AC-Fluid Coke (mesoporous)			41.8 (12.7)	
4.5 wt % Co–Rh–Mo–K/AC-CGP Super (mesoporous)			44.5 (13.1)	
4.5 wt % Co–Rh–Mo–K/MWCNTs			52.4 (16.8)	

a Gas hourly space velocity.

The activities of alkali-promoted trimetallic Co–Rh–Mo sulfide catalysts for higher alcohol synthesis (C_2_ and higher) from syngas were assessed.^124^ When used as a support, activated carbon (AC) does not favor hydrocarbon formation and was reported to be resistant to changes in pH, high temperatures, and pressures. However, AC still suffers from coking, as it has micropores (<2 nm pore diameter) that are easily blocked, leading to transport limitations. Multiwalled carbon nanotubes (MWCNTs) have properties similar to those of AC, albeit with a meso/macroporous structure that circumvents the limitations of transport and metal dispersion. To synthesize the MWCNT-supported catalysts, the supports were pretreated with HNO_3_, and then metals were added using an incipient wetness impregnation method through the addition of first a K-promoter (9 wt %) followed by the metal precursors. A range of reaction conditions were tested, with higher temperatures and pressures leading to increased CO conversion and hydrocarbon formation rate, decreasing methanol selectivity, and increasing higher alcohol selectivity (593 K and 8.28 MPa were optimal).

Bimetallic Rh–Mo catalysts were also compared for higher alcohol synthesis (C_2_ and higher) from syngas, but the CO uptake increased when Co was added, up to 4.5 wt %, to form the trimetallic catalyst.^124^ The Co–Mo–S phase is considered to be the active site for higher alcohol synthesis, and the selectivity for higher alcohols over the 4.5 wt % Co–Rh–Mo–K/MWCNT catalyst was 31.4 wt %, compared with 24.6 wt % over Rh–Mo–K/MWCNT. When the Co content was increased above 4.5 wt %, an increase in large Co_9_S_8_ particles was observed that decreased the surface area and hence the metal dispersion of the Co–Mo–S phase. The selectivity for higher alcohols over catalysts containing 6 wt % Co supported on either AC or MWCNTs was subsequently reduced. Further analysis of the catalysts by temperature-programmed reduction (TPR) revealed that the addition of Co caused the reduction temperature to decrease, showing that addition of Co enhances the reducibility of the other metal species present at lower temperatures, and as the Co content was increased, this effect intensified. The highest CO conversion (48.9%) was observed over the 6 wt % Co–Rh–Mo-K/MWCNT catalyst.^124^ The catalyst with the lower Co content (4.5 wt % Co–Rh–Mo–K/MWCNT) showed a lower CO conversion (45.2%). The results imply that Co addition increases the number of active sites, as an improved CO conversion was observed, but when the Co concentration is increased beyond a certain level, the methanation activity increases as large Co_9_S_8_ sites form.

In a later study, the same group assessed the influence of the porosity of the carbon support on the performance of the alkali-modified trimetallic Co–Rh–Mo catalysts for CO hydrogenation to higher alcohols, testing four different commercial AC supports.^125^ Two of the AC supports had microporous characteristics (AC-Darco and AC-RX_3_ Extra), while the remaining two AC supports were mesoporous (AC-Fluid Coke and AC-CGP Super). The supports were compared to commercial MWCNTs, which are also mesoporous. In reflection of the group’s previous work, a 4.5 wt % Co–Rh–Mo–K catalyst was studied on each support.^124^ TEM studies showed that the TMNPs present were well-dispersed on the mesoporous AC and MWCNT supports with small average particle diameters (3–6 nm on mesoporous AC and 1–3 nm on MWCNTs). Conversely, TMNPs agglomerated on the microporous AC supports, resulting in lower dispersion. The different dispersions were reflected in the measured CO uptake on each catalyst; the highest CO uptake was observed on the MWCNT-supported catalyst, followed by the mesoporous-AC-supported catalysts, and the microporous-AC-supported catalysts had the lowest CO uptake. The CO hydrogenation activity followed the same trend due to pore structure of the catalyst supports. The uniform pore size of the MWCNT support led to the best TMNP dispersion, which resulted in the highest number of active sites, giving the highest activity. The MWCNT-supported catalyst also had the highest selectivity for higher alcohols, 16.8%, compared with 13.1% and 12.7% for the two mesoporous-AC-supported catalysts and 12.1% and 11.7% for the two microporous-AC-supported catalysts. The observations suggest that the Brunauer–Emmett–Teller (BET) surface area and pore volume of the supports are not the important features of these catalysts but rather that the textural properties of pore size and percent mesoporosity have the most significant effects on the activity and selectivity.

#### 4.1.2 Fischer–Tropsch Synthesis

Fischer–Tropsch synthesis (FTS) refers to the hydrogenation of carbon monoxide to form hydrocarbons (HCs). This reaction is of environmental importance, as it is a potential route for non-petroleum-based fuel production from waste products (CO and H_2_/H_2_O). The routes of alkene and alkane production are given in eqs 2 and 3, respectively:
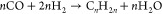
2
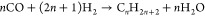
3

First developed in the 1920s by Franz Fischer and Hans Tropsch, Fe-based catalysts were initially favored, although later Co-based catalysts were found to perform well for the hydrogenation of CO to produce a mixture of hydrocarbons and oxygenates.^191^ Vannice studied different transition metals supported on alumina for their specific activities and product distributions for CO hydrogenation and found Ru/Al_2_O_3_ to be the most active catalyst, yielding the highest molecular weight of products.^192^ The wide variety of products possible *via* these routes leads to complicated product mixtures, and therefore, a highly selective catalyst is desirable. The implication of this is that the development of a high-performing trimetallic catalyst could be vastly beneficial.

Badoga *et al.* studied the effects of four different promoters—Mn, Mg, Co, and Ni—on the performance of a KCuFe catalyst supported on mesoporous Al_2_O_3_ for FTS.^193^ The catalysts were synthesized by a sequential incipient wetness impregnation method. First Fe (equivalent to 25 wt %) was added to the Al_2_O_3_, and after drying Cu (equivalent to 0.5 wt %) was added. Then the catalyst was dried again and calcined at 400 °C for 4 h. K was then added (1 wt %), and after drying and calcination the KCuFe/Al_2_O_3_ catalyst was obtained. To this the various promoters could be added by impregnation, and for comparison, an unpromoted monometallic Fe/Al_2_O_3_ catalyst was also synthesized.

The catalysts were reduced for 16 h at 623 K prior to the reaction and tested for FTS at 523 K and 2 MPa with a space velocity of 2000 h^–1^. The unpromoted monometallic Fe/Al_2_O_3_ catalyst showed a high CO conversion of 95% but also a high selectivity for CO_2_ (46.9%). Of all the hydrocarbon products produced, the selectivities were 34.1% for CH_4_, 11.1% for C_2_–C_4_ products, and 54.8% for C_5+_ HCs. The production ratio of olefins to paraffins (O/P) was 0.35. Over the KCuFe/Al_2_O_3_ catalyst, the CO conversion remained high at 94%. However, the C_5+_ selectivity increased in the hydrocarbon product distribution to 77.0%, and the CH_4_ selectivity decreased to 12.0%. The O/P ratio was significantly increased to 0.71, and the CO_2_ selectivity decreased to 44.0%.

When Co was added as a promoter, the CO conversion remained comparable (93%), but the CH_4_ selectivity increased to 16.8% and subsequently the O/P ratio dropped to 0.25. TPR and X-ray absorption near-edge structure (XANES) analyses of this catalyst showed relatively low ease of reduction of Fe compared with other promoted KCuFe/Al_2_O_3_ catalysts. When Mg was added as a promoter, the CO conversion was unaffected (93%), but the O/P ratio decreased with respect to KCuFe/Al_2_O_3_ to 0.60. The CH_4_ selectivity increased from 12.0% to 14.8%, and the C_5+_ HC selectivity decreased from 77.0% to 74.0%. The CO_2_ selectivity was similar for KCuFe/Al_2_O_3_ and the Co- and Mg-promoted equivalents, suggesting that these two promoters are not effective in inhibiting the WGS reaction.

Analysis of the Ni–KCuFe/Al_2_O_3_ catalyst with TPR and XANES suggested that the extent of Fe reduction was high, but the testing data revealed this catalyst to have the lowest CO conversion (77%) and the highest CH_4_ selectivity (29.3%) among all of the bi- and trimetallic catalysts tested. This was thought to be due to the H_2_ spillover mechanism being active on Ni. The Ni–KCuFe/Al_2_O_3_ catalyst did show a reduction in CO_2_ selectivity to 40.7%, which suggests that the WGS reaction was suppressed. However, in turn this lowered the H_2_ availability and may account for the decrease in CO conversion. The Mn–KCuFe/Al_2_O_3_ catalyst was the only promoted catalyst to exhibit an increase in CO conversion versus the unpromoted catalyst to 95%. Both the CO_2_ selectivity (39.4%) and CH_4_ selectivity (10.0%) decreased, whereas the selectivity for the desirable C_5+_ HCs increased to 81.0%. Meanwhile, the C_2_–C_4_ HC selectivity dropped to 9.0%, and the O/P ratio increased to 0.85.

XPS analysis of the Mn–KCuFe/Al_2_O_3_ catalyst showed that it had the highest surface concentration of Fe, whereas the Mg–KCuFe/Al_2_O_3_ catalyst had the lowest surface concentration of Fe (almost 40% lower than that of Mn–KCuFe/Al_2_O_3_), which helps to rationalize the varying performances of these catalysts. TPR and XANES analyses of the Mn–KCuFe/Al_2_O_3_ sample also showed that the ease of Fe reduction on this catalyst and the electron-donating nature of Mn lead to strong electronic interactions between Mn and Fe.

To summarize this work, Mn–KCuFe/Al_2_O_3_ outperforms all of the other catalysts tested with respect to C_5+_ HC selectivity, CO conversion, and minimization of undesired products (CO_2_, CH_4_, and C_2_–C_4_ HCs). This was reported to be due to increased Fe dispersion and reducibility.^193^

Golestan *et al.* also explored the promoting nature of Mn for FTS using unsupported FeCoMn catalysts.^194^ The catalysts were synthesized by a hydrothermal method in which the precursors Fe(NO_3_)_3_·9H_2_O, Co(NO_3_)_3_·6H_2_O, and Mn(NO_3_)_2_·4H_2_O, the capping agent CTAB, and the precipitating agent urea were stirred together for 1 h, after which the mixture was placed in an autoclave and heated to 180 °C for 18 h. The product was washed, collected by centrifugation, dried, and calcined (500 °C, 4 h). The catalysts were synthesized with a range of Mn loadings (wt %), and once the highest-performing catalyst from this series had been identified, the Fe:Co molar ratio was varied to study the impact of this. The catalysts were tested for FTS under the conditions identified as optimum through further study (563 K, 0.2 MPa, and a space velocity of 3000 h^–1^ with a H_2_:CO molar ratio of 1:1).

The desired products for this study were light olefins (C_2_–C_4_), and suppression of CH_4_ production and an improvement in the ratio of olefins produced to methane and paraffins (O/(M+P)) were also intended. It was found that increasing the concentration of Mn improved the selectivity for C_2_–C_4_ olefins and decreased the selectivity for CH_4_; however, the C_5+_ HC selectivity was unaffected. The catalyst containing 16 wt % Mn was the highest-performing with respect to O/(M+P). It was also found that as the Mn loading increased, the surface area, pore size, and pore volume of the catalysts all increased too. The effect of varying the Fe:Co molar ratio was next investigated using a catalyst with 16 wt % Mn. The Fe:Co content was varied from 68 wt % Co and 16 wt % Fe to 16 wt % Co and 68 wt % Fe. The highest-performing catalyst regarding light olefin production was 68%Fe–16%Co–16%Mn. However, although the increasing Fe content and decreasing Co content improved the O/(M+P) ratio, the catalysts with higher Co content had higher activity for syngas conversion.

XRD analysis of the used 68%Fe–16%Co–16%Mn catalyst showed the presence of oxidic and carbide phases, which are active phases for FTS. TPR studies confirmed that the addition of Co reduces the level of Fe_2_O_3_, which is reduced to FeO and Fe and subsequently forms iron carbides, including Fe_5_C_2_, which is especially important in FTS. N_2_ adsorption experiments found the BET surface area of the 68%Fe–16%Co–16%Mn catalyst to be 75 m^2^/g, which is significantly higher than that of any of the other catalysts synthesized and may be a contributing factor to its improved performance.^194^ The same group also studied FeCoMn catalysts supported on MgO, which appeared to improve the activity of the catalyst, although the selectivity for the desired C_2_–C_4_ olefins was reduced.^195^

Razmara *et al.* compared the performance of Co_2_–Ni–Mn/SiO_2_ catalysts prepared by three different methods for the Fischer–Tropsch reaction.^196^ In addition to the Co_2_–Ni–Mn/SiO_2_ catalysts prepared by impregnation and coprecipitation, a third sample was prepared by the thermal decomposition of a novel inorganic precursor, [Ni(H_2_O)_5_Co(dipic)_2_]·2H_2_O + [Mn(H_2_O)_5_Co(dipic)_2_]/SiO_2_, where dipic denotes the ligand pyridine-2,6-dicarboxylic acid. Thermogravimetric analysis results showed that the precursor decomposed to the metal oxides NiO_4_ and MnCo_2_O_4_ at 427 °C, so in order to ensure full decomposition, the precursor was calcined at 500 °C for 4 h in static air. The calcination process was shown to significantly increase the measured BET surface area and pore volume in the nanocatalyst compared with the precursor. This was reflected in the morphology observed by scanning electron microscopy (SEM), where the catalyst particle size is visibly much smaller than the precursor.

The three catalysts produced by thermal decomposition, coprecipitation, and impregnation were tested for the Fischer–Tropsch reaction, conducted at 553–633 K and atmospheric pressure with a H_2_:CO molar ratio of 1:1 (gas hourly space velocity (GHSV) = 3600 h^–1^). It was found that over all three samples, as the reaction temperature was increased, the activity for CO conversion also increased, but this also corresponded to an increase in methane selectivity. At all of the temperatures tested, the catalyst produced by thermal decomposition was the highest-performing with respect to both CO conversion and hydrocarbon selectivity. At 633 K, the CO conversions for the thermal decomposition, coprecipitation, and impregnation catalysts were 68.7, 58.1, and 43.2% respectively. This directly correlates with the Debye–Scherrer calculations from XRD, which gave crystallite sizes of 12.5, 21.3, and 29.0 nm for the thermal decomposition, coprecipitation, and impregnation catalysts, respectively. This is also in agreement with the observations from SEM and surface area measurements and suggests that the smaller crystallite size of the thermal decomposition catalyst exposes a larger amount of surface area, thereby improving the catalyst activity. All three catalysts showed stable performance over 8 h of time on-line, but after that there was a decrease in CO conversion over the 30 h reaction, with final CO conversion values of 61, 47, and 33% for the thermal decomposition, coprecipitation, and impregnation catalysts, respectively. In addition to its relative superior performance, other advantages of the thermal decomposition method were noted as being the accurate metal loading on the support, a homogeneous metal distribution, and the formation of a strong metal–support interaction.^196^

Zhai *et al.* used zinc- and sodium-modulated Fe_5_C_2_ catalysts synthesized by coprecipitation to investigate the formation of C_5+_ alkenes *via* FTS.^197^ In Fe-based catalysts, Zn may often be used to inhibit the reduction of FeO to increase CO adsorption, hence increasing the formation of long-chain hydrocarbons. However, the WGS reaction remained prevalent, and the methane productivity was high. Potassium is commonly used to increase the hydrogenation barrier of CH_*x*_ species, subduing methane production and increasing the selectivity for other products, including alkenes. By using sodium instead, the authors hoped to show a similar effect.

The catalysts tested were denoted as Fe–Zn–*x*Na, where *x* is the concentration of Na (3.4, 0.81, 0.36, or 0.0%) and the concentrations of Fe and Zn were equal. Furthermore, a catalyst containing no Zn and 1.2% Na (Fe–1.2Na) was also tested, along with a version of this catalyst that was washed to remove the Na, leaving a Na-free Fe catalyst. XRD characterization of the catalysts showed similar diffraction patterns for all of the Fe–Zn–Na catalysts, with the ZnO and ZnFe_2_O_4_ phases dominating, illustrating the strong Zn and Fe interactions in these samples. The most intense ZnO diffraction pattern was observed in the catalyst with the highest concentration of Na. In the catalysts in which Zn was absent, the crystallite size increased from the range 8.4–13.0 nm to 15.4–19.6 nm. This was supported by observations from the CO uptake survey, which suggested a larger exposed surface for the catalysts containing Zn.

**Figure 12:**
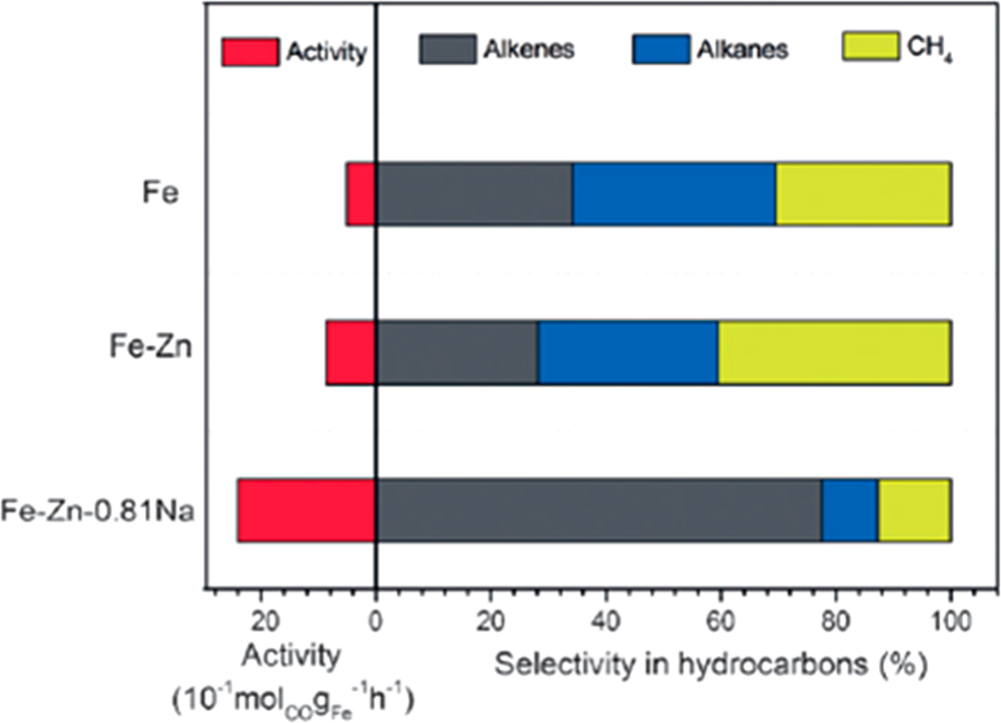
Activities and product distributions on Fe–Zn–0.81Na, Fe–Zn, and Fe catalysts. Reaction conditions: catalyst (20 mg), 340 °C, 2.0 MPa, syngas (6:16:2:1 CO/H_2_:/CO_2_/Ar, 20 mL min^–1^). Reproduced with permission from ref (197). Copyright 2016 Wiley-VCH.

The catalysts were tested for FTS at 340 °C and 2.0 MPa with a 6:16:2:1 H_2_/CO/CO_2_/Ar synthesis gas mixture. The catalyst found to have the highest syngas conversion was Fe–Zn–0.36Na, followed by Fe–Zn–0.81Na, Fe–Zn–3.4Na, and Fe–1.2Na, and finally the monometallic Fe catalyst had the lowest conversion. The two catalysts containing no Zn performed much worse than the Zn-containing catalysts, as Zn acts as a structural promoter leading to smaller Fe crystals with more exposed surface, which corresponds to a greater activity with respect to the mass of Fe. Figure 12 compares the performance of tri-, bi-, and monometallic catalysts.

It is common for Fe-based catalysts to produce large amounts of CO_2_ in FTS, and this was indeed observed over the Fe–Zn catalyst (33.1%). However, for the Na-containing catalysts the CO_2_ selectivity was reduced to as low as 21.9% (Fe–1.2Na) and 22.5% for the best-performing trimetallic catalyst (Fe–Zn–3.4Na). Fe–Zn–0.81Na also showed the lowest selectivity for CH_4_ among all of the catalysts tested. Increasing the Na content suppressed the alkane formation and increased the alkene production. Fe–Zn–3.4Na had the greatest production of C_5+_ alkenes (46.3% selectivity). Fe–1.2Na and Fe–Zn–0.81Na, the two catalysts with the most comparable Na contents, showed similar product distributions, but Fe–1.2Na had much lower syngas conversion. This indicates that Na plays an important role in determining the product distribution. DFT studies were used to show that Na is an electronic promoter, donating electrons to Fe to increase the CO activation ability and decrease the rate of C=C bond hydrogenation by promoting desorption of the alkene.^197^

There are other synthetic fuels that can be produced from the hydrogenation of CO, such as ethanol. Liu *et al.* studied the effects of the addition of Fe and Mn to Rh/SiO_2_ catalysts for the production of C_2+_ oxygenates and short chain hydrocarbons from syngas.^198^ The reactant mixture had a CO:H_2_ ratio of 1:1. The performances of mono-, bi-, and trimetallic combinations of the three metals were compared. To produce monometallic 5 wt % Rh/SiO_2_, 0.41 wt % Fe/SiO_2_, and 0.27 wt % Mn/SiO_2_ catalysts for comparison purposes, an incipient wetness impregnation method was used. The metal precursors were RhCl_3_, Fe(NO_3_)_3_, and Mn(NO_3_)_2_, respectively. To produce the bimetallic and trimetallic catalysts, controlled surface reaction (CSR) processes were used. For RhFe/SiO_2_ or RhMn/SiO_2_ catalysts, the reduced Rh/SiO_2_ catalyst was held in a Schlenk flask under an inert atmosphere, and (cyclohexadiene)iron tricarbonyl or (cyclohexane)manganese tricarbonyl dissolved in *n*-pentane was added. After 2 h of stirring, the excess pentane was evaporated, and the catalyst was then reduced. For RhFeMn/SiO_2_ or RhMnFe/SiO_2_ catalysts, the third metal was added to the reduced RhFe/SiO_2_ or RhMn/SiO_2_ catalysts *via* a Schlenk line using the same process as described for the production of bimetallic catalysts. The catalysts are denoted as Rh–*x*Fe–*y*Mn, where *x* and *y* are the nominal Fe:Rh and Mn:Rh molar ratios, respectively. The Rh loading was consistent in all of the catalysts at 5 wt %.

UV–vis spectroscopy was used to characterize the catalysts, and it was found that neither Fe nor Mn interacted with the silica support and that the Rh–Fe and Rh–Mn interactions were much more significant. The extent of metal uptake from CSR was also assessed; for the trimetallic catalysts, when Fe is deposited before Mn, the percentage of Mn adsorbed decreased as the total metal loading increased. That is to say, deposition of the Mn precursor is limited by the number of Rh sites available after Fe deposition. For example, in the Rh–0.05Fe–0.10Mn catalyst, the Mn uptake was measured as 63%. When the iron content was tripled in the Rh–0.15Fe–0.10Mn catalyst, the Mn uptake decreased to 48%. A further increase in iron loading to Rh–0.30Fe–0.10Mn saw an uptake of Mn of only 20%. Likewise, when Mn was deposited first in the trimetallic catalyst, the Fe loading was found to be lower than anticipated. This suggests that in the trimetallic system Fe and Mn act independently over the Rh surface, apart from when the loading of the first metal is very low, leaving enough Rh sites available for the deposition of the second metal.

The catalysts were tested for CO conversion at 523 K and 4.0 MPa with a CO:H_2_ ratio of 1:1. The CO conversion was kept close to 1% for all of the tests in order to study the product selectivity over each catalyst in detail. Over the monometallic Rh/SiO_2_ catalyst, the major products were CH_4_ and acetaldehyde. The Fe/SiO_2_ catalyst was much less active than Rh/SiO_2_ and primarily produced C_2+_ hydrocarbons, while the Mn/SiO_2_ catalyst was completely inactive.

The bimetallic RhFe/SiO_2_ catalysts were synthesized with Rh:Fe ratios of 0.05, 0.15, and 0.30. It was found that the selectivity for ethanol increased up to the Rh–0.15Fe catalyst and the selectivity for acetaldehyde decreased, and the Rh–0.15Fe catalyst had the highest selectivity for total oxygenates among all of the mono- and bimetallic catalysts tested. In fact, the Rh–0.30Fe catalyst had the highest TOF for total oxygenate production; however, as the TOF for CH_4_ production also increased, the overall selectivity for oxygenates decreased. It appears that the promoting role of Fe facilitates acetaldehyde hydrogenation: as the loading of Fe is increased, the TOF for acetaldehyde production decreases and the TOF for ethanol production increases.

The bimetallic RhMn/SiO_2_ catalysts had Rh:Mn ratios of 0.05, 0.10, and 0.15. The Rh–0.10Mn catalyst showed the highest selectivity for ethanol. This catalyst had less of a promotional effect than Fe, but Rh–Mn interactions still resulted in increased selectivity for oxygenates compared with the monometallic catalysts. The Rh–Mn catalysts are much more selective for C_2+_ hydrocarbons (2 times more than Rh/SiO_2_ and 4 times more than RhFe/SiO_2_) and showed a larger decrease in selectivity for CO_2_ and CH_4_ compared with Rh/SiO_2_ and RhFe/SiO_2_. Rh–0.10Mn had the highest selectivity for total oxygenates (28.4%), and the TOF for CO consumption was 4 times higher compared with Rh/SiO_2_—the highest of any of the catalysts tested in this study. The promotional effect observed for the Rh–Mn interactions is related to the increase in C_2+_ hydrocarbon production and CO consumption.

The synergistic effect between both the Fe and Mn promoters and Rh can be examined by testing the trimetallic catalysts. First, the amount of Fe was kept fixed while the level of Mn was varied. For the series Rh–0.05Fe–*y*Mn (*y* = 0.05, 0.10, 0.15), the methane and CO_2_ production levels were reduced, and the C_2+_ hydrocarbon and ethanol production levels were improved. Rh–0.05Fe–0.10Mn was found to have the best selectivity for total oxygenates (36.8%). The C_2_ oxygenate formation rate and CO consumption rate increased as the Mn loading increased. In general, this series of catalysts was found to favor production of alkenes over alkanes, which was the same trend as observed over the bimetallic Rh–Mn catalysts. For the series Rh–0.15Fe–*y*Mn, selectivity patterns similar to those for the Rh–0.05Fe–*y*Mn catalysts were observed, with Rh–0.15Fe–0.10Mn showing the highest selectivities for ethanol, C_2_ oxygenates, and total oxygenates. This catalyst was also the most selective for oxygenates among all of the catalysts prepared and had the lowest methane selectivity. However, its CO consumption rate was low. Oxygenates accounted for 44.8% of the products produced by this catalyst (2 times more than over Rh/SiO_2_). Conversely, Rh–0.30–*y*Mn showed a different pattern of selectivity compared with the previously tested trimetallic catalysts. For this series it was found that changing the Mn loading did not substantially change the reactivity of the catalyst, as Fe was in excess first, limiting Rh–Mn interactions. Therefore, in general, the Rh–0.30–*y*Mn catalysts followed the same trends in selectivity as observed for the Rh–*x*Fe catalysts.

**Figure 13:**
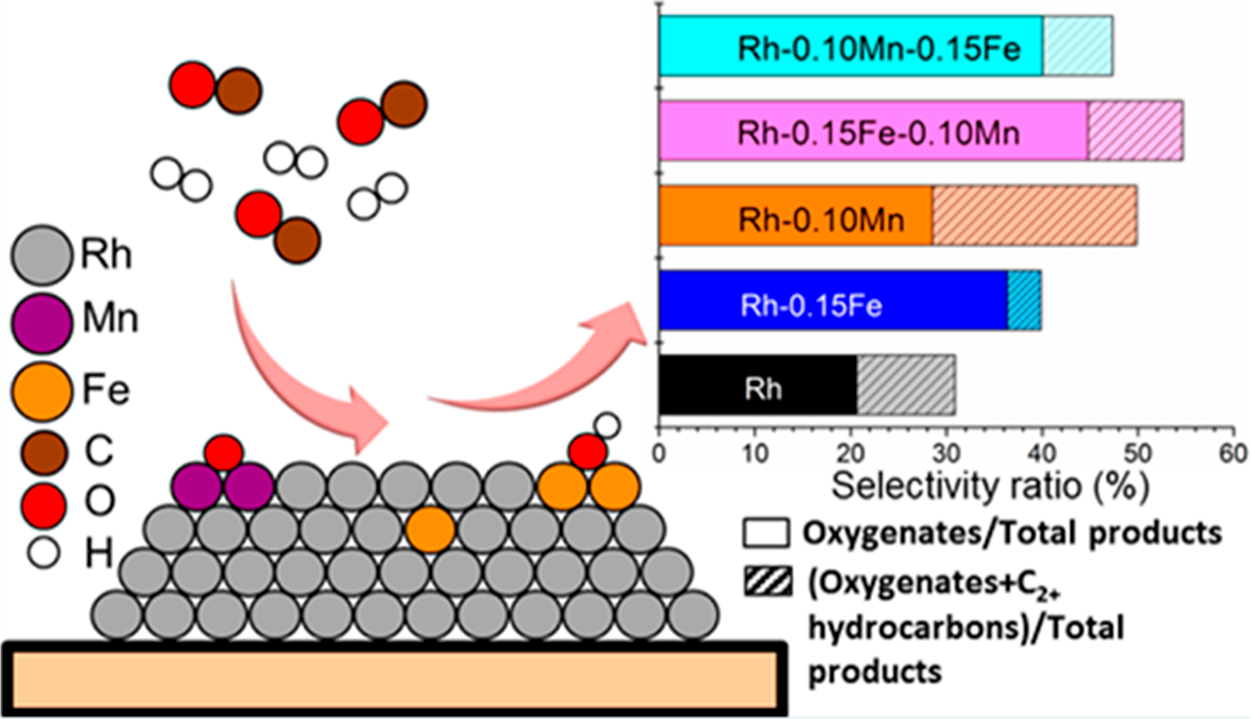
Active sites for CO hydrogenation on Rh–Mn and Rh–Fe active sites and product distributions for various Rh–*x*Fe–*y*Mn catalysts tested for syngas conversion. Reproduced from ref (198). Copyright 2017 American Chemical Society.

The effect of the order of secondary metal deposition on the catalytic performance was also investigated (Figure 13). Rh–0.15Fe–0.05Mn and Rh–0.05Mn–0.15Fe were found to perform similarly. However, the Rh–0.15Fe–0.10 Mn and Rh–0.10Mn–0.15Fe catalysts and the Rh–0.15Fe–0.15Mn and Rh–0.15Mn–0.15Fe catalysts showed more differences in reactivity for CO hydrogenation. When Fe was deposited before Mn, the total production of oxygenates was larger. When Mn was deposited before Fe, the ethanol selectivity was lowered, and the C_2+_ hydrocarbon selectivity was increased. This is the same pattern as observed for the Rh–*x*Fe and Rh–*y*Mn catalysts, respectively, and shows that the effect of the precursor deposited first is more important to the overall performance of the catalyst compared with the precursor deposited last. Observations made in an analysis of the uptake of the second precursor showed this to be less than 100%. However, this does not apply for the catalysts with very low loadings of Mn (*i.e.*, Rh–0.15Fe–0.05Mn and Rh–0.05Mn–*x*Fe).^198^

#### 4.1.3 Hydrogenation of CO_2_ to Methanol

The use of CO_2_ to produce platform chemicals is emerging as an important process to reduce harmful greenhouse gas emissions. Of these valorization products, methanol has been identified as a valuable option because it can be used as a solvent or as a chemical feedstock. Methanol can also be produced as a renewable fuel, provided that sustainable H_2_ is available for the hydrogenation of CO_2_.^199^ Ramirez *et al.* investigated the hydrogenation of CO_2_ under atmospheric pressure over PdCuZn/SiC trimetallic catalysts with various palladium metal loadings (Table 2).^200^ The bimetallic combinations PdCu/SiC, PdZn/SiC, and CuZn/SiC were also compared for their catalytic performance. The methanol synthesis reaction (eq 4) is in competition with the reverse water gas shift (RWGS) reaction (eq 5):
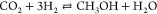
4
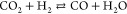
5

**Table 2:** Summary of Catalysts Discussed Here for the Hydrogenation of CO_2_ to Methanol

catalyst	catalyst type	reaction conditions	TOF;^a^ activity;^b^ selectivity	ref
Cu/ZnO/Al_2_O_3_ (CZA)	commercial	*T* = 513 K; *p* = 2 MPa; H_2_:CO_2_ = 3; GHSV = 6000 h^–1^	activity = 3.17; selectivity for MeOH = 35%	(205)
CuZn/SiC	BMNP	*T* = 523 K; *p* = 0.1 MPa; CO_2_:H_2_ = 1:9 v/v	activity = 0.03; selectivity for MeOH = 5%	(200)
PdZn/SiC			activity = 0.23; selectivity for MeOH = 21%	
PdCu/SiC			activity = 0; selectivity for MeOH = 0%	
12.5PdCuZn/SiC	TMNP		activity = 0.07; selectivity for MeOH = 23%	
25PdCuZn/SiC			activity = 0.14; selectivity for MeOH = 40%	
37.5PdCuZn/SiC			activity = 0.30; selectivity for MeOH = 50%	
Ni–Ga	BMNP	*T* = 533 K; *p* = 1 MPa; H_2_:CO_2_ = 3	TOF = 2880; activity = 1.2; selectivity for MeOH + DME = 43% (DME selectivity < 1%)	(203)
Au–Ni–Ga	TMNP		TOF = 11160; activity = 1.1; selectivity for MeOH + DME = 51% (DME selectivity < 1%)	
Cu–Ni–Ga			TOF = 5040; activity = 1.8; selectivity for MeOH + DME = 56% (DME selectivity < 1%)	
Co–Ni–Ga			TOF = 1800; activity = 1.3; selectivity for MeOH + DME = 55% (DME selectivity < 1%)	

a TOF (h^–1^);

b Specific activity toward MeOH formation (mol_MeOH_ kg_cat_^–1^ h^–1^).

These catalysts were prepared by an incipient wetness impregnation method and were shown to have Type II–IV isotherms, correlating to macro- and mesoporous materials that were unchanged from the β-SiC support. However, the surface area, pore volume, and average pore radius were all reduced following the addition of the metal nanoparticles. For the bimetallic CuZn/SiC catalyst, the pores were fully blocked as a result of the larger size of the nanoparticles; however, in the palladium-containing bi- and trimetallic nanoparticles, the particle size was smaller, so the pores in the support were only partially blocked, reducing the average pore size. In general, it was observed that the particle diameter decreased as the palladium content increased, as confirmed through studying XRD patterns and applying the Scherrer equation to estimate the particle sizes. The average particle sizes for the trimetallic catalysts containing 25 mol % and 37.5 mol % Pd were smaller than those for the bimetallic PdZn/SiC catalyst. TEM studies showed a Gaussian particle size distribution for all of the catalysts; however, the smallest average particle diameter was for the trimetallic catalyst containing the highest Pd content (37.5 mol %). Through TPR experiments, the reduction peak representing the formation of Cu^0^ was observed to occur at lower temperatures for the trimetallic catalysts than for the CuZn/SiC catalyst. The TPR profiles also showed a peak at around 673 K for the trimetallic catalyst, corresponding to PdZn alloy formation. As the CO_2_ hydrogenation reaction temperature was increased, the CO formation rate increased. The highest rate for the RWGS reaction was measured over the PdZn/SiC catalyst, followed by the CuZn/SiC catalyst. Maximum methanol formation at atmospheric pressure was generated in the temperature range 498–548 K. The methanol formation rate increased with the palladium content of the TMNP catalysts. Although the Cu-containing bimetallic catalysts performed poorly, the trimetallic catalysts were more active, which highlights the potential of the synergistic effects among the three metals to achieve high selectivity and activity. It must be noted, however, that the methanol production by these catalysts at atmospheric pressure is much less than has been obtained at thermodynamic equilibrium.

The combination of Pd, Zn, and Cu is thought to be effective for CO_2_ hydrogenation because of the formation of a PdZn alloy, which is considered to be an active site for methanol synthesis. Conversely, metallic Pd^0^ is present in the active surface at which the RWGS reaction occurs. The addition of Cu prevents the formation of metallic Pd^0^ and hence decreases the rate of CO formation and increases the selectivity for methanol.^200^ Furthermore, as reported by Zhao *et al.*,^201^ DFT calculations on PdCuAu TMNPs with various compositions indicated that the average bond distance of Pd_*a*_Cu_*b*_Au_*c*_ clusters, (where *a*:*b*:*c* is the compositional ratio), decreases with increasing Cu content. Hydrogen adsorption is favored by Pd > Cu > Au, highlighting the role of the Pd atoms in these trimetallic catalyst compositions. Pd_3_Cu_2_Au_2_ exhibited the highest H_2_ adsorption energy and was deemed to be the most suitable for H–H activation among the studied catalysts.^201^ When Cu and Au are mixed with Pt instead of Pd, the TMNPs are active toward the WGS reaction. Xue *et al.*(202) showed Au@o-AuCuPt to be most stable among the TMNP catalysts examined for the WGS reaction, with greater stability than respective binary alloys. The increased electronic activity was explained in terms of the d-band center of −3.05 eV being closer to the Fermi level. Both reactants (CO and H_2_O) bind strongly to the catalyst surface, and exhaustive mechanism analysis has shown that the formic acid mechanism has a lower barrier (3.09 eV) than the redox (4.84 eV) and carboxyl (3.15 eV) routes. The elementary reaction energy barrier of CO and desorption of chemisorbed CO_2_ is large, which was attributed to the strong adsorbate interaction with Pt.

Metal-promoted NiGa/SiO_2_ intermetallic catalysts, prepared *via* incipient wetness impregnation, have also been reported for CO_2_ hydrogenation.^203^ The activity order for CO_2_ hydrogenation to methanol (1.0 MPa, 473–543 K) as a function of TOF was AuNiGa > CuNiGa > NiGa > CoNiGa. The supported TMNP AuNiGa/SiO_2_ exhibited the highest TOF in this study (>3 s^–1^ at 533 K), which was a 4-fold improvement over o-NiGa/SiO_2_. The promotional effect of Au weakened the interaction between the catalyst surface and adsorbates binding through oxygen or carbon and was considered to be due to electronic modification of adding Au to NiGa. The active center for the high activity over AuNiGa is thought to be Au decorated on Ni_3_Ga, as supported by CO chemisorption, XRD, and STEM-EDX analyses. CuNiGa outperformed the other catalysts in terms of specific activity toward methanol; however, AuNiGa was more active. The addition of Cu is thought to form a Cu–Ga phase, affecting the selectivity.^203^ Further work will be needed to understand the intrinsic activities of the phases and the correlation to changes in catalytic performance. Other Ga-based catalysts have been prepared and tested for CO_2_ hydrogenation to methanol. With an optimized composition, Ni_2_FeGa catalysts were prepared by incipient wetness impregnation with an optimal reduction temperature of 823 K, resulting in alloy formation of the three metals and no evidence of sintering at this temperature. The introduction of Ga into NiFe NPs resulted in higher methanol selectivity through a promotional effect;^204^ however, the as-prepared Ni_2_FeGa/SiO_2_ did not outperform the commercial Cu/ZnO/Al_2_O_3_ (CZA) catalyst that is commonly used for methanol synthesis. Trimetallic alloyed systems could be desirable to deviate away from problems associated with CZA, such as water formation from the RWGS reaction, which promotes Cu sintering. Therefore, further investigation into suppressing the RWGS reaction and combining the favorable formation of a PdZn alloy has the potential to optimize CO_2_ hydrogenation to methanol.

#### 4.1.4 Direct Synthesis of Hydrogen Peroxide

TMNPs have recently been applied to the direct formation of hydrogen peroxide from H_2_ and O_2_. Industrially, H_2_O_2_ is produced by the anthraquinone method, and in 2014 the global annual production was 3 million metric tonnes, most of which was used for fine chemical synthesis and bleaching of paper and textiles.^206^ The anthraquinone method is indirect and involves hydrogenation and oxidation of an alkylanthraquinone precursor in organic solvents; an extraction step is also required to recover the H_2_O_2_. The multistep method generates significant waste and comes with high transport costs.^206^ Direct H_2_O_2_ synthesis is a potential alternative production route, but a major challenge is obtaining a high selectivity over water and preventing further hydrogenation of H_2_O_2_ to water.

Recent studies have investigated the use of supported Pd-based catalysts for the direct formation of H_2_O_2_;^207−210^ however, these materials require promoters (typically acid and halide) to obtain high selectivity.^211^ Hutchings *et al.* used multimetallic NPs for the direct formation of H_2_O_2_.^212^ The research team determined that the addition of Au to the supported Pd catalysts resulted in high H_2_O_2_ selectivity, eliminating the need for promoters. However, significant overhydrogenation of H_2_O_2_ to water was still observed. To further improve the yield of H_2_O_2_, the addition of Pt to form a TMNP-based catalyst was investigated.

The study by Hutchings *et al.* set out to determine an optimal Au:Pd:Pt stoichiometry when supported on CeO_2_.^16^ The TMNPs were prepared using an impregnation method, where the total metal content was fixed at 5 wt % and mono- and bimetallic analogues were also prepared. For catalytic testing, the total pressure of the reactor was 4 MPa, and the temperature was kept at 275 K. Each experiment was run for 30 min. The catalytic activity of each trimetallic composition can be seen in Figure 3, given in the form of a contour map. In general, the catalysts consisted of equal parts Pd and Au with a small quantity of Pt added. The best catalyst would exhibit a high H_2_O_2_ productivity rate (PR) with a low rate of H_2_O_2_ hydrogenation to water. Of the monometallic analogues supported on CeO_2_, Pd showed the highest PR of 97 mol_H_2_O_2__ kg_cat_^–1^ h^–1^. Supported monometallic Pt and Au achieved PRs of 8 and 1 mol_H_2_O_2__ kg_cat_^–1^ h^–1^, respectively. The PRs for the hydrogenation of H_2_O_2_ to water observed over Pd, Pt, and Au were 329, 126, and 118 mol_H_2_O_2__ kg_cat_^–1^ h^–1^, respectively. For each of these catalysts, PR_product_/PR_hydrog_ is less than 1, which is undesirable. Bimetallic Au_50_Pd_50_/CeO_2_ exhibited a PR of 68 mol_H_2_O_2__ kg_cat_^–1^ h^–1^; however, the hydrogenation PR was 145 mol_H_2_O_2__ kg_cat_^–1^ h^–1^, again resulting in a PR_product_/PR_hydrog_ lower than 1. Of the trimetallic compositions consisting of equal-weight quantities of Pd and Au with a small (<10%) weight fraction of Pt, the PR was generally over 100 mol_H_2_O_2__ kg_cat_^–1^ h^–1^. The highest H_2_O_2_ PR_product_ of 170 mol_H_2_O_2__ kg_cat_^–1^ h^–1^ was observed with Au_48_Pd_48_Pt_4_/CeO_2_. The corresponding hydrogenation PR_hydrog_ was 145 mol_H_2_O_2__ kg_cat_^–1^ h^–1^, giving a PR_product_/PR_hydrog_ ratio greater than 1. Compositions with more and less Pt saw a decline in PR_product_, but the PR_product_/PR_hydrog_ ratio was *ca.* 1.1–1.5.

A further composition was investigated whereby a portion of the Pd was replaced with Pt: Au_50_Pd_46_Pt_4_/CeO_2_.^16^ The catalyst showed a lower H_2_O_2_ PR of 86 mol_H_2_O_2__ kg_cat_^–1^ h^–1^; interestingly, however, this composition highly suppressed the hydrogenation reaction, with a hydrogenation PR of just 11 mol_H_2_O_2__ kg_cat_^–1^ h^–1^. This resulted in a PR_product_/PR_hydrog_ ratio of almost 8, or a selectivity of 88%. Two other regions on the compositional contour map also showed high productivity of H_2_O_2_, most notably an almost-entirely Pd composition with some Pt and Au added and a region that was predominantly Pd but with more Pt and Au added. However, catalysts prepared within these compositional regions also showed high hydrogenation and decomposition PRs. Only in the region of nearly equal Au and Pd with a minor concentration of Pt were simultaneous promotion of H_2_O_2_ production and suppression of hydrogenation and decomposition observed. Thus, Au_50_Pd_46_Pt_4_/CeO_2_ was concluded to be the optimal composition for the direct synthesis of H_2_O_2_. As the optimal catalyst had a lower H_2_O_2_ productivity than the other trimetallic formations yet yielded the highest selectivity, this outcome once again demonstrates the trade-off between total activity and high selectivity that is often observed within the field of catalysis.

Following the impregnation synthesis of each catalyst, a 673 K heat treatment in air was carried out. XPS data showed that following heat treatment the Pd:Au molar ratio on the surface of the TMNPs was far greater than the theoretically expected value.^16^ Au_50_Pd_46_Pt_4_/CeO_2_ was expected to have a Pd:Au molar ratio of 1.7; however, experimental data proved the surface ratio to be 165, nearly 100 times greater, which is indicative of a core@shell structure with a Pd shell. Interestingly, Au_50_Pd_50_/CeO_2_ saw a far smaller change in the Pd:Au molar ratio from 1.9 to 7.1. This leads to the possibility that the presence of Pt induces a drastic reorganization of the surface and reconfiguration of the structure of the nanoparticle. As with many other TMNP systems, the electronic induction between the three metals was thought to drive the enhanced catalytic performance toward the production of H_2_O_2_. The influence of the support on the efficacy of Pd–Pt–Au mixtures can be compared with supporting research on TiO_2_ by the same group.^213^ Again, the inclusion of a low concentration of Pt in the AuPd NPs resulted in the highest PR_product_/PR_hydrog_ ratio of 1.14 in this study, over 2.45%Au–2.45%Pd–0.05%Pt/TiO_2_, and increasing this Pt concentration decreased the PR ratio (Table 3). Analysis of the catalysts prepared by impregnation indicated that the distribution of NP diameters narrowed with the inclusion of Pt. There was a significant quantity of smaller Pd-rich NPs, and the Pd concentration in the nanoalloys decreased as the particle diameter increased. Interestingly, the inclusion of Pt suppresses the core@shell motif common with AuPd BMNPs supported on TiO_2_.

**Table 3:** Summary of Mono-, Bi- and Trimetallic NP Catalysts for the Direct Synthesis of H_2_O_2_ from H_2_ and O_2_

catalyst	catalyst type (supported)	H_2_O_2_ formation rate/mol_H_2_O_2__ h^–1^ kg_cat_^–1^ (H_2_ selectivity/%)^a^	rate of overhydrogenation (mol_H_2_O_2__ h^–1^ kg_cat_^–1^)	PR_product_/PR_hydrog_	ref
5%Au/C	MMNP	1.0	ND^b^	–	(212)
2.5%Au–2.5%Pd/C	BMNP	110 (80)	28	3.92	
5%Pd/C	MMNP	55 (34)	107	0.51	
5%Au/Al_2_O_3_	MMNP	2.6	ND^b^	–	
2.5%Au–2.5%Pd/Al_2_O_3_	BMNP	15 (14)	92	0.16	
5%Pd/Al_2_O_3_	MMNP	9.0	ND^b^	–	
5%Au/TiO_2_	MMNP	7.0	ND^b^	–	
2.5%Au–2.5%Pd/TiO_2_	BMNP	64 (70)	27	2.37	
5%Pd/TiO_2_	MMNP	30 (21)	113	0.26	
2.5%Au–2.5%Pd/TiO_2_ (1:1.9 mol % Au:Pd)	BMNP	64	235	0.27	(213)
2.5%Au–2.5%Pt/TiO_2_ (1:1 mol % Au:Pd)	BMNP	65	103	0.63	
2.5%Pd–2.5%Pt/TiO_2_ (1.9:1 mol % Au:Pd)	BMNP	124	129	0.96	
2.45%Au–2.45%Pd–0.05%Pt/TiO_2_ (1:1.9:0.025 mol % Au:Pd:Pt)	TMNP	154	135	1.14	
2.45%Au–2.45%Pd–0.1%Pt/TiO_2_ (1:1.9:0.05 mol % Au:Pd:Pt)	TMNP	159	283	0.56	
2.40%Au–2.40%Pd–0.20%Pt/TiO_2_ (1:1.9:0.10 mol % Au:Pd:Pt)	TMNP	156	318	0.49	
2.28%Au–2.28%Pd–0.45%Pt/TiO_2_ (1:1.9:0.20 mol % Au:Pd:Pt)	TMNP	106	263	0.40	
0.20%Au–4.60%Pd–0.20%Pt/TiO_2_ (1:43:1 mol % Au:Pd:Pt)	TMNP	184	382	0.48	
Pd/CeO_2_ (5 wt % Pd)	MMNP	97	329	0.29	(16)
Pt/CeO_2_ (5 wt % Pt)	MMNP	8	126	0.063	
Au/CeO_2_ (5 wt % Au)	MMNP	1	118	0.0084	
Au_50_Pd_50_/CeO_2_ (5 wt % M)	BMNP	68	145	0.47	
Au_48_Pd_48_Pt_4_/CeO_2_ (5 wt % M)	TMNP	170	145	1.17	
Au_50_Pd_46_Pt_4_/CeO_2_ (5 wt % M)	TMNP	86	11	7.81	
PdAg/C	BMNP	70	29	2.41	(216)

a Reaction conditions: *T* = 275 K; *p* = 4 MPa; H_2_:O_2_ ratio = 1:2.

b ND = not determinable at such low yields.

There have been several other reports of direct H_2_O_2_ synthesis using MNP catalysts.^214,215^ In general, the PR_product_ over BMNPs was reported to be lower than that over the TMNP formulations, typically in the 50–100 mol_H_2_O_2__ kg_cat_^–1^ h^–1^ region, with selectivities of <75%. The Au_50_Pd_46_Pt_4_/CeO_2_ catalyst has a PR of 86 mol_H_2_O_2__ kg_cat_^–1^ h^–1^, similar to the PR_product_ of 70 mol_H_2_O_2__ kg_cat_^–1^ h^–1^ for the bimetallic PdAg/C catalyst reported by Gu *et al.*;^216^ however, the selectivity was 71%, which was lower than the 88% selectivity obtained with Au_50_Pd_46_Pt_4_/CeO_2_.

Aside from earlier publications by the Hutchings team, which are summarized in Table 3 (*e.g.*, trimetallic ruthenium-containing PdRuAu/TiO_2_ catalysts with activity and selectivities similar to those for the AuPdPt formulations),^217^ to our knowledge there are no other reports of TMNPs for this reaction. Thus, the use of TMNPs for the direct synthesis of H_2_O_2_ is a new and promising area of investigation.

#### 4.1.5 Selective Hydrogenation of Alkynes to Alkenes

TMNPs have also been investigated for application in the selective hydrogenation of phenylacetylene to styrene. Industrially, styrene is an important monomer unit used for the production of polystyrene, 22 million tonnes of which was produced in 2015.^218^ For this reaction, the catalyst must simultaneously inhibit overhydrogenation to ethylbenzene.^165^

**Figure 14:**
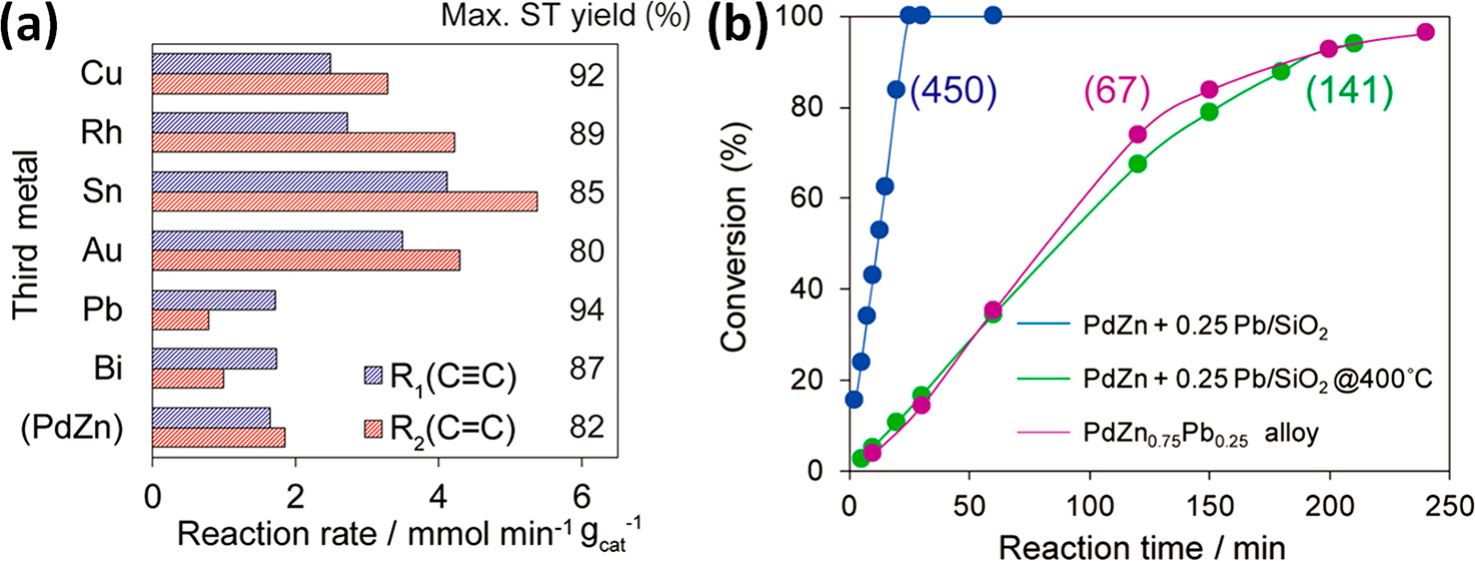
(a) Catalytic performance data for PdZn with several third metals added *via* galvanic replacement. (b) Conversion of phenylacetylene as a function of reaction time under the reaction conditions, treated at 673 K. Data for an unsupported PdZn_0.75_Pb_0.25_ alloy are provided for comparison. Corresponding TOF values are given in brackets. Adapted from ref (165). Copyright 2019 American Chemical Society.

Miyazaki *et al.* reported several TMNPs based on o-PdZn/SiO_2_ in which a third metal (M = Cu, Rh, Sn, Au, Pb, or Bi) was added *via* galvanic replacement, and the catalysts were compared on the basis of their catalytic activities (Figure 14).^165^ In the context of the reaction, it is important that reaction rate *R*_1_ (the rate of formation of styrene) is higher than *R*_2_ (the rate of formation of ethylbenzene) to ensure selectivity for styrene over ethylbenzene. The compositions with the highest reaction rate were M = Au, Sn, Rh, and Cu, but these compositions exhibited a greater propensity for overhydrogenation to form ethylbenzene. Furthermore, supported PdZn favors the formation of ethylbenzene over styrene. The inclusion of Pb or Bi, however, favored the formation of styrene, with Pb producing the highest yield of styrene (Figure 14a). The authors suggested that the product preference for styrene is due to steric hindrance because the large atomic radii of Pb and Bi atoms suppress further hydrogenation of the C=C moiety. This observation was supported by previous work by this group whereby overhydrogenation of internal C=C bonds was suppressed as a result of steric repulsion in reactions where large Bi atoms were present on the surface.^219^ The addition of Pb to the PdZn surface causes different surface structures to form according to DFT calculations; these substituted surfaces appear to increase the length of the Pd–C bond in ethylene over that of acetylene. To demonstrate the steric hindrance effect, other compositions were prepared such that Pb_*x*_ with *x* = 0.1–0.4. The *R*_1_/*R*_2_ ratio was reported to increase with increasing quantities of Pb, but the rates of both hydrogenation reactions were diminished.

The TOF over the PdZn@Pb_0.25_/SiO_2_ catalyst was 450 min^–1^, in comparison with 273 min^–1^ over PdZn. The TOF over the commercial catalyst for the same reaction, PdPb/CaCO_3_ (Lindlar catalyst) was 429 min^–1^. Upon heating to 673 K, however, the TOF of PdZn@Pb_0.25_/SiO_2_ diminished to 141 min^–1^. The diminished TOF was reportedly due to a phase change that resulted in the loss of the AB@AC structure and the formation of a random PdZnPb alloy. The partial hydrogenation to styrene demonstrates the trade-off between reaction rate and selectivity, as the compositions with the highest conversion rates had the lowest selectivity for the desired product; however, the superior TOF, selectivity, and yield over the PdZn@Pb_0.25_/SiO_2_ catalyst suggest that the benefits of high selectivity could outweigh the disadvantage of a lower conversion rate.

In summary, trimetallic catalysts have been successfully applied to a variety of hydrogenation reactions, but because of the recent nature of these discoveries, the number of publications on this subject remains relatively low. The work that has been published to date highlights the importance of continued research into the area and of the balance between selectivity and reaction rate in designing catalysts. The progression to trimetallic catalysts for use in hydrogenation reactions has been shown to improve catalytic performance compared with bimetallic catalysts, particularly for hydrogenation of carbon monoxide and carbon dioxide. Often the third metal can enhance the selectivity through reduction of overhydrogenation, such as in the direct synthesis of H_2_O_2_ or alkyne hydrogenation.

### 4.2 Dehydrogenation Reactions

Dehydrogenation reactions involve the removal or liberation of hydrogen from a molecule. At present, many commercial dehydrogenation reactions are practiced. The dehydrogenations of molecules such as formic acid^220−223^ and ammonia borane^224−226^ are of high importance, as are dehydrogenations of alkanes to alkenes^227−229^ or alkynes.^230−232^ In finding cleaner and more sustainable energy sources, dehydrogenation reactions have become important in the realization of hydrogen storage for energy, including the specific examples of harvesting stored hydrogen from simple molecules such as formic acid and ammonia borane. Similar to hydrogenation reactions, heterogeneous catalysts (often Pt- or Pd-based) are important for obtaining high activities and selectivities under milder conditions.^233−235^ The following section details recent examples of the use of TMNPs in various dehydrogenation reactions.

#### 4.2.1 Dehydrogenation of Formic Acid to Hydrogen and Carbon Dioxide

The dehydrogenation of formic acid (FA) to give H_2_ and CO_2_ has been widely studied because of the interest in use of FA for hydrogen storage and potential energy generation.^236,237^ There have been extensive studies investigating modifications of the commercially used Pd/C catalyst for this dehydrogenation reaction.^238^ As a reference point, Hu *et al.* characterized and evaluated a commercial Pd/C catalyst and reported a TOF of >500 h^–1^ at 333 K in aqueous solution.^239^ Multiple supports have also been investigated, and high TOFs have been observed over catalysts using CeO_2_, N-functionalized porous carbon (N-MSC-30), and reduced graphene oxide, for example.^240,241^

Enhanced catalytic performance for formic acid dehydrogenation has been observed through the use of multiple metals in bimetallic and trimetallic systems. For example, the addition of Ag to Pd/C showed a significant improvement in activity; as an example, Zhang *et al.*(242) prepared a AgPd/C catalyst *via* a coreduction method, and the Ag_42_Pd_58_ alloy catalyst exhibited a TOF of 382 h^–1^ at 323 K. The enhancement in catalytic performance compared with the as-synthesized monometallic counterparts was ascribed to the charge transfer between Ag and the active Pd center due to the net difference in work function.^238^

**Figure 15:**
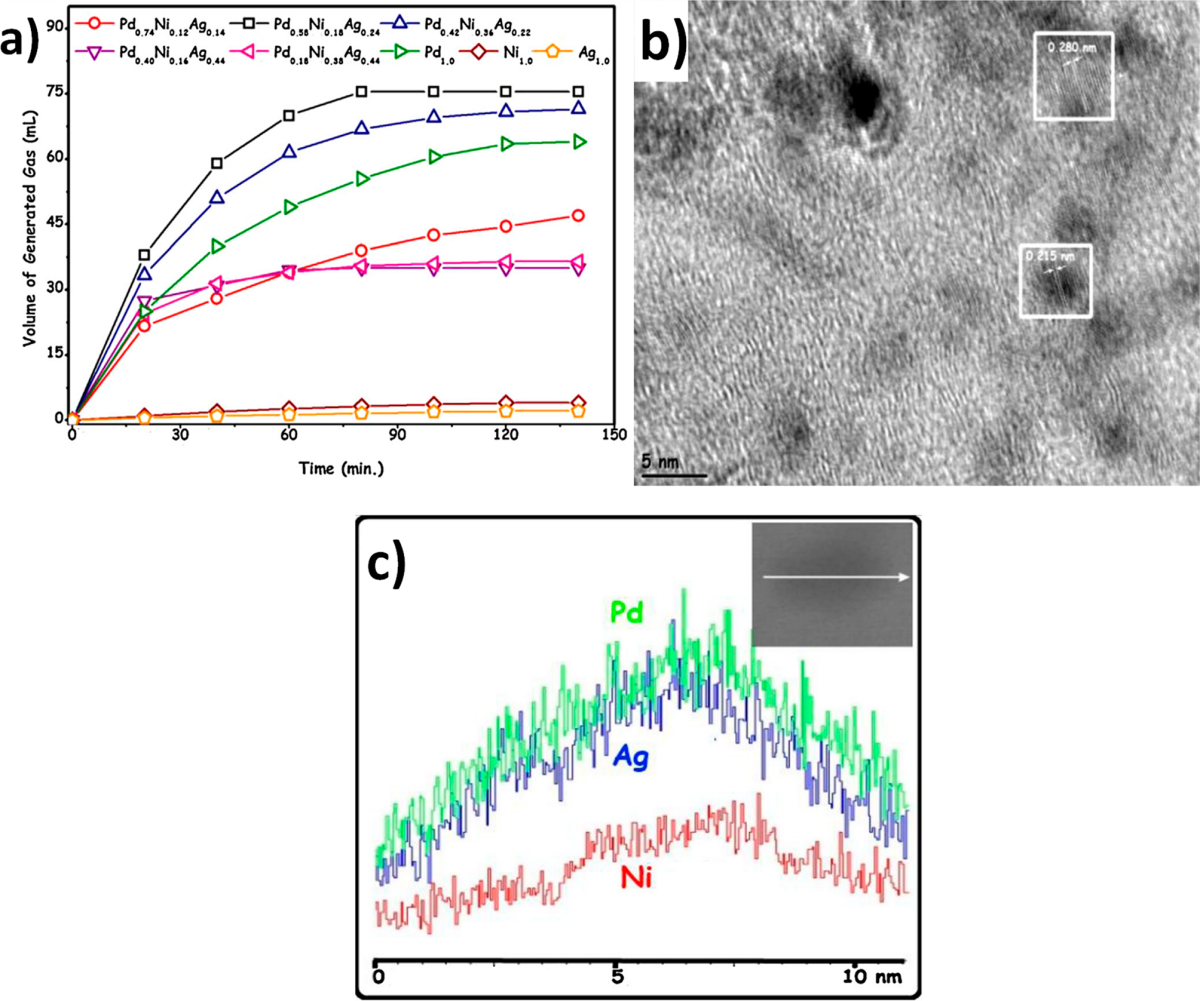
(a) Volumes of H_2_ gas generated as functions of reaction time from the dehydrogenation of FA over MMNP (Pd/C, Ag/C, Ni/C), BMNP (Pd_0.55_ Ni_0.45_/C, Pd_0.52_ Ag_0.48_/C, Ni_0.58_Ag_0.42_/C), and TMNP (Pd_0.74_Ni_0.12_Ag_0.14_/C, Pd _0.42_Ni_0.36_Ag_0.22_/C, Pd_0.40_Ni_0.16_Ag_0.44_/C, Pd_0.18_Ni_0.38_Ag_0.44_/C) catalysts. Reaction conditions: [metal] = 2.85 mM and [FA] = [SF] = 0.175 M in 10.0 mL of aqueous solution at 323 K. (b) HRTEM images of PdNiAg/C. (c) Elemental distribution of components in the PdNiAg NPs obtained by line-scan analysis using STEM-EDX along the white arrow in the HAADF-STEM image of PdNiAg/C given in the inset. Adapted with permission from ref (27). Copyright 2014 Elsevier.

Yurderi *et al.* prepared trimetallic PdNiAg NPs supported on activated carbon *via* wet impregnation followed by simultaneous reduction in the absence of stabilizers.^27^ The dehydrogenation of FA was carried out over a series of mono-, bi-, and trimetallic NP catalysts, and the highest activity was observed over the catalyst composed of Pd_0.58_Ni_0.18_Ag_0.24_ at 323 K (TOF = 85 h^–1^), with a high selectivity of *ca.* 100% (Figure 15a). The supported TMNPs were reported to be well-dispersed, with typical particle diameters of 2.5–8.4 nm, and alloyed, as supported by powder XRD, TEM, STEM-EDX mapping, and HAADF-STEM elemental mapping (Figure 15b,c). In the same study, the TMNPs provided higher catalytic activity than the monometallic counterparts prepared by the same method.^27^ The high activities were explained by the combination of (i) the composition of the trimetallic catalyst, (ii) low activation energies, and (iii) the synergistic effects from a change in the electronic density of Pd resulting from PdNiAg alloy formation. Additionally, the TMNP catalyst showed significant stability, maintaining activity (>94%) after four reusability tests. From ICP-OES data, the PdNiAg/C catalyst exhibited high stability against leaching, as no leaching was observed.^27^

**Figure 16:**
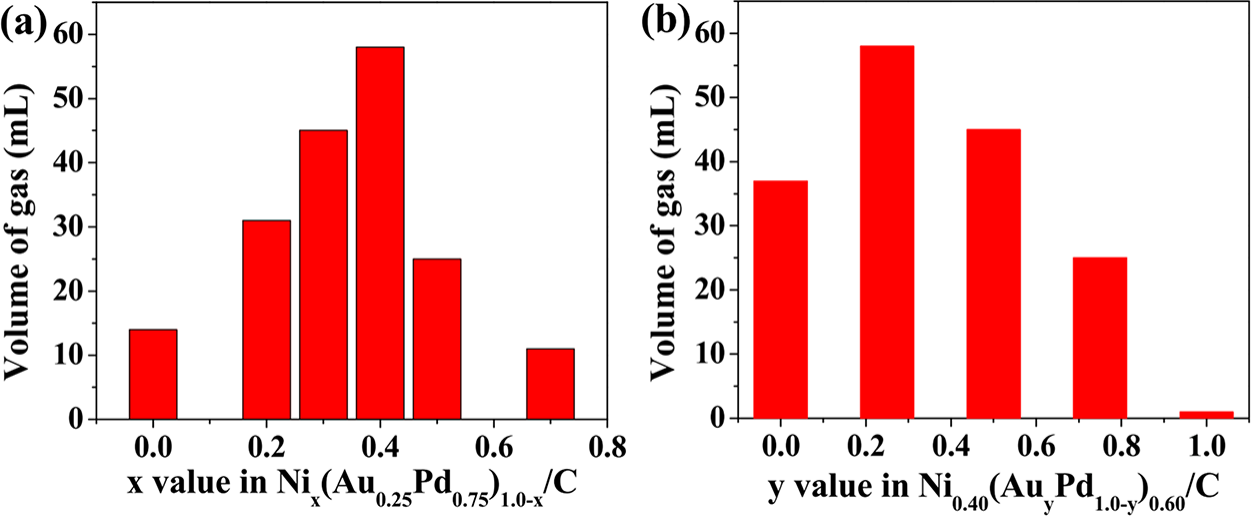
Illustration of the catalytic activity as a function of composition of TMNPs through measurements of gas generation from the dehydrogenation of FA (0.5 M, 10.0 mL) catalyzed by (a) Ni_*x*_(Au_0.25_Pd_0.75_)_1.0–*x*_/C with different *x* values and (b) Ni_0.40_(Au_*y*_Pd_1.0–*y*_)_0.60_/C with different *y* values at 298 K under ambient atmosphere (*n*_metal_/*n*_FA_ = 0.02, reaction time = 1 h). Adapted with permission from ref (77). Copyright 2014 Elsevier.

Wang *et al.* synthesized NiAuPd/C catalysts by a coreduction method in the absence of a surfactant and applied the catalysts to the dehydrogenation of FA.^77^ In comparison with the study reported by Yurderi *et al.*,^27^ larger TMNP diameters were formed *via* this method (16–35 nm), and an initial TOF of 12.4 h^–1^ at 298 K over Ni_0.40_Au_0.15_Pd_0.45_/C was reported, representing an increase in performance compared with the as-synthesized mono- and bimetallic counterparts. The enhanced activity may be ascribed to the synergistic effects that arise through the modification of the electronic structure of the NPs, which lead to different interactions between the catalytic surface and FA. In the example considered here, monometallic Pd/C showed low activity and is known to be susceptible to deactivation through CO poisoning.^239,243^ Additionally, comparison of the diffraction peaks for NiAuPd NPs to monometallic Au showed a shift to a lower diffraction angle for TMNPs, indicating an increase in the crystal lattice spacing from the formation of a NiAuPd alloy. The study also noted a strong correlation between composition and activity, with the volume of the evolved gas (1 h reaction time) correlated positively with the increase in Ni content to an optimized ratio. As a result, Wang *et al.* concluded the best composition to be Ni_0.40_Au_0.15_Pd_0.45_/C.^77^ The catalytic activity as a function of composition (Figure 16) highlights the need to optimize the respective metal concentrations of the active site.

**Figure 17:**
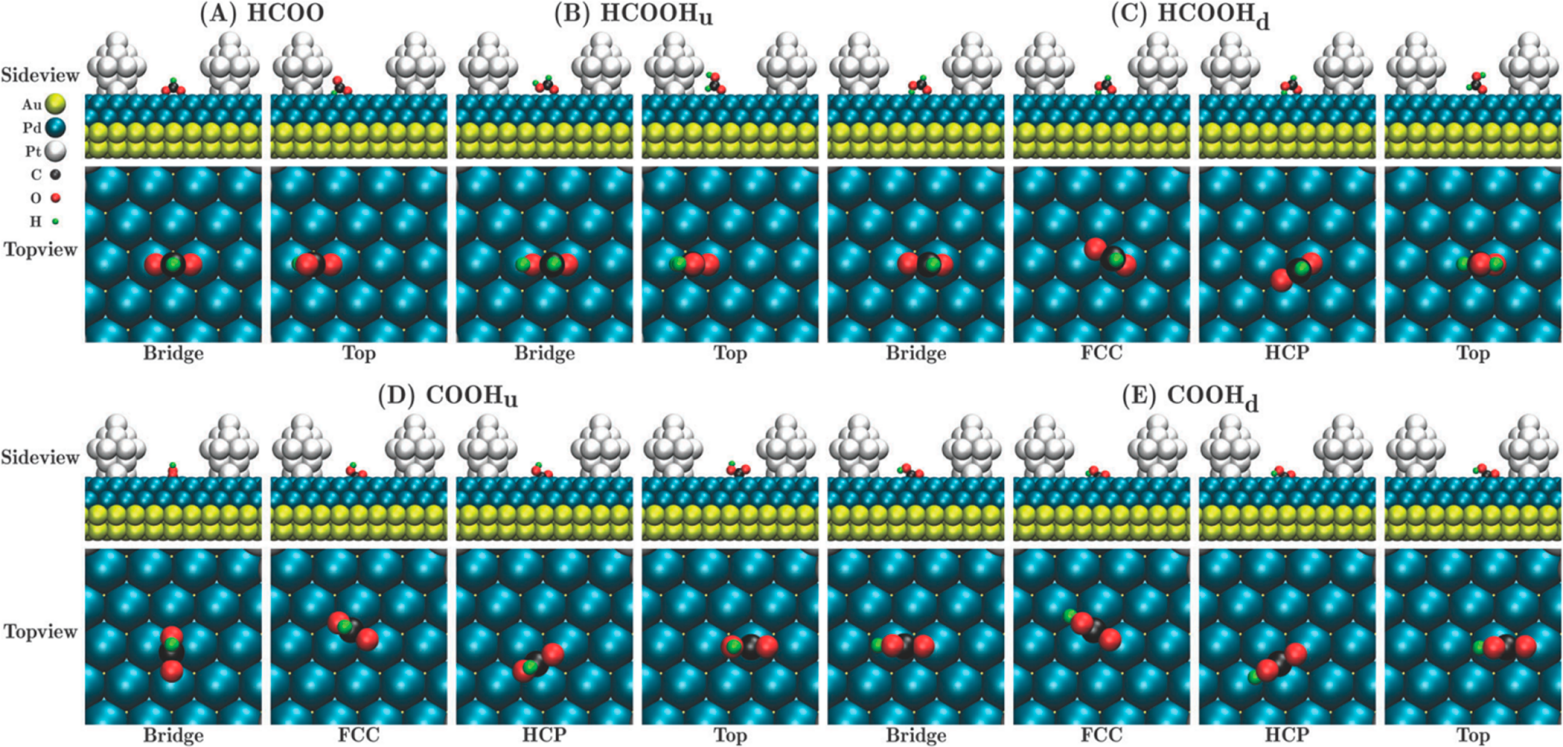
Optimized structures of (A) HCOO, (B) HCOOH_u_, (C) HCOOH_d_, (D) COOH_u_, and (E) COOH_d_ adsorbed on different sites in the center area of Au@2 ML Pd@0.5 ML Pt NPs. A key is provided at the top left. Adapted with permission from ref (245). Copyright 2013 The PCCP Owner Societies.

Fang *et al.* used DFT calculations to investigate Au_(core)_Pd_(shell)_Pt_(cluster)_ TMNPs for oxidation of CO and FA. CO adsorption is rare on “two Pd atoms between two Pt clusters mixed-hollow sites”, termed D-sites, which remain free to catalyze the electrooxidation of FA, leading to the high catalytic activity. The optimum Pt coverage on Pd was θ_Pt_ ≈ 0.5, thus maximizing the number of mixed-hollow D-sites and promoting the catalytic reactivity. The catalytic activity was observed to decrease with increasing thickness of the Pd overlayer, specifically when the thickness was greater than two monolayers. The authors proposed that the Au core can be replaced by less expensive Ag, since Au is recognized as providing structure morphology and not an electronic contribution;^244^ such a concept could be potentially extended to other low-cost, stable transition metals. Duan *et al.*(245) used computational methods to investigate nine possible decomposition routes of HCOOH on analogous Au_(core)_Pd_(shell)_Pt_(cluster)_ TMNPs, considering a variety of different adsorption sites, as shown in Figure 17.

**Figure 18:**
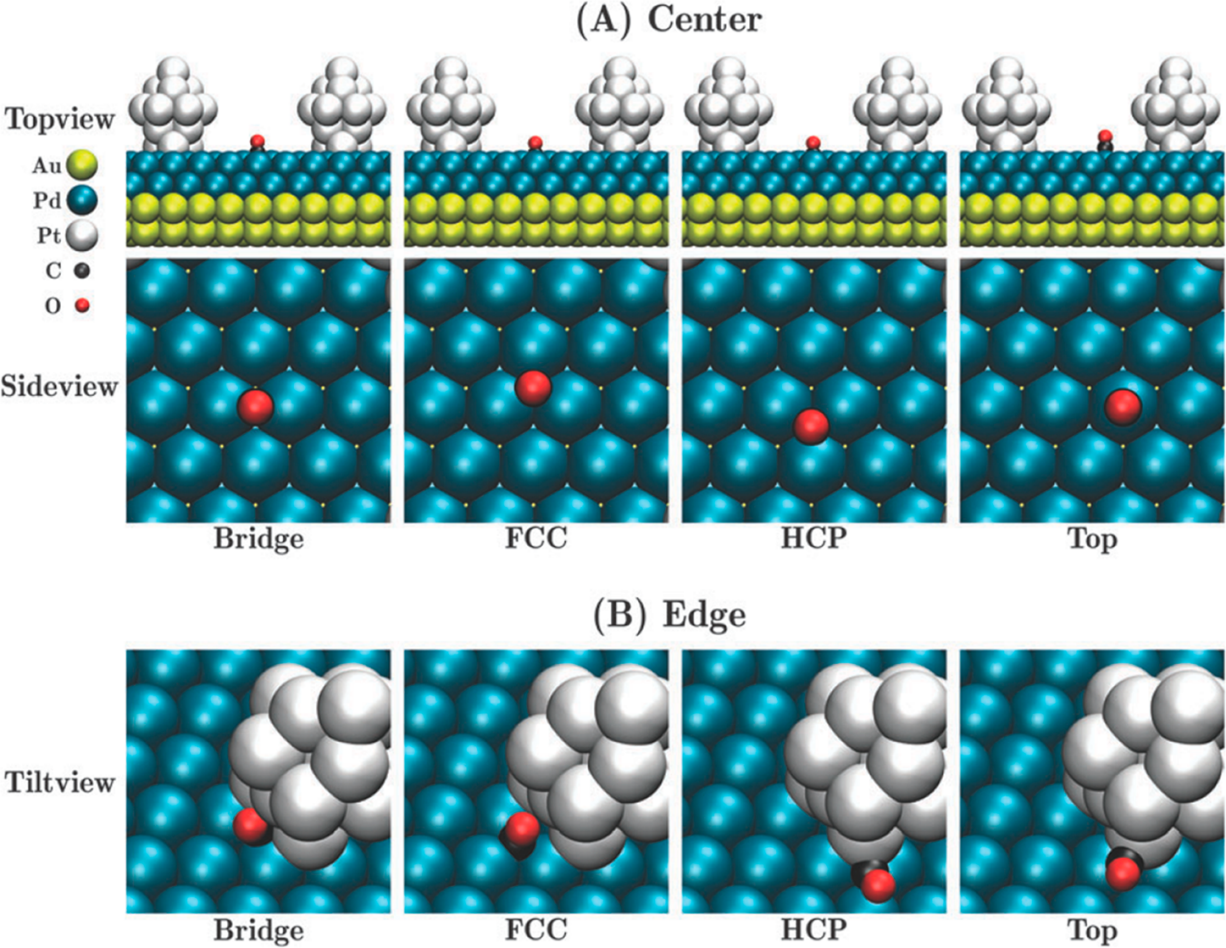
Schematic adsorption areas at (A) the center of a Pd facet and (B) the edge of a Pd/Pt moiety for various adsorption sites (*i.e.*, bridge, FCC, HCP, and top from left to right) and optimized structures of CO adsorbed on Au@2 ML Pd@0.5 ML Pt NPs. A key is provided in the top left corner. Adapted with permission from ref (245). Copyright 2013 The PCCP Owner Societies.

CO, which may poison the catalyst, was repelled by the Pt/Pd edge site, as illustrated in Figure 18. The repulsion of this poisonous species contributed to the high electrocatalytic performance by effectively leaving the site vacant for the CO_2_ pathway, which itself was simultaneously enhanced in the edge area. H and O atoms were shown to prefer to adsorb on the FCC and HCP sites in the center area of the Pt/Pd surface on Au@2 ML Pd@0.5 ML Pt NPs (core@shell@shell configuration).

Wu *et al.* also investigated dehydrogenation of formic acid using TMNPs, with the intent of generating H_2_*in situ* for the hydrogenation of nitrobenzene to aniline.^26^ In the chemical industry, aniline is an important intermediate^246^ for dyes,^247^ agricultural chemicals,^248^ and pharmaceuticals.^249^ These catalysts were synthesized using the defect-dominated shape recovery method to fine-tune the stoichiometry of the nanoparticle. Among the Pt_3_Ni-based catalysts and stoichiometries prepared, Pt_3_Ni@Au_0.5_ was reported to have the highest activity and selectivity toward the formation of aniline, with a yield of 97% and a TOF of 124 h^–1^, which is reportedly a 10-fold increase over bimetallic Pt_3_Ni, for which the TOF was calculated to be 12 h^–1^. An increase in gold content resulted in a loss of activity, with the TOF of Pt_3_Ni@Au_2_ diminishing to 84 h^–1^. The activity further diminished to merely 6 h^–1^ with the fully octahedral and defect-free nanoparticle Pt_3_Ni@Au_8_.

A loss of activity with increasing metal coverage was also observed when Ag, Cu, and Rh were used as the third metal for the same systems, which supports the postulate that defect sites exhibit catalytic properties, as increasing metal coverage corresponds to a loss of defect sites; however, the identity of the third metal remains important. The activities of catalysts where the third metal was Ag, Cu, or Rh were far lower than for Au as the third metal. The electronic properties of Pt_3_Ni@Au_*x*_ were investigated; reported electronic coupling within the structure indicated electron transfer from Au to Pt,^250^ thereby effectively reducing Pt(II) within the nanoparticle to a lower and more catalytically active oxidation state. Conversely, Au and Ni are slightly oxidized. The TOF of 124 h^–1^ obtained for the Pt_3_Ni@Au_0.5_ composition was considerably greater than the TOF of 85 h^–1^ obtained with the PdNiAg/C catalyst reported by Yurderi *et al.*,^27^ which may in part be due to the 20 K higher temperature used by Wu *et al.* or the considerably smaller nanoparticle size.

Using a unique support, Mori *et al.*(251) prepared PdCuCr TMNPs within a macroreticular basic resin. The effect of the slightly basic resin was to assist with O–H cleavage, aiding the dehydrogenation of FA. At 348 K, this catalyst exhibited a high TOF of 830 h^–1^, which was substantially higher than those over bimetallic PdCu and PdCr catalysts and monometallic Pd. The addition of Cr was thought to stabilize the PdCu NPs against agglomeration and to enrich the Pd center with electrons, which are beneficial synergistic effects that achieve higher catalytic performances.

Khan^252^ demonstrated that TMNPs with the same elemental composition created by different synthetic methods can lead to different outcomes with regard to both particle morphology and catalytic activity. Using three different orders of metal introduction, Khan synthesized different unsupported core@shell TMNP structures consisting of Pd, Ag, and Fe. The ratio of the metals in each experiment was fixed at 1:1:1, and each TMNP had an M1M2@M3 structure. These catalysts were then applied to the degradation of formic acid to carbon dioxide and hydrogen gas.

First, unsupported PdAg@Fe TMNPs were synthesized using a seedless ligand-capped reduction method with CTAB as the capping agent and hydrazine as the reducing agent. In contrast, FePd@Ag TMNPs were synthesized through stepwise addition of the metals. The Fe precursor was reduced with hydrazine and CTAB, forming Fe^0^ MNPs. An aqueous Pd^2+^ solution was added to the Fe MNP suspension along with additional CTAB, depositing Pd onto the surface *via* a displacement reaction. The FePd BMNPs were isolated from the reaction mixture and dispersed in deionized water; repeating the procedure with an aqueous Ag^+^ solution in the FePd BMNP suspension then yielded FePd@Ag TMNPs. Finally, FeAg@Pd TMNPs were synthesized by simultaneously reducing Ag^+^ and Pd^2+^ onto Fe^0^ MNPs, and the final product was washed with copious amounts of deionized water to remove any CTAB. The different reduction potentials of the metal salt precursors were used to explain how these materials formed through the formation of a galvanic cell replacement reaction.^252^

Interestingly, the activity profiles for the dehydrogenation of formic acid over this series of catalysts were different. FeAg@Pd, with *k*_obs_ = 0.83 h^–1^ (TOF = 75 mol_H_2__ mol_cat_^–1^ h^–1^), had the highest activity, whereas over FePd@Ag and PdAg@Fe the observed *k*_obs_ values were calculated to be 0.54 and 0.33 h^–1^, respectively (Table 4).^252^ Over FeAg@Pd, the calculated *k*_obs_ corresponded to the formation of 88 mL of the (CO_2_ + H_2_) product in 120 min; in contrast, over monometallic Fe and Ag catalysts, 3 mL of the product was formed in 120 min. The activation energy over the FeAg@Pd catalyst was 51 kJ mol^–1^, compared with 60 and 66 kJ mol^–1^ over the FePd@Ag and PdAg@Fe catalysts, respectively. Although these rates were considerably lower than previous reports, these reactions were carried out at 303 K, whereas the reactions were carried out at >323 K as reported by other groups (Table 4). Overall, the report demonstrates how different preparation methods for TMNPs with the same composition can have a direct and stark impact on the catalytic outcome. This can be due to the different morphologies, sizes, and nanoparticle structures that can manifest even with the same elemental composition, depending on the method used.

**Table 4:** Summary of Catalysts for Dehydrogenation of Formic Acid

catalyst	catalyst type	reaction conditions	performance	ref
AgPd/C	supported BMNP	323 K	TOF = 382 h^–1^	(242)
Pd/C	spported MMNP		TOF = 75 h^–1^; volume of H_2_ generated after 140 min = 63 mL	(27)
Ni/C	supported MMNP		TOF = 4 h^–1^; volume of H_2_ generated after 140 min = 4 mL	
Ag/C	supported MMNP		TOF = 2 h^–1^; volume of H_2_ generated after 140 min = 2 mL	
Pd_0.58_Ni_0.18_Ag_0.24_/C	supported TMNP		TOF = 85 mol_H_2__ mol_cat_^–1^ h^–1^; volume of H_2_ generated after 140 min = 76 mL	
Pd/C	supported MMNP	*T* = 298 K; *p* = 0.1 MPa	TOF = 8.3 h^–1^; formic acid conversion after 600 min = 39%	(77)
Ni/C	supported MMNP		formic acid conversion after 600 min = 2%	
Au/C	supported MMNP		formic acid conversion after 600 min = 0%	
Ni_0.4_Pd_0.6_/C	supported BMNP		TOF = 8.4 h^–1^; formic acid conversion after 600 min = 52%	
Au_0.25_Pd_0.75_/C	supported BMNP		TOF = 10.1 h^–1^; formic acid conversion after 600 min = 42%	
Ni_0.4_Au_0.6_/C	supported BMNP		formic acid conversion after 600 min = 1%	
Ni_0.4_Au_0.15_Pd_0.45_/C	supported TMNP		TOF = 12.4 h^–1^; formic acid conversion after 600 min = 73%	
physical mixture of Ni/C, Au/C, and Pd/C (Ni:Au:Pd = 0.4:0.15:0.45)	mixture of MMNPs		TOF = 3.7 h^–1^; formic acid conversion after 600 min = 22%	
PdAg@Fe	core@shell TMNP	303 K	*k*_obs_ = 0.328 h^–1^ (TOF = 30 mol_H_2__ mol_cat_^–1^ h^–1^)	(252)
FePd@Ag	core@shell TMNP		*k*_obs_ = 0.540 h^–1^ (TOF = 49 mol_H_2__ mol_cat_^–1^ h^–1^)	
FeAg@Pd	core@shell TMNP		*k*_obs_ = 0.828 h^–1^; CO_2_ + H_2_ generated after 120 min = 88 mL; TOF = 75 mol_H_2__ mol_cat_^–1^ h^–1^	
PdCuCr	TMNP constructed *in situ*	348 K; HCOOH/HCOONa = 9:1 aqueous solution	H_2_ production rate = 174 000 mL h^–1^ g_Pd_^–1^; TOF = 830 h^–1^	(251)

The emerging reports of TMNPs as catalysts for the dehydrogenation of FA have in part been inspired by the improved catalytic performances observed with bimetallic catalysts for this reaction. In combination with other enhancements, such as using different supports, further improvements can be achieved by trimetallic systems that will improve outcomes compared with the commercial Pd/C catalyst. The optimized electronic character of the NPs is commonly ascribed to the enhanced activity, and this is where optimizing the metal composition can be exploited with TMNPs.

#### 4.2.2 Dehydrogenation of Alkanes to Alkenes

When technology for the dehydrogenation of alkanes to alkenes was first developed in the late 1930s, chromia–alumina catalysts were used. These were mainly employed to produce butenes from butane, which could then be upgraded to more highly valued octenes and octanes. In the 1960s, Bloch identified that platinum-based catalysts could be used as highly active and stable catalysts to selectively dehydrogenate heavy linear alkanes to internal monoalkenes.^253^ As of 2014, the global production of propene from the dehydrogenation of propane was 5 million tons, and this is set to increase with at least 12 new propane dehydrogenation plants planned to be commisioned.^254^ There are two patented industrial processes for the dehydrogenation of propane, called the Oleflex process and the Catofin process, which use Pt–Sn/Al_2_O_3_ and Cr_2_O_3_/Al_2_O_3_ catalysts, respectively.

To improve on the Oleflex commercial formulation, the support influence of pure alumina, pure zirconia, and different percentage mixtures of zirconia and alumina (χZr–Al, where χ represents the mass percentage of ZrO_2_) on PtSnIn-based trimetallic catalysts was assessed.^128^ The supports were prepared by precipitation/coprecipitation, and the metals were then added by sequential impregnation.

The performance of the catalysts for propane dehydrogenation was assessed by measuring the propene selectivity, the initial and final propane conversion, and the decline in propane conversion over a 2.5 h reaction cycle. The propane conversions, both initial and final, decreased in the order PtSnIn/08Zr–Al > PtSnIn/04Zr–Al > PtSnIn/16Zr–Al > PtSnIn/Al_2_O_3_ ≫ PtSnIn/ZrO_2_. The results of these catalytic studies have been collated in Table 5 for comparison. The PtSnIn/08Zr–Al catalyst also had the highest propene selectivity (>98%). Furthermore, the PtSnIn/08Zr–Al catalyst was proven to be very stable, showing only a 3% decrease in initial conversion over five 2.5 h reaction cycles.

**Table 5:** Comparison of Catalysts Containing Pt, Sn, and In for the Dehydrogenation of Propane to Propene

			conversion/% (selectivity/%)		
catalyst	catalyst type	reaction conditions	initial	≥150 min	ref
PtSn/In-Al_2_O_3_	supported TMNP	*T* = 873 K; *p* = 0.1 MPa; H_2_:C_3_H_8_:Ar molar ratio = 7:8:35; WHSV = 3.3 h^–1^; *m*_cat_ = 0.3 g	47.0 (96.6)	36.0 (97.1)	(128)
PtSnIn/ZrO_2_			22.0 (96.1)	15.0 (95.9)	
PtSnIn/04Zr–Al_2_O_3_			54.8 (98.0)	47.6 (98.8)	
PtSnIn/08Zr–Al_2_O_3_			57.7 (98.3)	51.7 (98.7)	
PtSnIn/16Zr–Al_2_O_3_			51.2 (97.2)	45.8 (98.6)	
PtSn/In-Al_2_O_3_	supported TMNP	*T* = 873 K; H_2_:C_3_H_8_:Ar molar ratio = 7:8:35; WHSV = 3.3 h^–1^; *m*_cat_ = 0.3 g	62.0 (96.4)	37.0 (97.3)^a^	(127)
PtSnIn/0.6Ca–Al_2_O_3_			62.0 (96.8)	54.0 (97.6)^a^	
PtSnIn/1.2Ca–Al_2_O_3_			60.0 (97.0)	57.5 (97.8)^a^	
PtSnIn/1.5Ca–Al_2_O_3_			62.0 (97.6)	58.0 (97.8)^a^	
PtSnIn/1.8Ca–Al_2_O_3_			54.5 (96.6)	49.0 (96.25)^a^	
PtSnIn/2.4Ca–Al_2_O_3_			50.0 (96.5)	34.5 (96.25)^a^	
In/Al_2_O_3_	supported MMNP	*T* = 893 K; H_2_:C_3_H_8_:Ar molar ratio = 7:8:35; GHSV = 1500 h^–1^; *m*_cat_ = 0.3 g	10.0 (44.0)	9.5 (58.5)^b^	(255)
Pt/Al_2_O_3_			29.5 (55.5)	15.5 (67.0)^b^	
PtSn/Al_2_O_3_	supported BMNP		37.5 (83.0)	23.5 (86.0)^b^	
PtIn/Al_2_O_3_			53.0 (93.5)	37.0 (92.0)^b^	
PtSn/In-Al_2_O_3_	supported TMNP		58.0 (92.5)	48.0 (93.5)^b^	

a After 22 h.

b After 2.75 h.

Further examination revealed that the poor performance of the pure-zirconia-supported catalyst for propane dehydrogenation was probably due to its negligible surface acidity and low surface area. The ZrO_2_ was also found to obscure some of the Pt particles through a strong metal–support interaction, which resulted in a zirconia suboxide species decorating the surface of the Pt and obscuring its active sites. The PtSnIn/χZr–Al catalysts, however, performed well because of the moderate surface acidity arising from the high percentage of weak and medium-strength acid sites. These catalysts also have the smallest particle size and most homogeneous dispersion of metal. The χZr–Al mixture can promote the reduction of Pt species and stabilize the Sn oxidation states, which helps to maintain the catalyst stability. Assessment of the catalysts through C_3_H_6_ TPD showed that the reduction temperature of propene decreased in the order PtSnIn/ZrO_2_ > PtSnIn/Al_2_O_3_ > PtSnIn/08Zr–Al. The reduction temperatures show that the combination of ZrO_2_ and Al_2_O_3_ weakens the propene–support interaction compared with the other supports, and hence, the product is more easily removed from the catalyst before it reacts further to give higher-order dehydrogenation products, ensuring high selectivity.

In an earlier study, the same group investigated doping of γ-Al_2_O_3_-supported PtSnIn catalysts with calcium.^127^ As propane dehydrogenation is an endothermic reaction, high temperatures are usually employed, leading to rapid catalyst deactivation and poor selectivity due to the formation of coke and cracking. Ca has been shown to improve the stability of the catalyst and metal NP dispersion in bimetallic PtSn catalysts, resulting in improved performance. Long *et al.* prepared a range of PtSnIn/χCa–Al catalysts (where χ represents the mass percentage of Ca) by sequential impregnation. The initial conversions of propane over PtSnIn supported on 0.0Ca–Al, 0.6Ca–Al, 1.2Ca–Al, and 1.5Ca–Al were similar at *ca.* 60%; however, the final conversions were 36.7%, 54.0%, 57.0%, and 58.9%, respectively, indicating how the surface concentration of Ca affects the efficacy of the catalyst as a function of reaction time. The most stable catalyst was PtSnIn/1.5Ca–Al, which exhibited just a 1.2% decline in propane conversion over a 22 h reaction cycle; however, as the Ca content was increased above 1.5 wt %, the propane conversion and catalyst efficacy decreased. The PtSnIn/1.8Ca–Al catalyst showed an initial conversion of 54.6% and a final conversion of 49.5% after a 22 h reaction cycle, and the PtSnIn/2.4Ca–Al catalyst exhibited an initial conversion of 50.0% and a final conversion of 34.5%. Furthermore, over both of these catalysts the selectivity for propene was below 97%, whereas the catalysts with <1.5 wt % Ca had a propene selectivity of over 97.5%.

This study suggests that among all of the Ca-doped catalysts tested, the ideal catalyst for propane dehydrogenation was PtSnIn/1.5Ca–Al, which showed the least decline in propane conversion during time on stream and a high selectivity of over 98.0%. For this catalyst, the initial propane conversion decreased from 60.1% to 58.9% after 22 h on stream, which is a minimal decrease of 1.2%. After 100 h, the propane conversion had decreased by a further 24.9–34.0%, but this represents much higher stability than catalysts with other Ca concentrations. The reason given for the improved performance is that Ca introduces a bifunctional effect by providing acidic active sites on the support in addition to the metal active sites in the nanoparticles. These two kinds of active sites work synergistically and as such exist in an optimum ratio. The alkaline nature of Ca means that it neutralizes the otherwise strongly acidic support sites on the alumina, reducing negative effects caused by isomerization and coking that are prevalent on strongly acidic sites; however, if the concentration of Ca is too high, the optimum ratio of metal sites to acidic support sites is lost.

Comparison of these two studies by Long *et al.*(127,128) (Table 5) shows that the PtSnIn/1.5Ca–Al catalyst displayed the best activity and selectivity, with an initial propane conversion of 60.1%, which decreased by 0.8% to 59.3% after 2.5 h. This is a higher conversion and smaller decrease than observed over the PtSnIn/08Zr–Al catalyst, which had an initial propane conversion of 57.7% that decreased by 6.0% to 51.7% after 2.5 h on stream. However, the PtSnIn/08Zr–Al catalyst was more selective for the desired product, propene, with an initial selectivity of 98.3% that increased to 98.6% after 2.5 h; comparatively, over the PtSnIn/1.5Ca–Al catalyst the initial propene selectivity of 96.6% increased to 97.0% after 2.5 h.

The addition of a third metal in propane dehydrogenation catalysts has been shown to have beneficial effects. Long *et al.*(255) tested trimetallic PtSnIn catalysts, alongside monometallic Pt and bimetallic PtSn, supported on γ-Al_2_O_3_. Again, an impregnation method was used to produce the catalyst. The study showed that the addition of a third metal, indium, to a bimetallic PtSn catalyst resulted in improved catalytic performance and stability because the In helps to neutralize the strongly acidic sites on the γ-Al_2_O_3_ surface, enabling higher selectivity toward propene. After a 53 h reaction cycle, the final propane conversion was >41.0% and the selectivity remained stable at >96% at 873 K. The dilution effect caused by adding an additional metal also facilitated a reduction of the particle dimensions, with the average diameter of the PtSnIn TMNPs being 10.8 nm, compared with 17.9 nm for monometallic Pt/Al_2_O_3_ and 12.1 nm for bimetallic PtSn/Al_2_O_3_.

To compare monometallic, bimetallic, and trimetallic catalysts for the propane dehydrogenation reaction, an experiment was run over 2.75 h with monometallic In/Al_2_O_3_ and Pt/Al_2_O_3_ catalysts, bimetallic PtSn/Al_2_O_3_ and PtIn/Al_2_O_3_ catalysts, and trimetallic PtSn/In-Al_2_O_3_. Under the reaction conditions of 893 K and GHSV = 1500 h^–1^, the results outlined in Tables 5 and 6 were obtained. These results highlight the advantages of a TMNP catalyst for the propane dehydrogenation reaction, as the propane conversion decreased less than with the BMNP catalysts during the experiment and the propene selectivity was high, modestly improving to 93.5% during the test.

**Table 6:** Comparison of the Performances of Different Catalysts for Propane Dehydrogenation at 2.75 h Time on Line^a^^,^^255^

catalyst	*X*_I_ (%)	*X*_f_ (%)	±*X* (%)	*S*_I_ (%)	*S*_f_ (%)	±*S* (%)
In/Al_2_O_3_	10.0	9.5	–0.5	44.0	58.5	+14.5
Pt/Al_2_O_3_	29.5	15.5	–14.0	55.5	67.0	+11.5
PtSn/Al_2_O_3_	37.5	23.5	–14.0	83.0	86.0	+3.0
PtIn/Al_2_O_3_	53.0	37.0	–16.0	93.5	92.0	–1.5
PtSn/In-Al_2_O_3_	58.0	48.0	–10.0	92.5	93.5	+1.0

a *X*_i_ and X_f_ are the initial and final propane conversions, respectively, and ±*X* is the change in conversion over the reaction cycle. *S*_i_ and *S*_f_ are the initial and final propene selectivities respectively, and ±*S* is the change in selectivity over the reaction cycle.

#### 4.2.3 Dehydrogenation of Ammonia Borane

Ammonia borane is considered to be a suitable material for hydrogen storage because of its high hydrogen storage capacity, air stability, nonflammability, and low toxicity.^256,257^ The key driver for this technology is to efficiently dehydrogenate ammonia borane and release molecular hydrogen; consequently, many catalyst formulations have been evaluated, including those with TMNPs.

**Figure 19:**
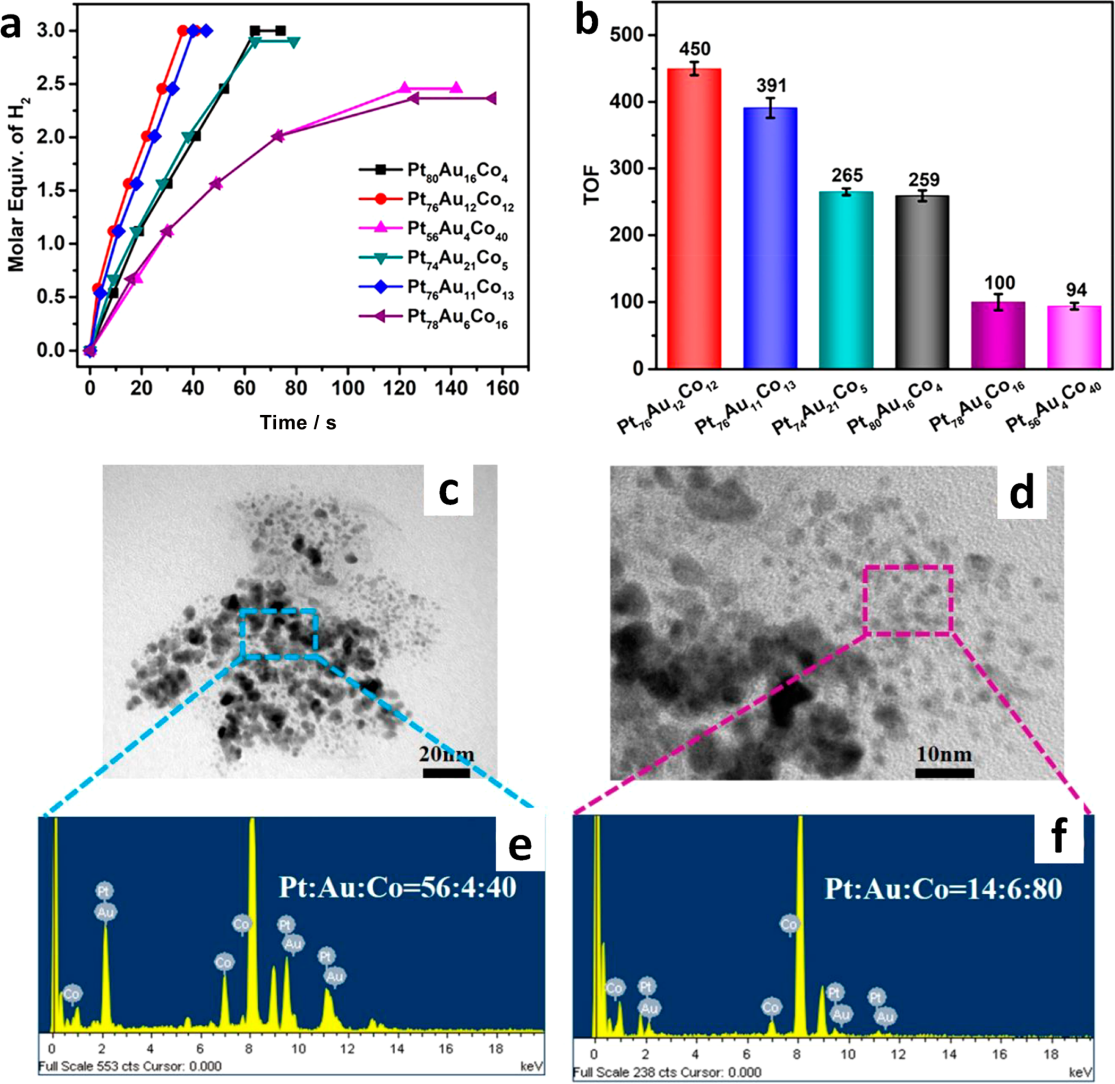
(a) Plots of H_2_ generation vs time and (b) the corresponding TOFs (mol_H_2__ mol_M_^–1^ min^–1^) for the ammonia borane hydrolysis reaction at 298 K catalyzed by trimetallic Pt–Au–Co catalysts with different compositions. (c, d) TEM images of Pt_56_Au_4_Co_40_ obtained after refluxing for 1 h and (e, f) the corresponding EDX data indicating the segregation of elements present due to high Co concentration, causing a reduction of catalytic activity. Adapted from ref (78). Copyright 2020 American Chemical Society.

Randomly alloyed PtAuCo catalysts prepared by Fu *et al.* were tested for the dehydrogenation of ammonia borane (Figure 19).^78^ Several ratios of PtAuCo were synthesized and tested: Pt_76_Au_12_Co_12_, Pt_76_Au_11_Co_13_, Pt_74_Au_21_Co_5_, Pt_80_Au_16_Co_4_, Pt_78_Au_6_Co_16_, and Pt_56_Au_4_Co_40_. The performance was compared to that of Pt_85_Au_15_ and Pt_86_Co_14_ bimetallic alloys, core@shell Au@Co, and monometallic Pt, Au, and Co. The TOF (moles of hydrogen formed per mole of metal per hour) obtained for each catalyst is displayed in Table 7, and these values are compared with those obtained over other catalyst formulations discussed here.

**Table 7:** Catalytic Performance of a Variety of Supported and Unsupported TMNP, BMNP, and MMNP Formulations for the Ammonia Borane Dehydrogenation Reaction

catalyst	catalyst type	reaction temperature (K)	TOF (h^–1^)	ref
Pt_76_Au_12_Co_12_	TMNP alloy	298	27000	(78)
Pt_76_Au_11_Co_13_			23460	
Pt_74_Au_21_Co_5_			15900	
Pt_80_Au_16_Co_4_			15540	
Pt_78_Au_6_Co_16_			6000	
Pt_56_Au_4_Co_40_			5640	
Pt_85_Au_15_	BMNP alloy		8220	
Pt_86_Co_14_			3960	
Au@Co	BMNP core@shell		840	
Pt	MMNP		240	
Au			180	
Co			120	
Pt (black)	MMNP	298	840	(258)
Ru@Co	BMNP core@shell	298	20640	(259)
AuCo/CN	supported BMNP	303	2880	(260)
PtCo	BMNP	293	18180	(261)
PtCu	BMNP	298	6480	(262)
Cu_40_Co_60_/BN	supported BMNP	298	480	(263)
PtCoCu@SiO_2_	protected TMNP	303	16380	(264)
Cu_72_Co_18_Mo_10_	TMNP	298	7140	(265)
CuNiCo/MOF	supported TMNP	298	4200	(266)
RuCuNi/CNTs	supported TMNP	298	18669	(28)

The TOFs obtained over the catalysts prepared by Fu *et al.*(78) varied greatly according to the stoichiometry of the three metals; however, the Pt_76_Au_12_Co_12_ composition proved to be superior to the other catalysts in the study, with a TOF of 27 000 h^–1^. By comparison, the most active BMNP formulation within this study was Pt_85_Au_15_, with a TOF of 8220 h^–1^, which is less than one-third of the reported TOF of Pt_76_Au_12_Co_12_. A separate study by Cao *et al.*(259) found that unsupported Ru@Co core@shell NPs are a more active BMNP formulation than Pt_85_Au_15_, with a TOF of 20 640 min^–1^; however, this turnover is still outperformed by the Pt_76_Au_12_Co_12_ composition. The performance of the Pt, Au, and Co MMNPs was relatively poor, with TOFs of 120–240 h^–1^, which are 2 orders of magnitude lower than those of the most active catalysts.

Analysis of XPS, TEM, and EDX characterizations of the PtAuCo catalysts indicated that as a result of a phase and size mismatch, a high concentration of Co caused segregation of the metals, as can be seen in TEM images of Pt_56_Au_4_Co_40_ (Figure 19), and this was correlated to a decrease in catalytic performance.^78^ Nanoparticle segregation occurred at high concentrations of Co, as can be seen for the high-cobalt composition (Pt_14_Au_6_Co_80_), where the majority of the MNP population were monometallic Co, with segregated Au/Pt islands. Similarly, Pt_78_Au_6_Co_16_ exhibited a small amount of Co segregation. The PtAuCo catalysts were assessed for their stability using two methods: first, another equivalent of ammonia borane solution was added following the cessation of H_2_ formation; second, the catalyst was recovered by centrifugation, washed with deionized water, and redispersed in a fresh ammonia borane solution. With both methods, the catalysts showed significant degradation of performance after five runs. The TOF of the Pt_76_Au_12_Co_12_ catalyst fell to 15 120 h^–1^ by the fifth run with the first method. The second method resulted in less degradation, with the TOF falling to 21 900 h^–1^ by the fifth experiment. The bimetallic Pt_85_Au_15_ composition exhibited a similar decline in TOF, falling from 8220 to 3900 h^–1^ by the fifth run with the first method. With the second method, the activity decreased modestly, to 5580 h^–1^. The second method proved to be superior to facilitate reusability, as the Pt_76_Au_12_Co_12_ retained 81% activity after five runs compared with 56% activity with the first method. The loss of activity was attributed to the production of BO_2_^–^ ions in the reaction, which increased the viscosity of the solution and slowed the diffusion, adsorption, and desorption processes.^267^

As Pt is the main component of many highly active catalysts for the dehydrogenation of ammonia borane, Pt likely provides the main active sites for Pt_76_Au_12_Co_12_. XPS measurements indicated that the Pt in the nanoalloys was slightly oxidized. The electronegativity of Pt is 2.20, whereas that of Au is 2.54 and that of Co is 1.70. The presence of Au results in a positive shift of the binding energy (BE), whereas Co results in a negative shift (Pt 4f BE = 71.34 eV for Pt_76_Au_12_Co_12_, 71.52 eV for Pt_85_Au_15_, and 71.25 eV for Pt_86_Co_14_). The data suggested that the intermediate binding energy of the Pt_76_Au_12_Co_12_ composition provided the optimal interaction energy between the catalyst and the active intermediates in the ammonia borane dehydrogenation reaction.

Other TMNP catalysts, which combine different metals than those already discussed, have been investigated for the dehydrogenation of ammonia borane. Highly dispersed, alloyed RuCuNi NPs were supported on carbon nanotubes (40–60 nm diameter) *via* a chemical reduction method and tested for ammonia borane dehydrogenation.^28^ Ru/CNTs, Cu/CNTs, Ni/CNTs, bimetallic RuCu/CNTs and RuNi/CNTs, and trimetallic RuCuNi/CNTs were prepared and then tested at room temperature. The resulting catalytic activity order was RuCuNi/CNTs > RuNi/CNTs > RuCu/CNTs > Ru/CNTs > Ni/CNTs > Cu/CNTs. The importance of alloy formation was investigated by Xiong *et al.* through comparison of RuCuNi/CNTs with physical mixtures of the as-prepared monometallic Ru/CNTs, Cu/CNTs, and Ni/CNTs, which showed inferior catalytic activity compared with the alloyed trimetallic catalyst. Overall, the study showed strong synergistic effects among the three alloyed metals, as manifested by the reported increase in catalytic activity for this reaction; it is also to be noted that the RuCuNi/CNTs catalysts exhibited excellent catalytic activity in recycling tests, where 61.9% of the initial activity was retained after five cycles.^28^

Sen *et al.* reported superior catalytic activity for the dehydrocoupling of dimethylamine borane over trimetallic PdRuNi NPs supported on graphene oxide.^121^ With a TOF of 737 h^–1^, this was reported as one of the leading catalytic activities in this field, along with RuPtNi/GO (TOF = 727 h^–1^)^268^ and RuCo/f-MWCNT (TOF = 775 h^–1^).^269^ In contrast, TOFs of 38 and 272 h^–1^ were reported for the dehydrocoupling of dimethylamine borane over Pd/GO and PdNi/GO, respectively.^270,271^ The superior catalytic performance observed was ascribed to the synergy of the three alloyed metals along with the small diameter (ca. 3.8 nm), high monodispersity, and high metallic ratio. The prepared trimetallic nanocomposites showed excellent catalytic performance and can be considered as a competitive alternative to other catalysts to obtain hydrogen gas from the dehydrocoupling of dimethylamine borane.

The use of TMNP catalysts for dehydrogenation reactions reflects the emerging use of TMNP catalysts in general. Publications involving the use of TMNPs for dehydrogenation reactions have appeared only within the last 10 years, alluding to the novelty of such catalytic systems. From the examples detailed in this section and collated in Table 7, the addition of a third metal has proven beneficial in many systems, particularly the dehydrogenation of formic acid and ammonia borane as well as the generation of alkenes and alkynes. In many cases, the addition of a third metal significantly enhanced the catalytic activity and improved the selectivity for the desired product through electronic modification and/or morphology control of the NPs.

### 4.3 Catalytic Oxidation Reactions over TMNPs

Catalytic oxidation reactions play crucial roles in the contribution of green and sustainable processes and in the production of key intermediates and chemicals. Oxidations often lead to low selectivity and can be difficult to control, and therefore, selective oxidation requires careful design of catalysts to overcome this challenge.^272^ Au- and Pd-based catalysts are among the more commonly used materials in catalytic oxidations of alcohols and polyols.^273−275^ The continual research into finding more active, selective, and stable catalysts is apparent, and alloying of other transition metals with noble metals is desirable to enhance the catalytic performance and also reduce the cost.^276^ Herein we discuss the impact of a variety of reports of oxidation reactions over TMNP catalysts.

#### 4.3.1 Oxidation of Glucose and Alcohols

Oxidation of abundant chemicals such as glucose can generate important intermediates that are used in the food and pharma industries; however, glucose oxidation is typically achieved *via* enzymatic catalysis, and therefore, high catalytic activity is demanded of heterogeneous catalysts for them to be considered as a replacement technology.

**Figure 20:**
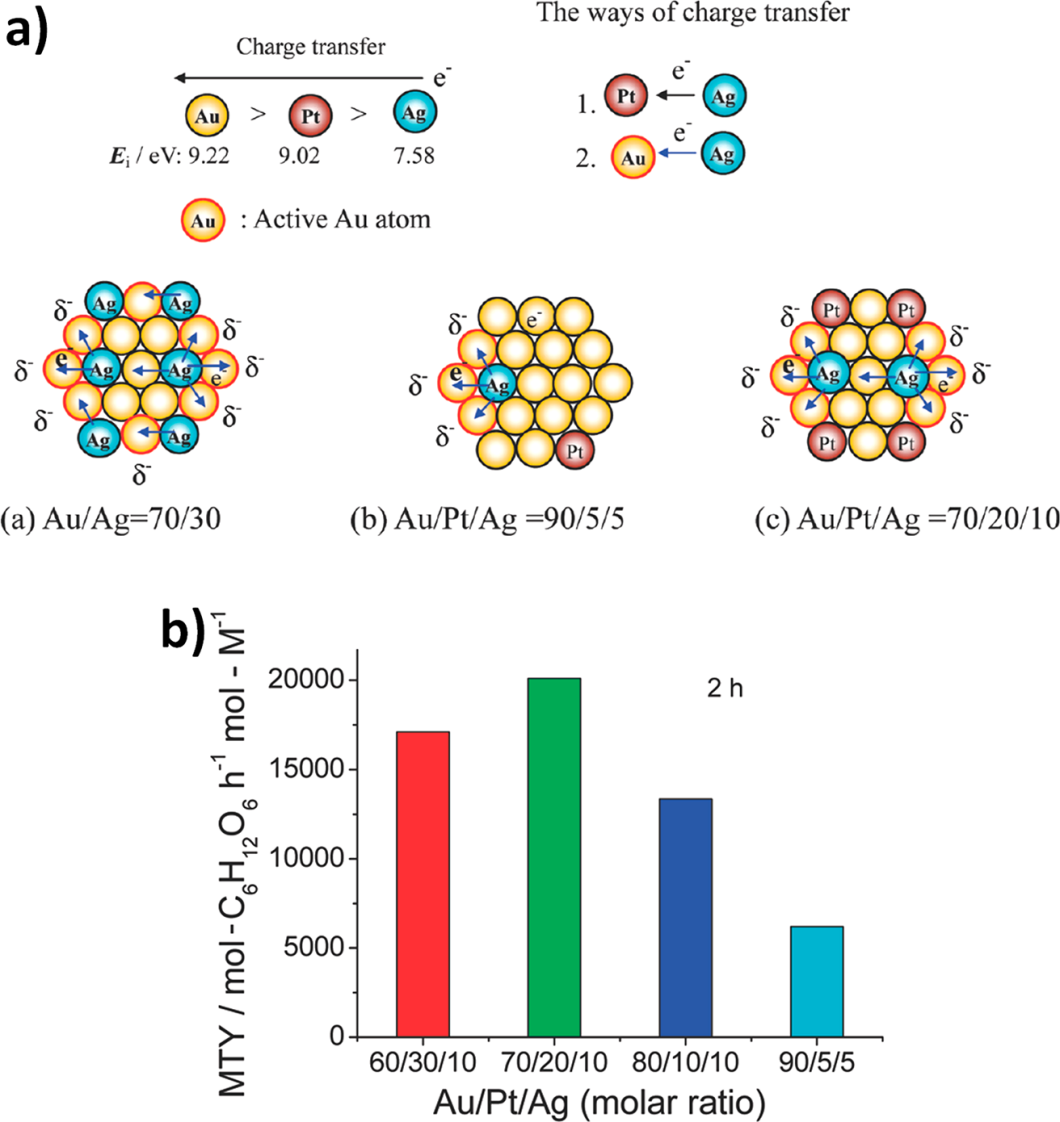
(a) Illustration of potential electronic charge transfer routes in PVP-protected Au/Ag and Au/Pt/Ag NPs. (b) Metal time yield (MTY, in units of moles of glucose per hour per mole of metal) over TMNPs with different compositions, highlighting the advantages of an optimized metal ratio according to improved charge transfer. Adapted from ref (277). Copyright 2011 American Chemical Society.

Polymer-protected Au_70_Pt_20_Ag_10_ NPs exhibited high catalytic performance for the aerobic oxidation of glucose, with a high average activity of 20 090 mol_glucose_ h^–1^ mol_M_^–1^ at 2 h (3.8 times higher than that of monometallic Au NPs) under operating conditions of 333 K and 0.1 MPa O_2_.^277^ The Au_70_Pt_20_Ag_10_ catalyst was reported to comprise small-diameter particles (*ca.* 1.5 nm) formed by a chemical reduction method and protected by PVP. It was concluded that in addition to the small NP size, electronic charge transfer from adjacent elements resulted in higher catalytic activities in comparison with those of mono- and bimetallic NPs. With the addition of Ag as the third metal, this species could donate electrons to Au and Pt (the ionization energies of Ag, Au, Pt are 7.58, 9.22, and 9.02 eV, respectively) (Figure 20). Compared with the bimetallic system, where there is only one route for charge transfer, the TMNP system had two further charge transfer routes: Ag → Au and Ag → Pt. The synergistic effect of the combination of the three metals was used to explain the increased activity.^277^ To support the synergistic postulate, XPS measurements and DFT calculations indicated the presence of negatively charged Au atoms in such trimetallic alloys that were formed through electron transfer from Ag. The negatively charged Au atoms were thought to be the crucial active sites in glucose oxidation. For the study outlined, it is also noted that 70% of the initial activity was maintained after four cycles, giving a total of 8 h for the long-term activity of the aerobic oxidation of glucose.

**Figure 21:**
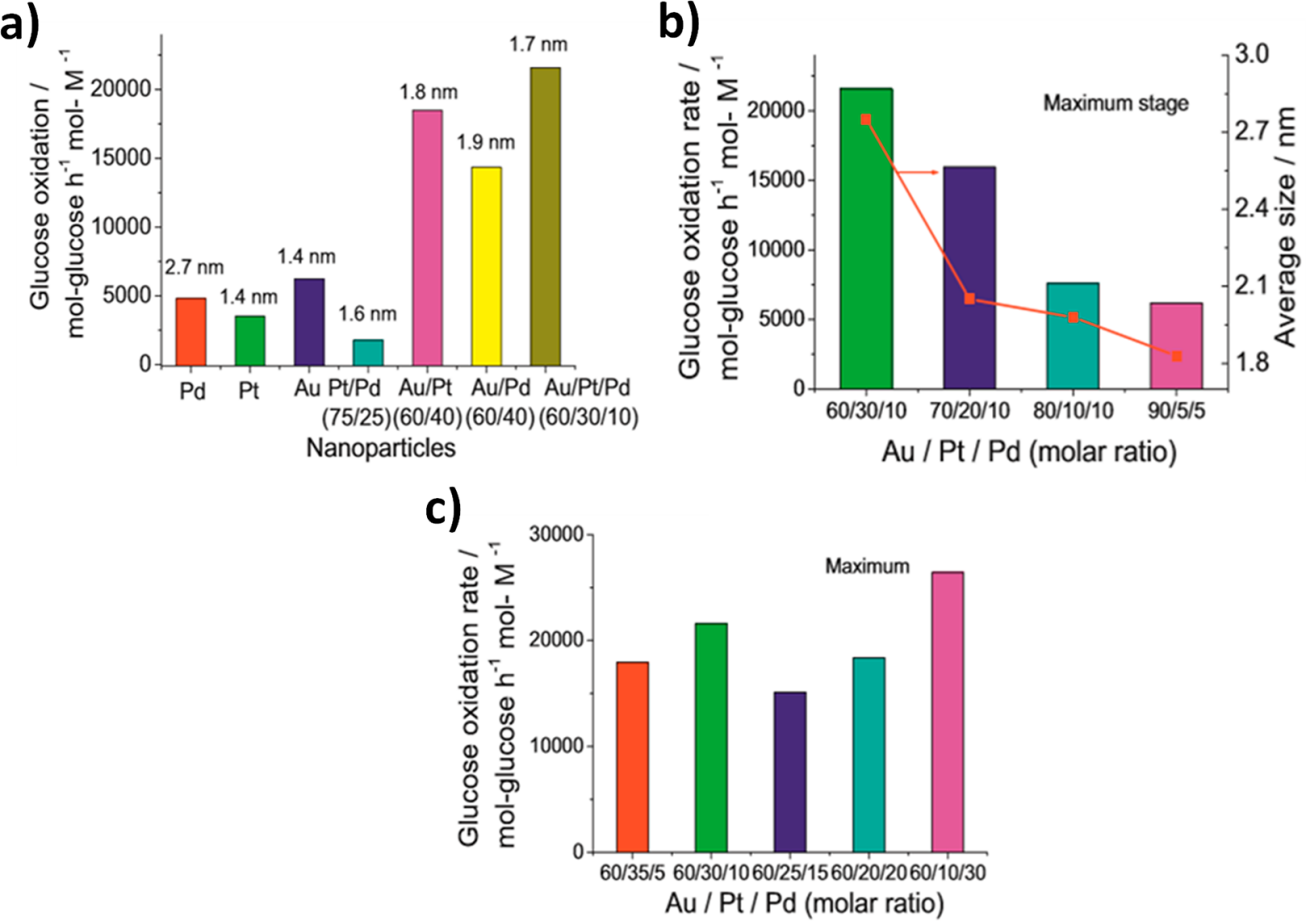
(a) Catalytic activities of Pd, Pt, Au, Pt_75_Pd_25_, Au_60_Pt_40_, Au_60_Pd_40_, and Au_60_Pt_30_Pd_10_. The corresponding mean diameters of the nanoparticles are shown. (b) Catalytic activities of AuPtPd nanoparticles with Au fractions of 60, 70, 80, and 90%. (c) Catalytic activities of AuPtPd nanoparticles with 60% Au. Adapted with permission from ref (80). Copyright 2013 Elsevier.

Subsequently, Zhang *et al.* reported the use of AuPdPt TMNPs in a colloidal suspension for the aerobic oxidation of glucose.^80^ TMNPs with a variety of Au:Pt:Pd atomic ratios were synthesized, and the particles were compared with monometallic Pd, Pt, and Au and bimetallic Pt_75_Pd_25_, Au_60_Pt_40_, and Au_60_Pd_40_ materials. On the basis of TEM images, the reported diameters of these nanoparticles ranged from 1.3 ± 0.5 nm (Au_60_Pt_10_Pd_30_) to 2.1 ± 0.6 nm (Au_70_Pt_20_Pd_10_). The reported catalytic activities for the aerobic oxidation of glucose over the various species indicate that an improvement in catalytic activity can be achieved *via* addition of a second metal. The Au_60_Pt_40_ bimetallic nanoparticles exhibited a 4-fold increase in the conversion rate in comparison with their monometallic counterparts. Upon the addition of a third metal, such as in the case of Au_60_Pt_30_Pd_10_, a further increase in the rate was achieved in comparison with the bimetallic compositions; however, the data in Figure 21b highlight how variance in the Au:Pt:Pd ratio can drastically influence the catalytic activity. Higher concentrations of Au resulted in a reduced rate, with Au_90_Pt_5_Pd_5_ having a rate similar to that for monometallic Au, corresponding to a 4-fold decrease in activity compared with Au_60_Pt_30_Pd_10_. Figure 21c illustrates that variations in the stoichiometry of Pt and Pd, with the concentration of Au fixed at 60%, had an impact on the catalytic performance. The optimal stoichiometry was reported as Au_60_Pt_10_Pd_30_, with a glucose oxidation rate of 26 430 mol_glucose_ h^–1^ mol_M_^–1^. The activity of this composition is more than 5 times greater than those of the corresponding monometallic NP catalysts and more than 15% greater than for the bimetallic compositions such as Au_60_Pt_40_.

**Figure 22:**
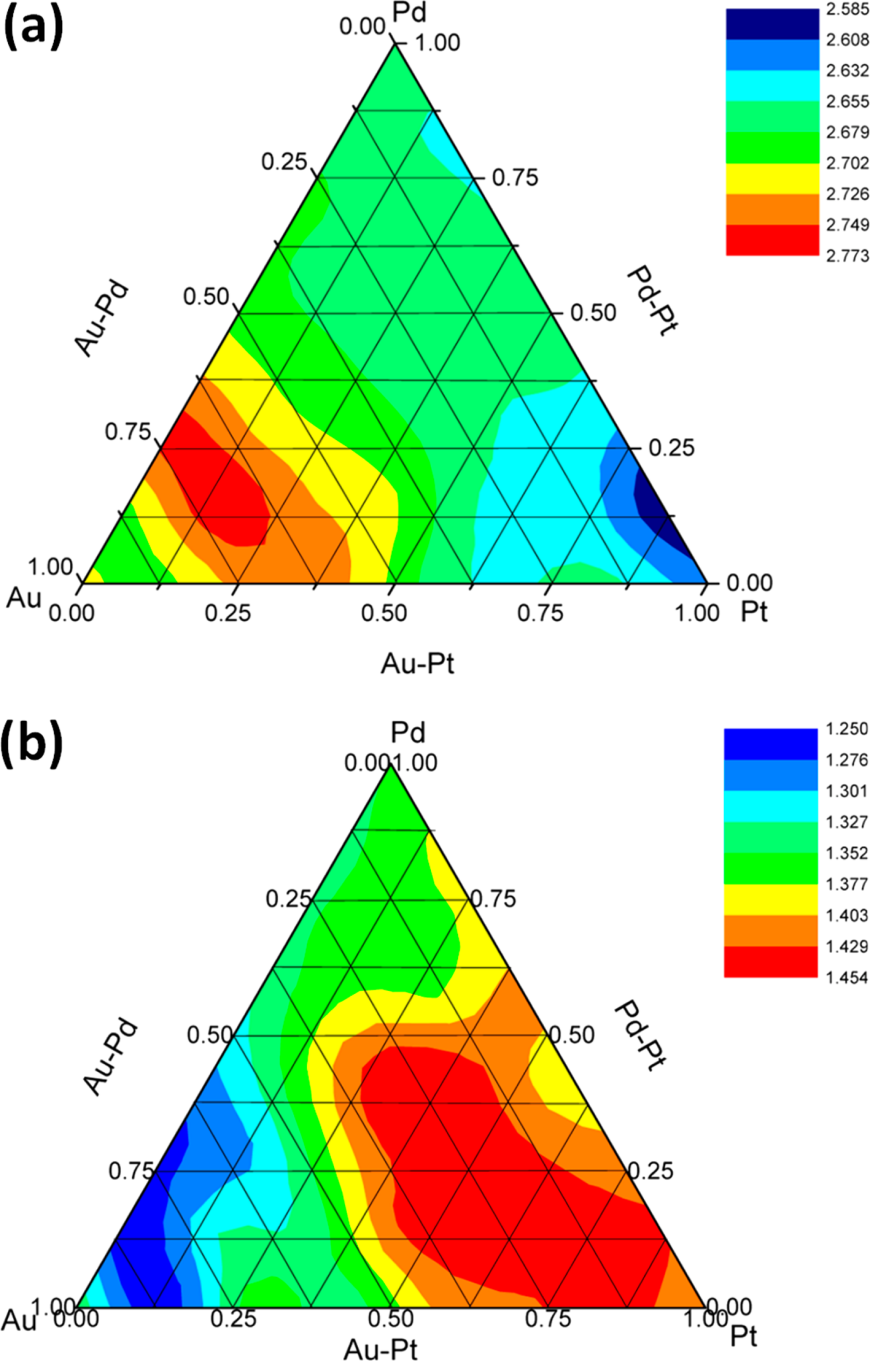
Ternary contour maps of (a) calculated average Au–Pd, Au–Pt, and Pd–Pt distances (in Å) for Au_*x*_Pd_*y*_Pt_*z*_ (*x* + *y* + *z* = 7) clusters and (b) calculated O–O distances (in Å) in Au_*x*_Pd_*y*_Pt_*z*_–O_2_ (*x* + *y* + *z* = 7) clusters as functions of the atomic composition. Adapted from ref (278). Copyright 2017 American Chemical Society.

The increase in activity in the highlighted work was again concluded to originate from electronic charge transfer between elements, which is due to their different electronegativities. In this case, Pd is the most electropositive metal and donates electron density to neighboring Au and Pt atoms. DFT calculations confirmed the Mulliken charges on a simulated Au_37_Pt_12_Pd_6_ nanoparticle to be −0.035 for Au, −0.024 for Pt, and +0.168 for Pd. The induced (slight) negative charge of the Au atoms could activate O_2_ molecules by donation of electronic charge into the antibonding orbital of the O atom to form a superoxo-type radical; this highly active oxygen species then would promote the glucose oxidation reaction, which in turn is facilitated by the Pt and Pd sites.^80^ Zhao *et al.* investigated various TMNP AuPdPt compositions with DFT calculations and observed that Au took exposed positions (lower CN) with Pd in the center (higher CN).^278^Figure 22a illustrates the calculated average interatomic distances of the cluster according to the composition. Interestingly, longer O–O bonds appear on clusters with shorter average metal–metal distances as the cluster composition changes. Subsequent calculations indicated that high Pt and low Au content yielded a greater oxygen binding energy and shorter bonds, while the largest O_2_ bond elongation and lowest binding energies were observed for Au_2_Pd_2_Pt_3_ and Au_2_PdPt_4_. Mixing was preferred over segregation of bi- and monometallic phases, and O_2_ favored binding in the order Pt > Pd > Au, as depicted in Figure 22b. The observations were attributed to lower vertical ionization potentials and electron affinities of clusters with higher Pd content. The adsorption energy increased with decreasing Au content, and this was approximately correlated with the electron transfer from the metal cluster to O, which itself aligned with the highlighted experiment.^278^

Selective oxidation of glycerol can yield a variety of useful commercial products and intermediates in organic synthesis, including glyceric acid (GLYA), dihydroxyacetone (DHA), and tartronic acid.^279,280^ With regard to glycerol oxidation, it is crucial to tune the selectivity, overcome deactivation caused by strong adsorption of acids and ketones, and avoid overoxidation.^281^ Kimura *et al.* pioneered a commercial Pt/C catalyst with added bismuth.^282^ The DHA selectivity increased from 10% to 80% when Bi was added to the parent Pt/C catalyst, and it was proposed that surface submonolayers of bismuth acted as site blockers on Pt(111), controlling the glycerol orientation.

Earlier studies used basic conditions for glycerol oxidation, but base-free conditions have recently been developed due to the use of base additives forming impurities.^281^ Additional studies have been conducted to further enhance the commercial Pt/C and BiPt/C catalysts (where C is activated carbon). Villa *et al.* modified AuPt/C with bismuth (0.1–1 wt %) and compared the resulting catalysts to the commercial catalysts (Pt/C and BiPt/C) in the base-free oxidation of glycerol.^283^ Adding 1 wt % Bi to AuPt/C had no significant effect on the activity (TOF = 585 h^–1^) compared to nonmodified AuPt/C (541 h^–1^). Conversely, a higher TOF (773 h^–1^) was observed with the addition of only 0.1 wt % Bi. In comparison, commercial Pt/C and BiPt/C exhibited activities of 586 and 914 h^–1^, respectively (353 K, *p*O_2_ = 0.3 MPa, base-free). High amounts of Bi were thought to block the active sites, reducing the promotional effect of Bi addition, as seen in previous studies using addition of 1–3 wt % Bi to a bimetallic AuPd catalyst.^284^

In terms of selectivity, the addition of Bi directed the oxidation of the secondary alcohol function, yielding DHA as the main product. In the absence of Bi, glyceric acid was the main product, as oxidation of the primary alcohol group was favored; however, the selectivity for DHA depended on the amount of Bi, with 1 wt % Bi decreasing the DHA selectivity. With a low Bi loading, stable DHA selectivity (63–65%) with conversion was obtained, in contrast to a drastic drop in selectivity over commercial BiPt/C, where at 30% and 80% conversion the selectivities were 53% and 35%, respectively. Analysis of XPS measurements revealed that Bi did not modify the electronic properties of Au and Pt; therefore, the group proposed that the change in selectivity was due to a change in the geometric nature of the NPs.^284^

Recycling tests were performed to compare 0.1 wt % Bi-modified AuPt/C with commercial BiPt/C (4 h reaction, catalyst filtered and reused after each run without any further treatment). Interestingly, 0.1 wt % BiAuPt/C showed better resistance to deactivation, as Au enhanced the stability by limiting the leaching of Bi, whereas BiPt/C resulted in high Bi leaching, as confirmed by ICP measurements.^283^

Other bimetallic systems have shown considerable activities toward glycerol oxidation, with a mixture of selectivities for GLYA or DHA, including PdPt/TiO_2_,^25^ AuPt/NiO,^285^ PtBi/MWCNTs, and PtSb/MWCNTs.^286^ Kondrat *et al.* prepared AuPdPt TMNPs supported on TiO_2_ by sol immobilization and reported enhanced catalytic performance for base-free glycerol oxidation (373 K, *p*O_2_ = 0.3 MPa, 0.5 h).^25^ Bimetallic PdPt/TiO_2_ showed high selectivity for C_3_ products. However, the addition of Au as the third metal increased the catalytic activity: the TOF increased from 210 h^–1^ over PdPt/TiO_2_ to 378 h^–1^ over the trimetallic AuPdPt/TiO_2_ catalyst. Notably, monometallic Pt NPs were reported to prevent toluene formation in glycerol oxidation. After the first reusability test, the particle diameter was determined to be *ca.* 2.6 nm by TEM. The catalyst showed small amounts of leaching and agglomeration after the second reuse, and the average particle diameter increased to 4.3 nm, indicating instability after two runs.

Using sol immobilization preparation, the same group also supported AuPdPt NPs on activated carbon, and the catalytic activity for the solvent-free oxidation of benzyl alcohol was investigated.^133^ Detailed aberration-corrected STEM-EDX analysis confirmed the formation of ternary alloys but also indicated compositional differences between particles for the trimetallic catalysts. The fluctuation in composition varied systematically with NP size, as larger particles were Pd-rich whereas smaller particles were Pd-deficient—an example of size-dependent composition. Ostwald ripening could also be a factor in the Pd-rich particles.^287^ In comparison, the compositional variation was not observed in the AuPt bimetallic system. Oxidations were carried out for 2 h at 393 K and *p*O_2_ = 1.0 MPa. Over a 1 wt % Au_0.45_Pd_0.45_Pt_0.1_/C trimetallic catalyst, a TOF of 31 900 h^–1^ was reported, with 80.2% selectivity for benzaldehyde. In contrast, over monometallic Pt/C, a TOF of 2910 h^–1^ was recorded, but a high selectivity of 90.7% for benzaldehyde. Over bimetallic Au_0.5_Pt_0.5_/C, a TOF of 8450 h^–1^ was reported, with 84.9% selectivity for benzaldehyde. Addition of low levels of Pt to the AuPd system resulted in higher selectivity by suppression of toluene formation, which was thought to be due to the enhanced relative stability of platinum hydride in comparison with palladium hydride.^272^

#### 4.3.2 Oxidation of Carbon Monoxide to Carbon Dioxide

Carbon monoxide is a well-known toxic gas and a pollutant with various environmental and health-related problems. Catalytic CO oxidation is an important research area because of its applicability in several processes, such as methanol synthesis, the water gas shift reaction, and automotive exhaust controls.^288−290^ Haruta *et al.* demonstrated that supported Au NPs exhibited high catalytic activity when the diameter of the Au NPs was less than 5 nm.^291^ The excellent affinity of Au to selectively bind with CO enhances the catalytic activity, and CO oxidation is thought to take place at the interface between the Au metal NP and an oxide support, such as ZnO, TiO_2_, or CeO_2_.^45,292^ Pt metal catalysts are also highly active for CO oxidation,^293^ but other non-noble metals such as Fe, Co, Ni, and Cu have received recent focus due to the motivation to reduce cost.^294,295^ High activity, selectivity, and stability are constant challenges in heterogeneous catalysis, so it is crucial to design catalysts with highly active sites, for example, to render them useful in applications. Adding a second or third metal can not only increase the activity, selectivity, and stability through synergistic effects but also reduce the price, for example by reducing the weight percent of costly platinum-group metals by addition of other metals such as Fe and Cu.

There is limited literature on the use of TMNPs as catalysts for CO oxidation, but successful reports concern mono- and bimetallic catalysts.^293,296−298^ Yang *et al.*(276) synthesized PtNiCo alloyed NPs *via* a polyol method with hexadecanediol as the reducing agent, and three different support materials were investigated: carbon black, silica, and titania. The CO conversion rates (in terms of Pt-specific mass activity of the NPs supported on carbon black) followed the trend PtNiCo/C > PtCo/C > PtNi/C. Pt was the active site, but the combination of Pt, Ni, and Co in the alloy led to enhanced activity and stability in comparison with the mono- and bimetallic counterparts. Ni was responsible for the enhanced stability, while the synergy of adding Co promoted the activity. The formation of PtNiCo trimetallic alloy sites promoted oxygen activation on the surface, facilitating the adsorption and activation of CO and O_2_. Overall, the study highlighted the importance of oxygen-activation sites on the nanoalloy trimetallic surface and the tunability of active sites related to support–nanoalloy interactions.

Tripathi *et al.* studied a 55-atom Pt_31_Ni_12_Co_12_ FCC catalyst using DFT modeling approaches.^299^ The system exhibited limited CO poisoning, which is advantageous, but was inferior to PtNi for CO oxidation activity. The occupancy of d states and the position of the d-band center were used to determine optimal alloying metals with Pt and revealed that lowering the back-donation ratio was key to reduce the CO binding energy. There remains a trade-off between activity and reduction in CO poisoning, and a balance can be struck by analysis of the occupancy of d states and the position of the d-band center, which can be harnessed in further work in this field.

Zhu *et al.* tested highly mixed PtPdRh FCC (111) and (100) slabs with a major focus on C–C splitting and CO oxidation.^181^ They found that alloy systems were better for C–C bond cleavage over monometallic equivalents, indicating synergy. Pd in the Pt–Pd–Rh nanocrystals was shown to promote activity and durability by offering oxidative hydroxyl groups that facilitated the oxidation of adsorbates. For (100), Pd-rich compositions are favorable for C–C cleavage; for (111), the highest reactivity was observed with a 1:1:1 composition, which was considered suitable for CO oxidation as well. The CO oxidation capability increased with increasing Pt content in ternary nanotruncated octahedrons as a result of a lower energy barrier, while Rh improved the selectivity for CO_2_.

#### 4.3.3 Partial Oxidation of Methane to Methanol

The direct formation of methanol from methane remains a promising process to generate an important chemical with the potential to be used to form fuels or resins, for example. Methane reserves have been estimated to exceed 2 × 10^14^ m^3^.^300^ Currently, the dominant method of converting methane to methanol is a two-step process. The first step involves the conversion of methane to syngas (CO + H_2_), and this step is highly energy-intensive. The second step converts syngas selectively to methanol over a methanol-selective catalyst,^301^ which is highly selective, but again the high energy demand and harsh conditions of the process leave much room for improvement. The direct or one-pass conversion of methane to methanol, for example, is characterized by high selectivity; however, the low conversion of methane provides the main challenge.

Previous studies have used H_2_O_2_ as the oxidizing agent;^302−305^ however, the use of concentrated H_2_O_2_ is associated with its own challenges, including storage and transport. Therefore, the catalyst must both catalyze the direct synthesis of H_2_O_2_ and the oxidation of methane. Previous reports have detailed a supported BMNP formulation, AuPd/TiO_2_, to catalyze the oxidation of methane with H_2_O_2_ in water at 323 K.^306,307^

With the addition of copper, Ab Rahim *et al.*(308) expanded the AuPd/TiO_2_ system to a trimetallic CuAuPd/TiO_2_ system in order to investigate the effect of a third metal on the catalysis. The effects on the H_2_O_2_ production, methanol production, and methanol selectivity were investigated. Catalysts with various stoichiometries were prepared using the IWI method. In a process relying on *in situ* generation of H_2_O_2_, the addition of Cu actually led to a sharp decrease in catalytic performance in methanol production in comparison with the AuPd/TiO_2_ system. Using 2.5 wt % Au and 2.5 wt % Pd on TiO_2_ resulted in an activity of 0.9 mol_CH_4_converted_ kg_cat_^–1^ h^–1^ with a methanol selectivity of 68.2%; the activity using CuAuPd/TiO_2_ (2.5 wt % loading of each metal) was 0.1 mol_CH_4_converted_ kg_cat_^–1^ h^–1^ with a methanol selectivity of 81.8%. The TOF of the trimetallic material further diminished with decreasing Cu loading, and no activity was recorded over 2.5 wt % Cu/TiO_2_.

When H_2_O_2_ was added to the reaction mixture, far greater activity was recorded, and the TMNP system proved to be superior. AuPd/TiO_2_ showed an activity of 1.9 mol_CH_4_converted_ kg_cat_^–1^ h^–1^ but only 49.3% methanol selectivity, and other bimetallic combinations gave low activities and selectivities toward methanol. The addition of 2.5 wt % Cu boosted the activity to 2.2 mol_CH_4_converted_ kg_cat_^–1^ h^–1^, but further compromised the methanol selectivity to 27.8%. However, when 1.0 wt % Cu was used, a slightly higher activity of 2.7 mol_CH_4_converted_ kg_cat_^–1^ h^–1^ was recorded, with a methanol selectivity of 82.7%.

Three possible explanations were proposed for the increase in catalytic performance with the addition of Cu. First, the redox properties of the AuPd sites could have been altered with the addition of Cu through electronic inductive effects. Second, the Cu could have blocked or neutralized active sites for undesirable H_2_O_2_ decomposition pathways. Third, the Cu itself could have lowered the rate of H_2_O_2_ decomposition.

XPS was used to analyze the CuAuPd/TiO_2_ (1.0 wt %, 2.5 wt %, and 2.5 wt %, respectively) following calcination at 673 K, and Cu^+^ and Cu^0^ were present, but Cu^2+^ was not. Pd was present as both Pd^2+^ and Pd^0^, with an atomic ratio of 6.14:1. Little change in the Pd/Cu ratio was observed, indicating that the formation of Au@Pd with Cu highly dispersed across the TiO_2_ surface is more likely. The mean diameter of the nanoparticles was 1.2 nm, as measured with TEM imaging; however, EDX studies of such particles with greater resolution would be required to positively support the conclusion of a core@shell structure as postulated on the basis of the XPS measurements, particularly with respect to the small NP size.

With regard to catalytic oxidation reactions, reports on TMNPs as catalysts are limited, and ternary metal oxides as catalysts dominate.^309−311^ From the stated examples, however, the benefits that alloying a third metal into a binary system can provide to catalytic performance are evident.

### 4.4 Electrochemical Reactions

TMNPs have recently garnered much attention as catalysts in many electrochemical oxidations and reductions. With the ongoing environmental cost related to high usage of nonrenewable fossil fuels, the search for renewable, efficient, sustainable, and green energy sources is of high importance. Electrochemical oxidation of methanol,^312−320^ ethanol,^321−326^ and other simple oxygen-containing organic compounds^327^ to give carbon dioxide and water is a potential candidate to meet this demand.

#### 4.4.1 Electrochemical Oxidation of Alcohols

Methanol possesses a high energy density, and the products of the methanol oxidation reaction are CO_2_ and H_2_O, both of which can be recycled and reused in other processes. Methanol is also easy to store and transport in large quantities. The current commercial catalyst for the direct methanol fuel cell is Pt/C,^328^ but the kinetics for the oxidation reaction of methanol are slow, and intermediates are formed that poison the catalyst, such as CO, formaldehyde, and formic acid.^329^ The addition of a second metal, notably Ru, increases the catalytic performance considerably as a result of electronic and geometric effects.^175^ Addition of Ru adsorbs ^•^OH at a lower potential than Pt, so Ru–OH_ads_ is formed more readily than Pt–OH_ads_ and oxidizes CO_ads_ to CO_2_, liberating the catalytic site of the strongly adsorbed CO molecule that would poison a Pt-only catalyst. Cu has also been reported as a second metal, since the Pt–Cu interaction shifts the d band of Pt, reducing the strength of adsorption of CO on Pt.^330,331^

**Figure 23:**
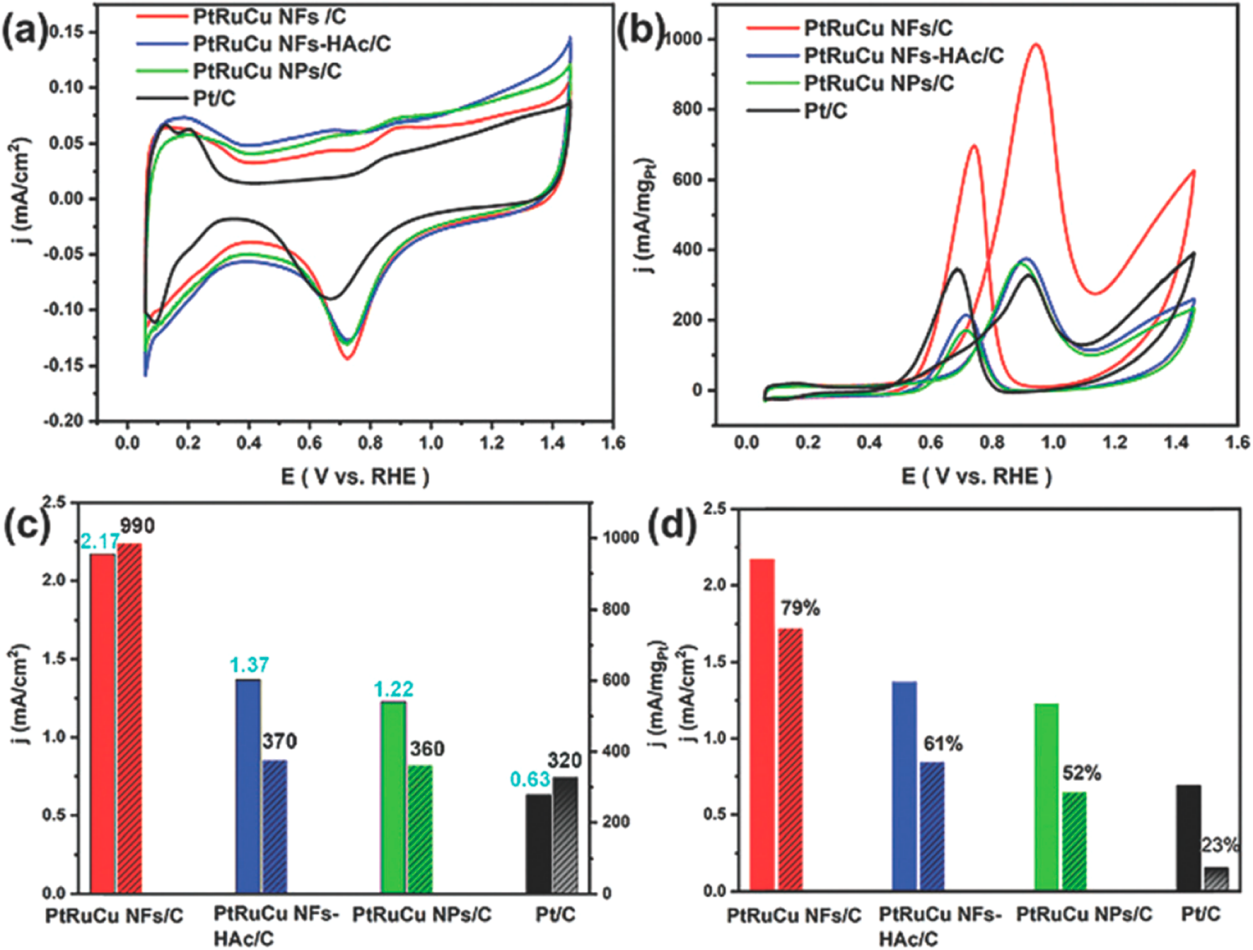
(a) Cyclic voltammograms obtained for the methanol oxidation reaction in N_2_-saturated aqueous HClO_4_ over PtRuCu nanoframes and carbon-supported PtRuCu and Pt NPs. (b) Mass activities of the catalysts. (c) Specific activities, with peak current density on the left and mass activity on the right. (d) Durability comparison following 800 CV cycles. Adapted with permission from ref (175). Copyright 2020 Chinese Chemical Society, Institute of Chemistry of the Chinese Academy of Sciences, and Royal Society of Chemistry.

Yin *et al.* reported the use of trimetallic PtRuCu nanoframes for the electrochemical oxidation of methanol (Figures 11 and 23).^175^ The PtRuCu nanoframes aim to combine the promotional effects of the addition of Ru and Cu to the Pt-only catalyst, resulting in a catalyst with activity superior to that of bimetallic formulations. Figure 23b illustrates that the commercial Pt/C catalyst exhibits an electrocatalytic performance normalized to catalyst mass of 0.32 A mg_Pt_^–1^, whereas the PtRuCu/C nanoframe catalysts were 3 times more active, with an activity of 0.99 A mg_Pt_^–1^. The unetched PtRuCu/C TMNP catalyst had an activity of 0.36 A mg_Pt_^–1^, which was marginally greater than Pt/C (Table 8), demonstrating the increase in catalytic performance that can be achieved from high exposure of low-coordinate metal sites.

**Table 8:** Summary of Catalysts Used for the Electrochemical Oxidation of Methanol

catalyst	catalyst type	performance^a^	ref
PtRuCu/C	supported trimetallic nanoframe	MA = 0.99; SA = 2.17; decay after 800 cycles = 21%	(175)
	supported TMNP	MA = 0.36; SA = 1.22; decay after 800 cycles = 48%	
PtCu/C	supported BMNP	MA = *ca.* 0.5	
Pt/C	supported MMNP	MA = 0.32; SA = 0.63; decay after 800 cycles = 77%	
Au_oct_@PdPt	colloidal core@shell TMNP	MA = 1.50; SA = 2.19	(79)
Pt_2_Au_1_Sn_1_/CNT	supported TMNP	MA = 0.50 (acidic)	(126)
		MA = 1.70 (basic)	

a MA is the mass activity in units of A mg_metal_^-1^, and SA is the specific activity in units of mA cm^-2^.

Furthermore, Figure 23c illustrates that the specific activity of the PtRuCu/C nanoframes was 2.17 mA cm^–2^, which is 3.4 times greater than that over Pt/C (0.63 mA cm^–2^). To demonstrate that a modest quantity of Ru could improve the catalytic performance, PtCu/C samples were also prepared. The mass activity of the PtCu/C catalysts was *ca.* 0.5 A mg_Pt_^–1^, half that of the trimetallic formulation. Moreover, the specific activity of the Ru-free sample was one-third of that for the trimetallic sample. Studies with Cu-free samples showed similar results, strongly confirming the synergistic interaction between the three metals within the system.

In further work from the same study, Yin *et al.* reported degradation of the catalytic performance following 800 cyclic voltammetry (CV) cycles (Figure 23d). The PtRuCu/C nanoframe exhibited a 21% decay in activity. By comparison, acetic acid-capped PtRuCu/C samples showed a 39% decay, TMNP samples a 48% decay, and the conventional Pt/C catalyst a 77% decay. All of the trimetallic formulations showed stability superior to that of the conventional Pt/C catalyst. The observations support the importance of Ru and Cu in lowering the strength of adsorption of intermediates that poison the active sites, thus making desorption of these intermediates more favorable. Analysis following the durability study suggested that the PtRuCu/C nanoframes retained their shape and integrity.

The PtRuCu/C nanoframe samples capped with oleylamine proved to be superior to the commercial Pt/C catalyst with regard to both activity and stability, highlighting the benefits of adding additional metals. In this case, the combination of metals used in the study served to improve the durability of the catalyst by increasing the rate of desorption of catalytic poisons; meanwhile, the defect-rich nanoframe structure facilitated greater catalytic performance in the oxidation of methanol.

Kang *et al.* explored a facile one-pot synthesis method to produce highly controlled Au@PdPt TMNPs with a well-defined octahedral Au core and a highly crystalline dendritic Pd–Pt shell (labeled as Au_oct_@PdPt).^79^ The catalysts showed excellent performance for methanol electrooxidation, originating from optimal binding of adsorbate molecules due to improved charge transfer between the core and the shell. Although the morphologies of BMNPs have been widely studied to tune the catalytic performance, in TMNPs this has been comparatively unexplored. The addition of a third metal increases the number of degrees of freedom with respect to the structure–composition–property relationships. In order to control the nucleation and growth kinetics, preformed NP seeds or structure-directing templates are commonly used, but the method developed by Kang *et al.* instead uses dual reducing agents (namely, ascorbic acid and hydrazine) to coreduce the metal precursors. The single-step process is therefore a simpler approach for synthesizing multimetallic nanoparticles with a desirable structure.^79^

**Figure 24:**
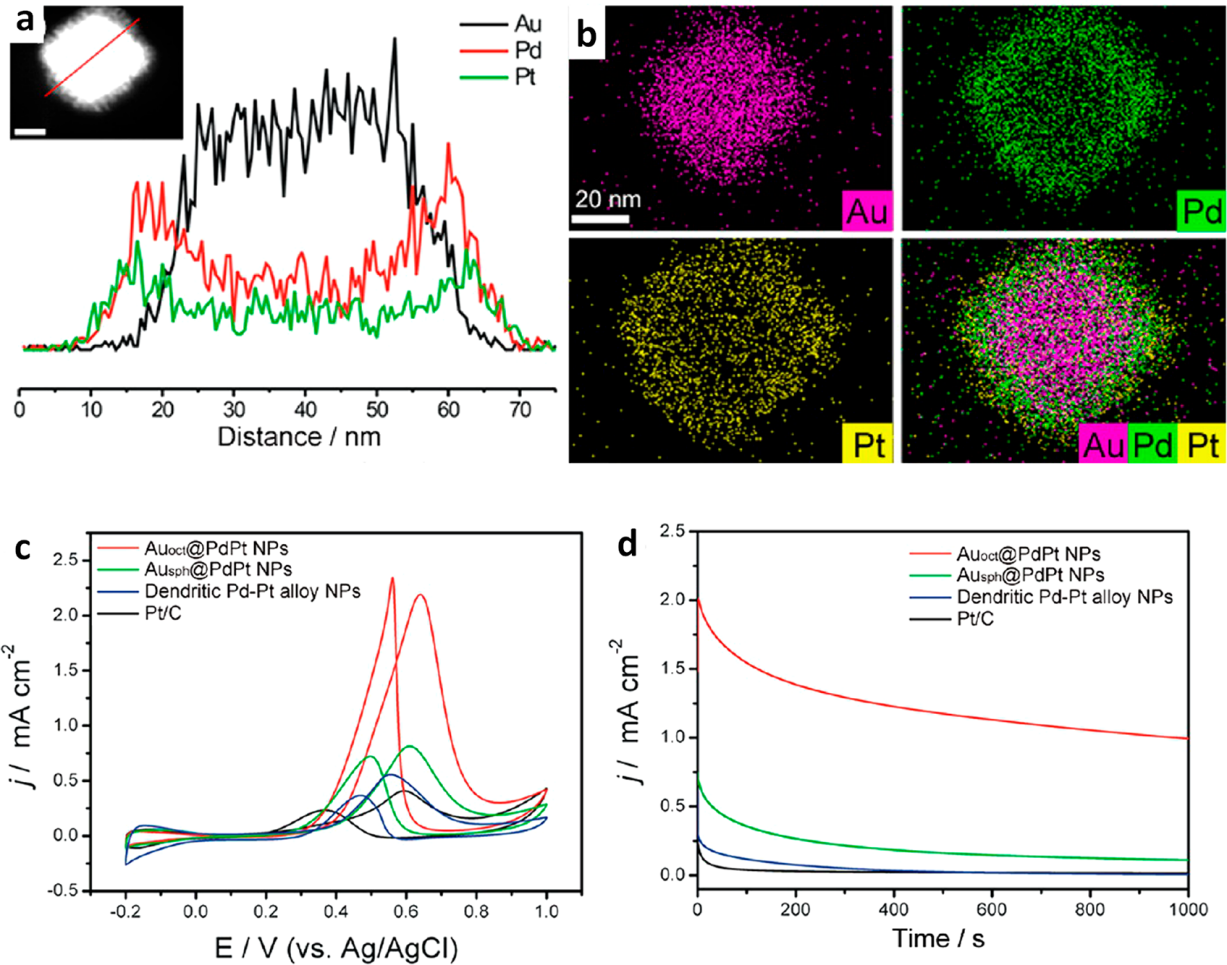
Characterization of the Au@PdPt TMNP electrooxidation catalyst. (a) HAADF-STEM image and cross-sectional compositional line profiles of an Au_oct_@PdPt NP. The scale bar indicates 20 nm. (b) HAADF-STEM-EDX elemental mapping images of an Au_oct_@PdPt NP. (c) CVs of Au_oct_@PdPt NPs, Au_sph_@PdPt NPs, dendritic Pd–Pt alloy NPs, and Pt/C catalysts in 0.1 M HClO_4_ + 0.5 M methanol. Scan rate = 50 mV s^–1^. (d) Chronoamperometry curves for the various catalysts in 0.1 M HClO_4_ + 0.5 M methanol at 0.6 V vs Ag/AgCl. Adapted from ref (79). Copyright 2013 American Chemical Society.

The facile one-pot method involved first mixing the metal precursors (HAuCl_4_, K_2_PdCl_4_, and K_2_PtCl_6_) in an equimolar ratio with CTAC as a stabilizing agent; ascorbic acid was then added, and the mixture was gently shaken. The second reducing agent, hydrazine, was then added, and the mixture was sealed and heated to 95 °C in an oven for 150 min. The resulting NPs were collected *via* centrifugation. SEM and TEM were used to show the high yield (>90%) of octahedral-shaped NPs (Figure 24a). The prepared NPs showed excellent catalytic activity and stability for the electrooxidation of methanol in acidic media. The mass activity and current density of the Au_oct_@PdPt NPs were ca. 1.5 A mg_Pt_^–1^ and 2.19 mA cm^–2^ (Figure 24c,d and Table 8), which compare favorably to those for the nanoframes prepared by Yin *et al.*(175) The enhanced catalytic activity was attributed to the optimized binding affinity of these trimetallic catalysts for the adsorbates due to the improved charge transfer between the core and the shell of the NPs. This conjecture was supported by analysis of the XPS data, as the d-band centers of the Au@PdPt NP catalysts shifted downward compared with that of the PdPt alloy NP catalyst.

Singh *et al.* tested PtAuSn trimetallic catalysts in combination with a range of carbon-based supports for methanol electrooxidation.^126^ Activated carbon is a widely used catalyst support, and it is popular because of its high surface area, which provides good particle dispersion. However, activated carbon is also highly microporous, and this can cause metal nanoparticles to become trapped and inaccessible, which eliminates their catalytic activity. Alternative carbon-based supports, such as CNTs and carbon nanofibers (CNFs), are now emerging. Mono-, bi-, and trimetallic platinum-based catalysts (20% w/w) on different carbon supports were tested for methanol electrooxidation. The CNTs and CNFs have to be pretreated with acid to increase their hydrophilicity in order for the metal(s) to be deposited onto the support. The pretreatment develops functionality on the surface that can be confirmed through the use of Fourier transform infrared spectroscopy (FTIR) and XPS, showing evidence of −OH, C–O, C=O, and C(=O)–O functionalities.

The subsequent evaluation of Pt_2_Au_1_Sn_1_/CNT revealed that this catalyst can be considered among the best catalysts for methanol electrooxidation.^126^ The catalyst was measured to have methanol oxidation mass activities of *ca.* 0.5 A mg_Pt_^–1^ in acidic media and 1.7 A mg_Pt_^–1^ under basic conditions. Although Pt_2_Au_1_Sn_1_/CNF, prepared in the same study, had very reasonable metal dispersion, TEM and XRD analyses indicated that the particle diameter on average was 3.8 ± 1.5 nm (median = 3.7 nm) and that the particle size distribution (PSD) was broad, with only *ca.* 40% of the particles in this 3–4 nm range. Conversely, the Pt_2_Au_1_Sn_1_/CNT catalyst had a smaller average metal particle diameter of 3.3 ± 1.8 nm (median = 2.6 nm), and *ca.* 75% of the particles had diameters of ≤3 nm. The measurement of particle size also showed that the particle size increased slightly with increasing Au content; as the fraction of Au in the metal particles can be reduced in trimetallic catalysts, hence removing its effect on particle size growth, it is advantageous to consider using the trimetallic catalyst over a bimetallic combination.^126^ The unique structure of CNTs appeared to encourage a narrow PSD and small particle diameter of Pt, Au, and Sn TMNP alloys, which make the active sites highly accessible to the reactants, although the reasons for this require further work.

Evidence of an electronic effect caused by the presence of Au was uncovered from analysis of the XPS measurements. In the Pt/AC catalyst, 53% of the platinum was in the oxidized state (as a combination of Pt^2+^ and Pt^4+^); as the Au content was increased to form the Pt_1_Au_4_/C catalyst, the percentage of oxidized platinum was 72%, and in both the trimetallic Pt_2_Au_1_Sn_1_/C and Pt_2_Au_1_Sn_1_/CNT catalysts, the percentage of oxidized platinum was *ca.* 45%. These measurements suggest that the presence of gold in the catalyst enhances the catalytic oxidizing potential of Pt.^126^

Methanol is highly toxic, and therefore, ethanol is considered as an alternative candidate for fuel cell applications. However, the 12-electron reaction for complete transformation is challenging because of the C–C bond cleavage required.^332^ Despite this challenge, bioderived ethanol has a strong case as a renewable resource for energy applications,^333^ particularly as ethanol has a greater theoretical energy density than methanol (8.0 vs 6.1 kWh kg^–1^, respectively).^334^

Zhu *et al.*, reported on the electrochemical lower-alcohol oxidation reaction over TMNP materials.^177^ For the electrochemical oxidation of ethanol (EOR), previous studies had already indicated that Pd is an effective catalyst under basic conditions.^335,336^ As shown elsewhere within this review, the Au_*x*_Pt_*y*_Pd_*z*_ formulation has received much attention in the fast-developing field of trimetallic catalysts; however, as opposed to nanoparticles or nanoframes, one-dimensional nanowires were used in the study reviewed here.^177^

**Figure 25:**
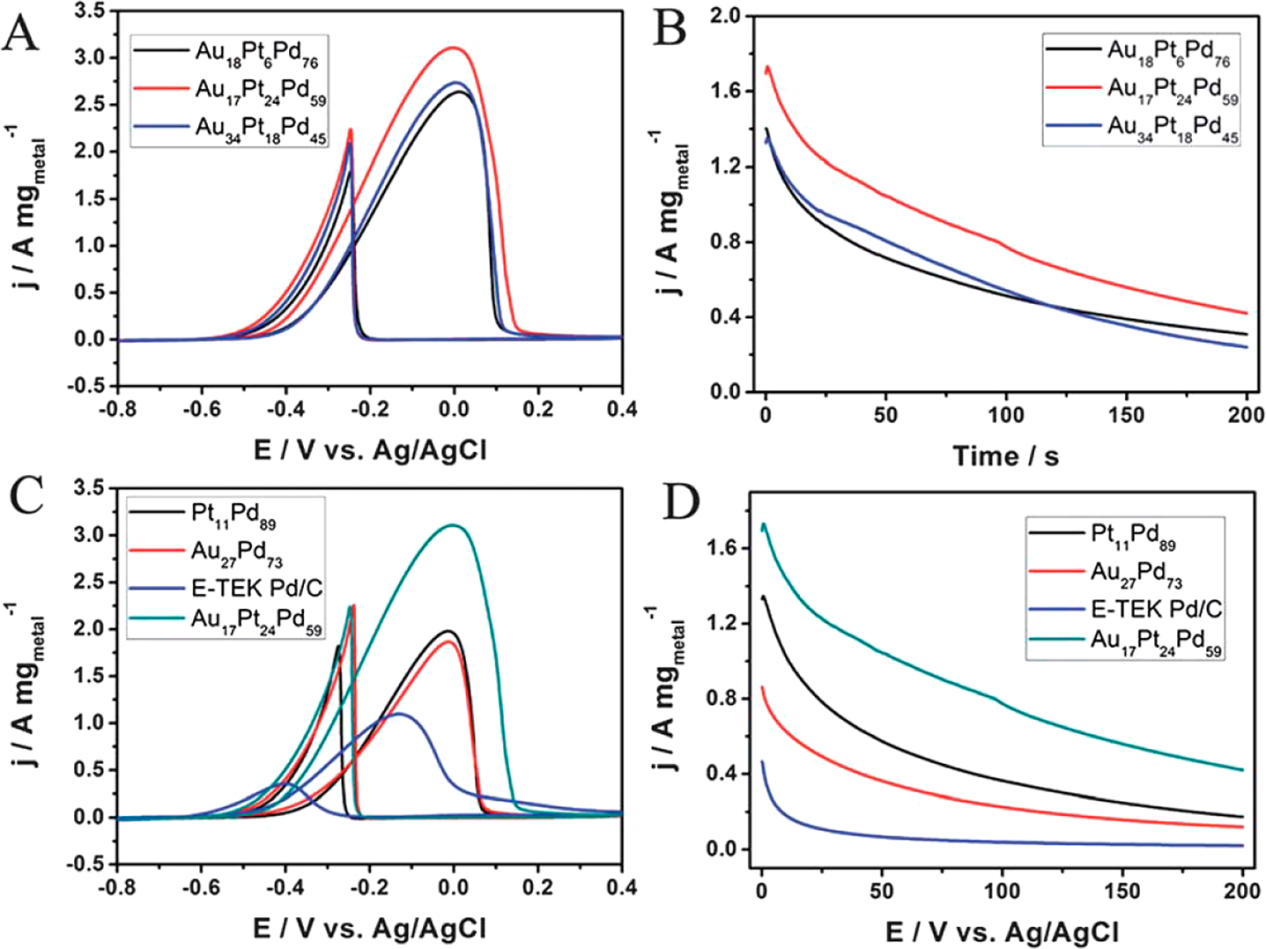
Cyclic voltammetry measurements and current density–time curves in the ethanol electrooxidation reaction. (a) CVs of the three trimetallic catalysts on electrodes under basic conditions. (b) Current density–time curves for the three trimetallic catalysts in a 0.5 M NaOH + 1 M ethanol solution at 0.2 V. (c) CVs of bimetallic Pt_11_Pd_89_ and Au_27_Pd_73_ as well as Au_17_Pt_24_Pd_59_ and commercial E-TEK Pd/C electrodes. (d) Current density–time curves of the bimetallic compositions, Au_17_Pt_24_Pd_59_, and commercial E-TEK Pd/C electrodes at 0.2 V. Adapted with permission from ref (177). Copyright 2012 Royal Society of Chemistry.

The effect of the composition of the trimetallic nanowires was investigated using CV. As the quantity of Pt was increased, the ethanol oxidation peak potential and the onset of the peak potential were negatively shifted, and the peak current increased. Thus, among the three trimetallic compositions tested, the composition with the most Pt (Au_17_Pt_24_Pd_59_) had the highest catalytic activity. Figure 25 illustrates that the peak current density is highest for the Au_17_Pt_24_Pd_59_ composition, where the loading of noble metal was 35.7 μg cm^–2^ for each catalyst. Moreover, Figure 25c shows that the peak current density and catalytic activity of Au_17_Pt_24_Pd_59_ are superior to those of the bimetallic formulations Pt_11_Pd_89_ and Au_27_Pd_73_ as well as the commercial Pd/C catalyst.

A computational study of Pt–AuSnO_*x*_ by Dai *et al.*(337) showed that Pt-based catalysts in which an oxygen-containing species decorates a Pt surface can greatly enhance the ethanol oxidation reaction. Bader charge analysis revealed that in the Pt–AuO catalyst, the small negatively charged O in AuO has a higher-energy O p band and less negative charge to stabilize H and thus was found to be more active than the other screened oxygen-containing species. The high segregation energy of Pt–SnO and the strong oxygen adsorption energy provide durability by preventing dissolution of the Pt–oxygen-containing species into the subsurface or bulk of Pt. Combining the two materials into a trimetallic Pt–AuSnO_*x*_ species increased the mass activity by a factor of 9.7 compared with that of pure Pt while maintaining the desired stability.

It was proposed that in the ethanol oxidation reaction using a Au_*x*_Pt_*y*_Pd_*z*_ catalyst, a CH_3_CO intermediate forms on the Pd surface, and its reaction with adsorbed OH is the rate-determining step because of the low concentration of OH.^338^ The addition of Pt and Au activate the surface, forming OH through electronic synergistic effects. The highlighted study noted that the catalytic performance of the Au_*x*_Pt_*y*_Pd_*z*_ nanowires could be further improved with finer optimizations and adjustments of the Au:Pd:Pt ratio.

Dai *et al.* computationally investigated Pt_3_RhM (M = Fe, Co, Ni, Cu, Ga, In, Sn, Pb) surface models built with the FCC structure and (111) facets using a 2:1:1 composition and four-layer depth in their models for ethanol dehydrogenation. Descriptors developed for selectivity and reactivity were based on the binding energies for O + H_2_O and O + C, respectively; Rh and M were identified to control intermediate adsorption and thus the barrier in the rate-determining step. Furthermore, M influences H_2_O adsorption and tunes the d-band center, which is believed to be responsible for modulating the reactivity. Group IIIA and IVA metals increase the selectivity and preference for CO_2_; this was most impactful for Pt_3_RhSn/C, which exhibited 67- and 7-fold increases in specific activity and mass activity, respectively, when referenced against a commercial Pt/C catalyst.^339^ Further work investigated Pt_6_SnAg nanorods, which showed enhanced EOR activity and stability with a homogeneous Pt–Sn–Ag surface configuration.^340^ The reactivity enhancement was attributed to a structural effect of Sn–Ag pairs formed on the Pt surface, and the homogeneity of the catalyst was highlighted as key to the reactivity.^340^

#### 4.4.2 Oxygen Reduction Reaction

The oxygen reduction reaction (ORR) is of high interest in electrochemistry and has been heavily studied due to its importance in fuel cell technology.^341^ Pt NPs supported on carbon are commonly used as an ORR electrocatalyst, which is characterized by adequate catalytic activity and durability.^342,343^ Research into enhancing the Pt catalyst has shown that introducing additional metals can improve the catalytic performance further as a result of synergistic effects. Tang *et al.*(344) synthesized Au_*x*_Pt_*y*_ electrocatalysts deposited on fluorinated tin oxide and carbon disk substrates. Through the addition of Au, a higher resistance to poisoning in an acidic environment was achieved, with a significant improvement in catalytic performance in an alkaline electrolyte for the ORR. Through the electronic interactions between Pt and Au in alloy particles, the differences in catalytic performance were rationalized. Compared with monometallic Pt electrocatalysts, the overpotential for the reduction was decreased by the bimetallic NPs, leading to an increased cell voltage. Ternary Pd_*x*_Pt_*y*_Ni_*z*_ alloy catalysts were synthesized by a polyol reduction method and were tested for the ORR in proton-exchange-membrane fuel cells. With a narrow particle diameter distribution of *ca.* 5 nm, the Pt-based mass activity of Pd_*x*_Pt_*y*_Ni_*z*_ was double that of commercial Pt; in addition, the long-term durability testing of the trimetallic Pd_*x*_Pt_*y*_Ni_*z*_ alloy within a 200 h operation time was comparable to that of a commercial Pt catalyst.^345^ Furthermore, Mazumder *et al.*(346) highlighted that ternary Pd/PtFe NPs had high durability over 10 000 cycles without loss of the core@shell structure. The use of multiple metals can decrease the total Pt loading in the catalytic system, making such catalysts more attractive for commercial applications with regard to cost and efficiency. A core@shell model with the notion of a thin Pt shell and a core incorporating less expensive and more abundant metals has been considered to improve Pt catalysts; Mazumder *et al.*(346) demonstrated a unique synthesis approach for preparing structured Pd@FePt NPs with a Pd-rich core and a FePt-rich shell. The ORR under acidic conditions was reported to be dependent on the thickness of the FePt shell, where the highest activity and durability were achieved with a shell thickness of <1 nm. The high activity was thought to be due to the change of the electronic structure of Pt upon alloying, but compared to the ORR over a commercial Pt catalyst, the activity was not drastically improved. In terms of the ORR in the study of Mazumder *et al.*, the authors summarized that the interfacial interactions between the FePt shell and the Pd core resulted in higher activity, as upon careful alterations of shell thickness correlating activities were achieved.

**Figure 26:**
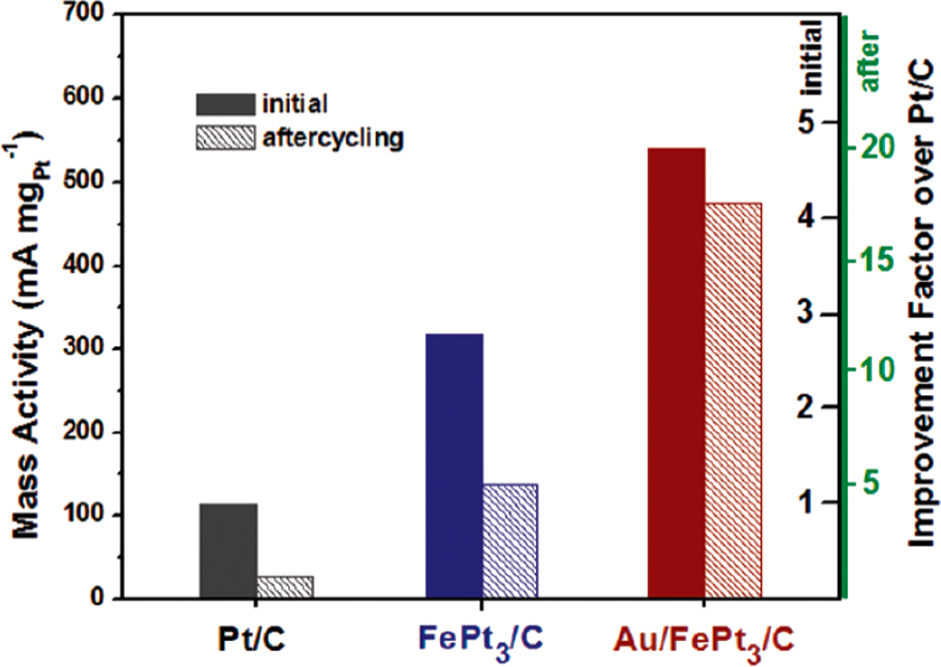
Mass activities of the Pt/C, FePt_3_/C, and Au@FePt_3_/C catalysts before and after 60 000 potential cycles between 0.6 and 1.1 V vs RHE in oxygen-saturated 0.1 M HClO_4_ electrolyte at 20 °C with a sweep rate of 50 mV/s. Adapted from ref (347). Copyright 2010 American Chemical Society.

Other groups have also focused on incorporating multiple metals to modify Pt NP catalysts, concluding that the added synergy, including altering the Pt electronic structure, improves the catalytic performance. Au@FePt_3_ NPs with well-defined surfaces were compared against a commercial Pt catalyst and bimetallic FePt_3_.^347^ The trimetallic Au@FePt_3_ catalyst showed superior durability and activity due to the tailored morphology, composition, and synergy. The initial ORR activity, normalized to the catalyst mass over Au@FePt_3_, was reported to be 5 times higher than that over Pt/C. Figure 26indicates the mass activity before and after 60 000 potential cycles over Pt/C, FePt_3_/C, and Au@FePt_3_/C catalysts, highlighting the durability of the ternary catalyst. TEM characterizations before and after the stability studies were also performed and indicated a considerable change in the size of the Pt and FePt_3_ NPs that was proposed to be due to Ostwald ripening.^348^ In comparison, Au@FePt_3_ NPs were of a similar size and shape before and after the potential cycling, indicating a higher relative durability.

The preparation of TMNP catalysts without Pt, comprising PdCoAu NP alloys, were prepared *via* a microemulsion method and supported on carbon black.^349^ Voltammetric and valence-band spectral measurements indicated a change in electronic structure properties of Pd caused by alloying with Co and Au. The PdCoAu/C system exhibited a decreased density of states at the Fermi level of Pd through a shift of the Pd d-band center. As a result, the formation of O_ads_ or OH_ads_ was inhibited on the Pd surface, leading to enhanced performance in the ORR. The activity at +0.8 V was 0.15 mA cm^–2^ for the as-synthesized Pd/C monometallic catalysts compared with 1.32 mA cm^–2^ over the heat-treated trimetallic PdCoAu/C catalyst.

Modern DFT techniques have become increasingly significant in recent studies of TMNP catalysts for the ORR. Ternary nanoparticles based on Pt–Au–M (M = Cr, Mn, Co, Cu, Zn) were explored by Jennings *et al.* as electrocatalysts for the ORR.^350^ Alloying with Au provided stability under harsh operating conditions at the cathode, and Cr-, Co-, and Cu-rich cores were shown to lead to an icosahedral structure due to the small lattice constants that was determined to be highly catalytically active because of the interplay between strain and ligand effects. Compressive strain exerted on the Au subsurface by the 3d transition metal core led to a favorable weakening of the Pt–OH binding energy, which subsequently resulted in an overall enhancement of the reaction kinetics.^350^

Noh *et al.* investigated alternative Ni@Cu@Pt nanoparticles for the ORR, again using DFT, and observed that there is a strong compressive strain on Pt, leading to the high ORR activity among the nanoparticles according to the d-band center energy model.^351^ At a diameter of *ca.* 3 nm, the electrochemical stability of the TMNPs exceeded that of the monometallic Pt NPs.^352^ An oxygen monolayer coverage exceeding 0.34 induced Cu segregation to the core of the Ni@Cu@Pt TMNPs, implying high stability under reaction conditions between 0.99 and 1.10 V, where up to 0.33 monolayer of O will be available.^352^ Similarly, Flores-Rojas *et al.* studied 44-atom octahedral CoNi bimetallic NPs wrapped in Pt with a Co_*n*_Ni_6–*n*_Pt_38_ (0 ≤ *n* ≤ 6) atomic composition.^353^ The ORR activity was described in terms of O and OH binding energies, which were lower for the trimetallic NPs than for Pt_44_, suggesting an overall higher activity relative to the monometallic Pt catalyst.^353^ In a nicely complementary study, these findings were then confirmed experimentally with two comparable nanoparticle compositions, Co_30_Ni_70_–20Pt/C and Co_70_Ni_30_–20Pt/C, which exhibited 400% and 300% increases in specific activity, respectively, with respect to Pt/C and overall a 1.5-fold higher mass activity than a commercially available Pt/C catalyst.^353^ Cruz-Martínez *et al.*(354) investigated the electrocatalytic activity of M_6_@Pd_30_Pt_8_ (M = Co, Ni, Cu) toward the ORR and observed similarity to Pt_44_; an activity trend of Pt_44_ > binary M@Pd_38_ > Pd_44_ was reported. The O and OH adsorption energies were again evaluated as descriptors of the electrocatalytic activity, and the trend in activity based on compositional variation was shown to be M_6_@Pd_30_Pt_8_ > M_6_@Pd_38_ > Pd_44_,^354^ supporting the inference that the benefits of TMNPs over mono- and bimetallic formulations for this reaction are significant.

Oxygen is activated on Ni@PdPt; calculations indicated that M atoms (M = Ni, Co, Cu) have lower energies in the core in ternary compositions, which changes for bimetallic nanoparticles in an oxidizing environment. Kim *et al.*(355) performed computational analysis on Pt_*x*_Cu_*y*_Pd_*z*_ alloy catalysts for the ORR. They reported that the strength of the bond between surface Pt and adsorbates reaches a maximum when the Pt:Pd ratio reaches 50 atom % and that Pd and Au improve the durability of the (111) surface in an acidic and oxidizing environment while maintaining the catalytic reactivity. The enhanced catalyst stability is crucial for achieving the durability target for proton-exchange-membrane fuel cells set by the U.S. Department of Energy.^356^

**Figure 27:**
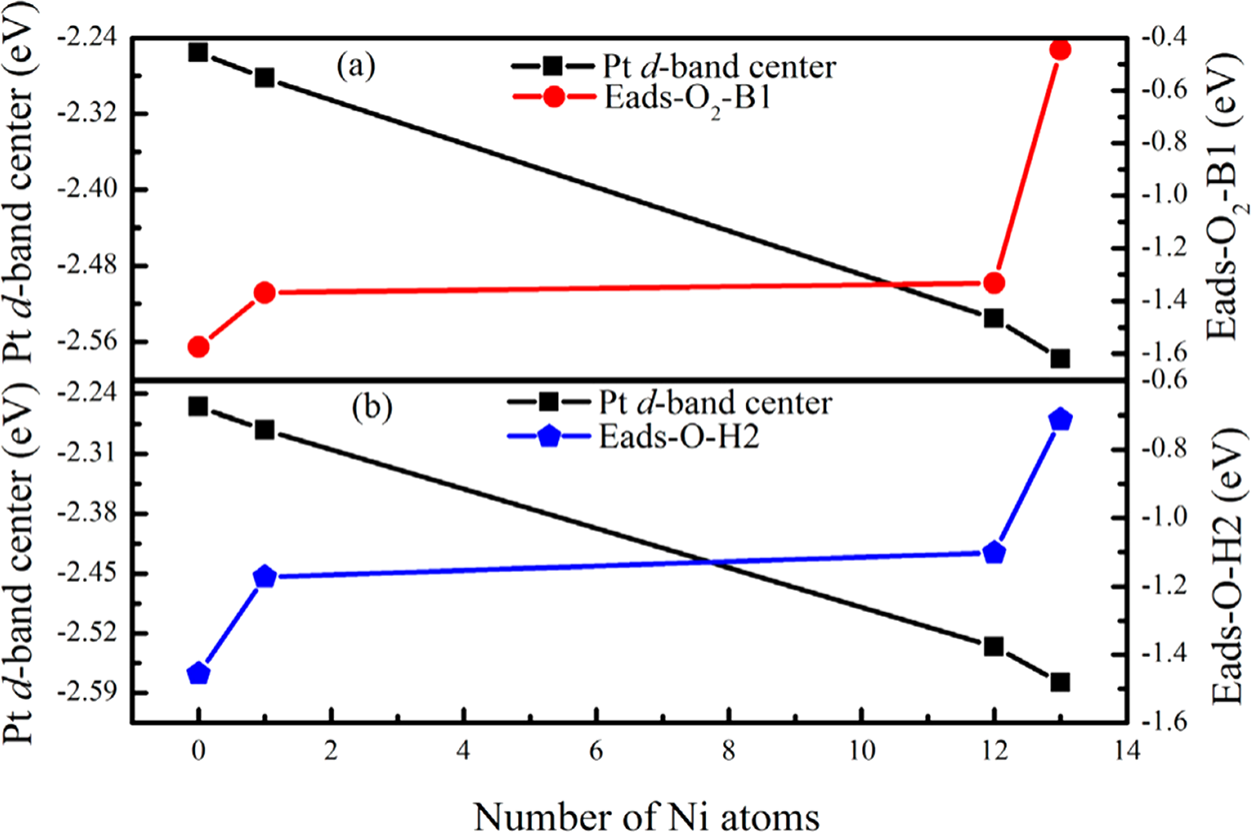
Pt d-band center and adsorption energies of O_2_ and O atoms in the Pd_13–*n*_Ni_*n*_@Pt_42_ (*n* = 0, 1, 12, 13) NPs. Adapted with permission from ref (357). Copyright 2017 Elsevier.

In Pd_13−n_Ni_n_@Pt systems, it appears that reactant adsorption is strongly affected by the coordination number of the shell atoms; the adsorption energy and d-band center were found to be inversely correlated with the activity, as shown in Figure 27. In general, the most active catalysts among those investigated were composed of NiPt; therefore, it was proposed that surface Pd impedes this reaction.^357^ For Pt@PdNi compositions, 0.8% compressive strain was observed in the structure compared with Pt_3_Pd, which aligned with EXAFS observations. The adsorption energy decrease was attributed to negative d-band shifts following the structural compression, which was further supported by a report that OH adsorption was 0.13 eV weaker than on the binary system.^358^

Pd–Pt based FCC TMNPs have been investigated as potential catalysts for scavenging of reactive oxygen species.^359,360^ Wang *et al.*(360) used experimental and DFT studies to investigate idealized PtPdRh FCC fragments with fixed structure and mixed compositions and showed that the O atoms are located further away from each other on the trimetallic structure compared with PtPd, which translated into remarkable scavenging efficiency for reactive oxygen species. Mu *et al.*(359) carried out an experimental study of nanozyme oxidation with additional computational modeling of a small PtPdMo FCC system (treated as a random alloy). The TMNPs in this study were reported to be attractive to electrons, thus capturing oxygen and nitrogen species on Mo, as confirmed by electrostatic potential and electron localization function analysis. Doping of PtPd with Mo caused lattice deformation and increased exposure of the (200) facets with highly active sites.

**Figure 28:**
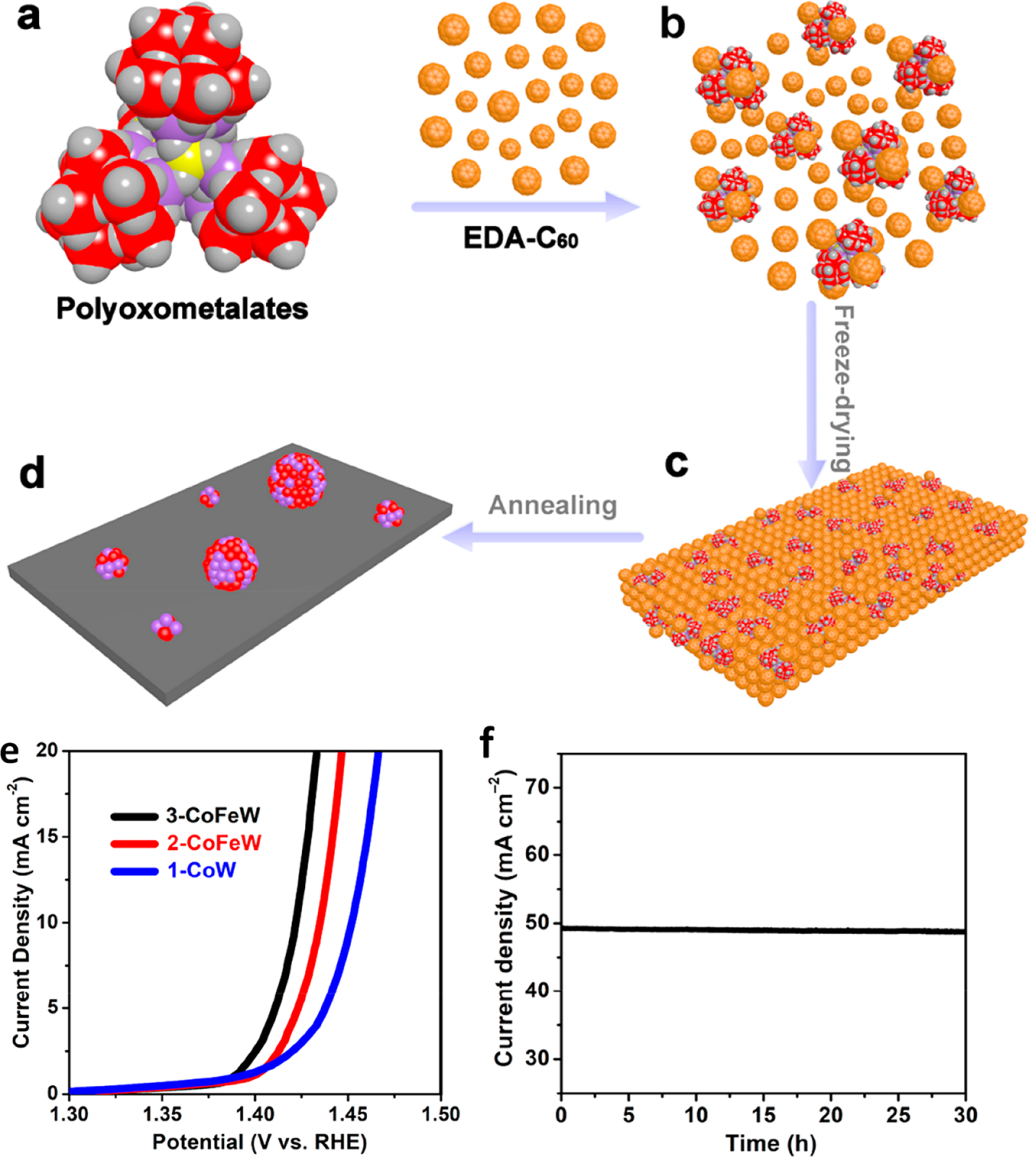
Schematic illustration of the synthesis of trimetallic clusters. (a) Space-filling representation of the structure of polyoxometalates (POMs). (b) Self-assembly of POMs and ethylenediamine-grafted C_60_ (EDA-C_60_) driven by electrostatic interactions. (c) Schematic structure of the POMs/EDA-C_60_ composites after freeze-drying. (d) Trimetallic clusters on a carbonic support. O, gray; Co/Fe, violet; W, red; P, yellow. (e) OER polarization curves of BNMP and TMNP catalysts loaded on gold foam (scan rate of 5 mV s^–1^ without *iR* correction). (f) Current density vs time for 3-CoFeW loaded on gold foam for 30 h at a constant applied potential of 1.46 V vs RHE for the OER. Adapted from ref (361). Copyright 2018 American Chemical Society.

Han *et al.*(361) investigated o-CoFeW TMNP catalytic materials for the oxygen evolution reaction (OER) (Figure 28), an important step of the water splitting reaction that can allow for clean and renewable energy production and utilization. o-CoFeW clusters were investigated by DFT using basin-hopping Monte Carlo for structural ordering. In the presence of Fe, the individual Co CN increases, suggesting that Co has a bias toward the core and Fe toward the surface of the o-CoFeW nanoclusters. The ternary CoFeW cluster structures result in desired intermediary adsorption energies of reactants, which were otherwise too strong (o-WFe) or too weak (o-WCo) in the binary metallic clusters that were examined. However, a degradation of catalytic activity was observed, which on the basis of experimental XPS data was attributed to the formation of an oxyhydroxide surface layer. o-CoFeW exhibited an overpotential of 192 mV at a current density of 10 mA cm^–2^ when loaded on gold foam as the working electrode (Figure 28), which places it among the most active oxygen evolution reaction catalysts reported to date.^361^

In summary, the overall outcome of the studies of the OER and ORR utilizing polymetallic electrocatalysts with well-defined surfaces or cluster shapes and composition can be a path to optimal catalyst design. A plethora of computational and experimental techniques can effectively be used together for screening and benchmarking of catalytic materials on the basis of a combination of electronic, morphological, and steric effects that translate into synergy for better catalytic performance. For example, enhancements in catalytic activity or durability can be achieved through interplay between two contrasting weak–strong interactions of intermediates with atomic species present in a TMNP, morphological changes, and/or better exposure of active sites. Well-balanced composition can help reach intermediate properties that allow better catalytic activity while increasing durability, and novel TMNP catalysts often manifest properties far superior to those of well-established commercially available materials.

### 4.5 Further Applications of TMNPs and Beyond Ternary Compositions

This section sets out further examples of TMNP-based catalysts that are not strictly applicable to a preceding section. Here TMNP catalysts have been applied to hydrodesulfurization (HDS) and dry reforming of methane (DRM). In both cases, achieving high activities is discussed with respect to the optimal catalyst formulation or preparative conditions of these examples, with the benefits of a ternary composition made clear for the outlined applications.

HDS is an industrial chemical process in which sulfur is removed during oil refining. Sulfur is the third most common element in crude oil and may account for up to 7.9% per gram.^362^ Reduction of sulfur-based emissions from the use of fuels is a key aspect of the current energy sector. Sulfur is also a common poison for precious metal catalysts, which are typically used further along in the refining process. Metal catalysts commonly involved in HDS are based on Ni, Co, Mo, and W,^363^ and in particular, sulfided versions of these catalysts have been found to be most active. The activity of sulfided catalysts is problematic because it can cause unwanted sulfur contamination; thus, over recent years an effort has been made to produce non-sulfided catalysts that are as active as their sulfided counterparts.^364^ A potential solution to the outlined challenge could lie in the application of trimetallic catalysts.

In a study reported by Mendoza-Nieto *et al.*,^129^ trimetallic NiMoW catalysts were tested for HDS of petroleum fractions, specifically dibenzothiophene and 4,6-dimethyldibenzothiophene. The catalysts were supported on either γ-Al_2_O_3_ or mesoporous SBA-15, which had been prepared with or without the addition of citric acid. The NiMoW/γ-Al_2_O_3_ catalyst was unaffected by the addition of citric acid during its preparation, and the metals were well-dispersed for the oxide and sulfided states regardless of how they were prepared. Analysis of TPR profiles suggested that a large fraction of Mo^6+^ and W^6+^ species with tetrahedral coordination were present, providing evidence of a strong metal–support interaction; consequently, the catalyst was not reducible at temperatures as high as 873–1273 K. Conversely, NiMoW/SBA-15 possessed a weak metal–support interaction, suggesting that agglomerated NiMoO_4_ and WO_3_ species were formed when the catalyst was prepared without the addition of citric acid. Through the addition of citric acid during preparation, the metal dispersion was increased, and the metals became more easily reduced and sulfided. Additionally, the presence of citric acid decreased the degree of stacking of the Mo(W)S_2_ active phase. The final citric acid-prepared NiMoW/SBA-15 catalyst had the highest activity for HDS of dibenzothiophene and 4,6-dimethyldibenzothiophene, as it was >2 times more active than NiMoW/γ-Al_2_O_3_.^129^

Mozhaev *et al.* assessed the impact of nickel addition to sulfided CoMo/Al_2_O_3_ catalysts for HDS of dibenzothiophene.^363^ The Mo loading was constant in each catalyst (10 wt %), and the Co and Ni contents were varied from 1.3 to 3.8 wt % and from 0.8 to 3.8 wt %, respectively. The catalysts were prepared by incipient wetness impregnation using decamolybdodicobaltate heteropolyacid (Co_2_Mo_10_HPA) and cobalt or nickel citrate. The catalysts were sulfided prior to the reaction, and catalytic testing took place in a fixed-bed reactor at 543 K and 3.0 MPa.

Increasing the Co:Mo ratio was found to decrease the TOF of the Co_*x*_–Co_2_Mo_10_/Al_2_O_3_ catalysts. The trimetallic Ni_*x*_–Co_2_Mo_10_/Al_2_O_3_ catalysts had higher TOF values than the bimetallic CoMo-based equivalents. These changes in activity were thought to be due to the formation of either mixed NiCoMoS active sites or, more likely, the coexistence of NiMoS and CoMoS active sites. However, it was crucial to have the correct ratio of metals: increasing the Co content in Co_*x*_–Co_2_Mo_10_/Al_2_O_3_ decreased the Co content in the CoMoS phase, causing an increase in the Co fraction in cobalt sulfide. Increasing the Ni content in Ni_*x*_–Co_2_Mo_10_/Al_2_O_3_, however, reduced the Ni content in the NiCoMoS phase while the Co percentage remained constant. At a (Co + Ni)/(Co + Ni + Mo) atomic ratio of 0.33, the dibenzothiophene HDS activity was observed to be at its maximum. The rate constant observed for the trimetallic sulfide catalyst was higher than that of the CoMoS/Al_2_O_3_ catalysts with the same metal content, and when the Ni loading was further increased to a Ni/(Ni + Co + Mo) molar ratio of 0.34, the TOF decreased because of a reduction in the number of mixed active sites.^363^

Liu *et al.*(365) used rare-earth metals in a trimetallic catalyst for the hydrocracking of jatropha oil to produce green diesel consisting of C_11_–C_20_ straight-chain alkanes. The catalysts were prepared by a novel powder metallurgy (PM) technique, which was advantageous when more common preparation techniques were problematic because of the significant effect of pH on the activity. In this preparation technique, γ-Al_2_O_3_ powder is mixed with the metal oxide precursor powders (5 wt % NiO, 15 wt % MoO_3_, and 1 wt % La_2_O_3_), pressed into a cylindrical shape, and then heat-treated at 973 K for 3 h. The preparation method is simple and inexpensive. For comparison, the same catalyst was also synthesized by a wet impregnation method followed by heat treatment at 400 °C. The catalysts were then tested for hydrocracking of jatropha oil in a fixed-bed reactor under the reaction conditions of 370 °C, 3.0 MPa, LHSV = 0.9 h^–1^, and H_2_/feed ratio = 1000/1.

Surface area analysis on the catalyst through nitrogen adsorption revealed that the pore diameter at the surface of the catalyst was smaller than the diameter inside the pore. Both the bare support and the catalyst prepared by impregnation showed a Type IV isotherm with an H4-type hysteresis loop, indicative of a mesoporous material. The PM catalyst showed a Type IV isotherm, with an H3-type hysteresis loop, suggesting that the preparation method affects the textural properties. Analysis of XRD patterns showed that there were no crystalline phases of MoO_3_ or LaO_3_ present, suggesting that these oxides are highly dispersed or amorphous. In the PM catalyst, which had been heated to 973 K, NiO phases were detected. The impregnation catalyst, which had been heated at 673 K, showed much less intense peaks, indicating that higher temperatures result in crystalline phases. TEM images showed very different results for the two catalysts, with the impregnation catalyst showing a rough surface with agglomerated metal species whereas the PM catalyst had a much smoother surface with a more homogeneous dispersion of metals.^365^

The catalyst prepared by PM had a higher selectivity for C_11_–C_20_ products. However, the jatropha oil conversion over the catalyst prepared by impregnation was higher, but more short-chain hydrocarbons and alkanes longer than 20 carbons were formed, which are less useful. Increasing the liquid hourly space velocity (LHSV) was found to decrease the conversion and significantly reduced the selectivity for C_11_–C_20_ alkanes because of a reduction in the contact time.^365^

Dry reforming of methane is of great industrial interest because it produces syngas (H_2_ and CO), which can be used for other applications, such as Fischer–Tropsch synthesis.^366,367^ Ni-based catalysts are among the more common materials used for DRM but often suffer from deactivation due to carbon deposition.^368−370^ Therefore, various strategies, such as alloying Ni with other metals, offer a solution to prevent sintering. The addition of Fe to form NiFe alloys has been reported to enhance carbon resistance and activity;^371^ however, the stability of the catalysts was a concern because of Fe segregation during the process of DRM. The NiFe alloy catalysts are sensitive to dealloying during the process and subsequently sinter.^372^ Theofanidis *et al.* reported the synthesis of a NiFePd alloy catalyst *via* incipient wetness impregnation^373^ that had enhanced stability and activity achieved through the addition of Pd to FeNi/MgAl_2_O_4_, wherein an FeNi core and a trimetallic shell composed of a FeNiPd alloy were formed. With an optimal Ni:Pd molar ratio of 75:1, DFT calculations and catalytic performance studies indicated that the addition of Pd to FeNi reduced the tendency of Fe to segregate and controlled the carbon formation, resulting in a high activity of 24.8 mmol s^–1^ g_metals_^–1^ after 21 h on stream (1023 K, 0.1 MPa). With use of other non-noble-metal additives, Jin *et al.* introduced Cu into NiFe alloy-based catalysts supported on MgAl_2_O_4_*via* coprecipitation to suppress Fe segregation and NP sintering.^374^ The effect of different Cu loadings was evaluated with DRM, and the subsequent catalytic performance was determined. The initial methane conversion as a function of time on stream followed the trend Ni_3_Cu_1_-MA > Ni_3_Fe_1_Cu_0.5_-MA > Ni_3_Fe_1_Cu_1_-MA ≈ Ni_3_Fe_1_-MA > Ni_3_Fe_1_Cu_0.5_-MA. Although Ni_3_Cu_1_-MA showed high activity initially, it severely deactivated over time; after 20 h, the methane conversion followed the trend Ni_3_Fe_1_Cu_0.5_-MA > Ni_3_Fe_1_-MA > Ni_3_Fe_1_Cu_1_-MA ≈ Ni_3_Cu_1_-MA > Ni_3_Fe_1_Cu_1.5_-MA. The trend at 20 h indicates that adding small amounts of Cu to form a trimetallic system enhanced activity and stability, which was correlated to an alloy structure formation according to EXAFS analysis. Excessive Cu addition, however, led to lower activity because Cu obscured the Ni active sites.

It is important to note that multimetal systems are not limited to trimetallic ones. As mentioned previously, incorporating multiple metals into a catalytic system increases the number of degrees of freedom, complicating preparative control and subsequent material characterization. High-entropy alloys (HEAs) are an example of systems that use multiple metals and are notably more complex than trimetallic systems. Yeh *et al.* first proposed HEAs as materials containing five or more elements in near equiatomic percentages.^375^ The entropic contribution to the total free energy overcomes the enthalpic contributions as the number of elements increases in an alloy, stabilizing solid solutions.^375,376^ HEAs have shown enhanced properties, including increased mechanical strength and stability and enhanced corrosion and oxidation resistance.^376−378^ There is appeal in the flexibility of designing systems with multiple metals, but the control and rationale behind these complex structures remain a challenge. Although HEAs are a relatively new field, they have been seen in a range of applications such as aerospace engineering, biomimetic materials, hydrogen storage materials, and electromagnetic shielding materials.^376,378^

In line with the purpose of this review, attention should be placed on their catalytic applications. Understanding is limited because of the premature nature of HEAs, and most efforts in this field have been placed on enhanced mechanical properties. However, reports have shown the application of HEAs in electrocatalytic systems, such as the hydrogen evolution reaction (HER),^379,380^ the ORR,^379,381^ and ammonia decomposition.^382^ Successful synthesis methods that have been developed for HEAs include a carbothermal shock method,^383^ fast-moving bed pyrolysis,^379^ and ball milling.^384^

**Figure 29:**
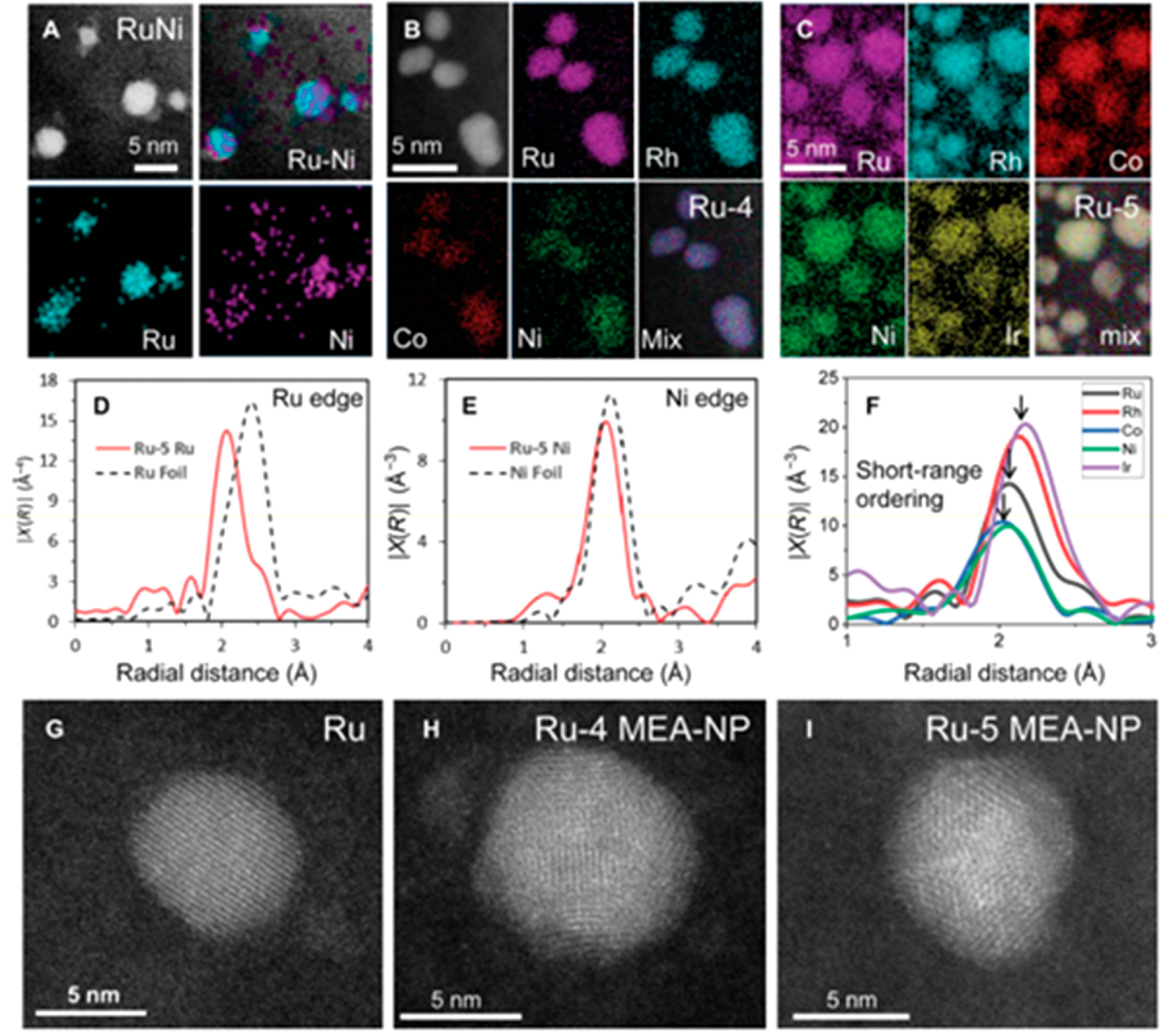
(A–C) Elemental maps of (A) bimetallic Ru–Ni NPs, (B) Ru-4 (RuRhCoNi), and (C) Ru-5 (RuRhCoNiIr) supported on carbon nanofibers. (D–F) FT-EXAFS spectra of (D) Ru in Ru-5, (E) Ni in Ru-5, and (F) Ru, Rh, Co, Ni, and Ir in Ru-5. (G–I) HAADF-STEM images of Ru, Ru-4, and Ru-5 MEA-NPs, respectively. From ref (385). CC BY-NC 4.0.

Multielemental alloy nanoparticles (MEA-NPs) can also be used to describe HEA systems. Yao *et al.* reported a computer-aided study to illustrate their promise for catalyst discovery, with vast compositional space.^385^ Alloy phase characterization in MEA-NPs was reported using EDX elemental maps, HAAD-STEM, and FT-EXAFS (Figure 29). Bimetallic Ru–Ni NPs show phase separation due to their immiscibility, which may be attributed to the Hume–Rother principle.^386^ However, when the number of elements is increased, a uniform distribution with a homogeneous alloy structure can be observed (Figure 29b,c). The alloy structure is also supported by FT-EXAFS measurements, as a slight shift was observed in the radial distance of Ru and Ni compared with their corresponding metallic bonds. Furthermore, in Figure 29f, a modest difference in bond lengths among the different elements present suggests that short-range ordering of the NPs exists.

Comparable to bimetallic and trimetallic catalysts, additional metals such as those found in HEAs can be beneficial as a result of synergistic effects. Facile synthesis methods are more commonly reported for bimetallic and trimetallic NPs in comparison with HEAs. Enhancing the relationship between theory-driven and experimental synthesis is crucial in the design, including the ability to prescreen a multitude of compositions. DFT, molecular dynamics, and Monte Carlo methods can aid the prediction of alloy formation and examine structural stability.^385^ To mass-produce HEAs efficiently, new synthesis strategies with low cost along with high catalytic performances should be thoroughly investigated to meet industrial needs and regulations.

Trimetallic systems already show challenges in terms of full characterization, but a multitude of studies, as presented in this review, have shown their catalytic enhancements through synergistic effects. HEAs require even more in-depth analysis, and to make progress, state-of-the-art characterization systems such as EXAFS, HAADF-STEM, and LEIS spectroscopy will be required to fully understand the properties of these multimetal systems.

## 5 Conclusions and Future Prospects

Reports on the preparation and use of TMNP catalysts have rapidly increased, and in this review these recent advances have been comprehensively discussed. Improvements in catalyst design in this field benefit from a combined computational and experimental approach because of the complexity of the supported and unsupported ternary nanoparticles. However, at this stage there are predominantly reports detailing novel preparation and particle compositions along with comparative catalyst testing studies of mono-, bi-, and trimetallic NPs. The elemental compositions of those discussed in the review often share common elements in combination, such as Pt, Au, Ni, Pd, Ag, Cu, Zn and/or Mo, Fe, and Co, other compositions include Rh, Ru, In, or Sn. Of these, the most successful or most often reported/applied compositions are often based on one or two of the following metals in combination or all three: Pd, Pt, and/or Au. This is the case because they readily form trimetallic alloys. These compositions have been applied as catalysts for both hydrogenation and oxidation reactions. Therefore, as a guide for future researchers hoping to use TMNPs, we offer that those preparations could involve two of these metals as a starting point.

Computational studies of TMNPs are increasing in parallel; however, there are only a handful of reports using a combined approach. The influence of a second or third metal on catalytic activity is often presented as a synergistic relationship and is strongly dependent on the nanoparticle composition, as evidenced with AuPdPt-based catalysts for the direct synthesis of hydrogen peroxide or the electrooxidation of ethanol. Despite major progress in studying TMNP compositions, there are great challenges, such as characterizing and understanding the particle makeup and inconsistencies from particle to particle. Furthermore, several reports detail a compromise between selectivity and activity, that is, some TMNPs may offer lower overall activity but higher selectivity, thus making them superior overall. The improved selectivity can be observed through steric hindrance, suppression of the formation of byproducts, electronic effects promoting the formation of product over a byproduct, or inhibition of unwanted side reactions by the third metal.

We have presented a mixture of articles, some focusing on materials and design whereas others directly compare catalytic activities of mono-, bi-, and trimetallic systems. Our aim in this review has been to bridge these aspects and give an overview of how each factor plays a role, particularly with respect to efforts to add non-noble metals to mono- or bimetallic NPs; this approach has proven to be more effective and economical, driving commercial attention. Advanced characterization, such as *operando* studies, may pave the way to a clear relationship of precise composition and structure with catalytic performance. Here computation can provide an essential insight in the design of catalysts and optimize influential factors on the reactivity, such as the d-band center, which themselves play a role in catalytic performance. However, challenges remain in exploring the full compositional spectrum and modeling reactive processes on realistic nanoparticles. When appropriate complementary characterization using bulk and surface spectroscopy, for example XPS, LEIS spectroscopy, surface-enhanced Raman spectroscopy, EXAFS and XANES, X-ray diffraction, high-resolution electron microscopy, NMR spectroscopy, and chemisorption should be used to discriminate and locate the metals within the NPs. The key information that we consider for researchers hoping to understand these systems starts with the composition and morphology of the nanoparticles, the chemical or electronic nature of the surface, support influences (if applicable), and finally detailed data on the mechanism of a given process. These key elements combined should provide sufficient information to relate the influence of a third metal to the experimental data. Following the techniques discussed by Ferrando *et al.*(17) on characterizing nanoalloys or BMNPs, we expect advances in resolution and modeling of ternary particles to greatly improve in the next 10 years, although effort is still necessary to unite computational modeling with materials and experimental design, particularly for supported systems. However, computational studies can already provide a wealth of information on potential for metals to alloy, phase composition, and surface electronic trends. On the basis of the results surveyed in this review, there is a wide scope for further research, principally in fundamental studies and development of TMNPs as catalysts, as the evident benefits will be applicable to other applications.
